# Antiaromaticity‐Driven Enhancement of Single‐Molecule Electrical Conductance by *s*‐Indacene Molecular Bridges

**DOI:** 10.1002/anie.3299896

**Published:** 2026-06-12

**Authors:** Ching‐Piao Chu, Yuki Hanamura, Tatsuhiko Ohto, Seiya Maeda, Ryo Yamada, Siao‐Lei Yu, Yen‐Ju Cheng, Hirokazu Tada, Yoshito Tobe

**Affiliations:** ^1^ Department of Applied Chemistry National Yang Ming Chiao Tung University Hsinchu Taiwan; ^2^ Graduate School of Engineering Science The University of Osaka Toyonaka Japan; ^3^ Graduate School of Engineering Nagoya University Nagoya Japan; ^4^ Center For Emergent Functional Matter Science National Yang Ming Chiao Tung University Hsinchu Taiwan

**Keywords:** antiaromaticity, aromaticity, HOMO–LUMO gap, molecular electronics, single‐molecule conductance

## Abstract

High electrical conductance of antiaromatic molecules compared with aromatic counterparts at the single‐molecule level has been a topic of controversy, with only a few previous experimental proofs. We synthesized length‐matched molecular wires incorporating 12 π‐electron antiaromatic *s*‐indacene (Ind) and 14 π‐electron aromatic benzodithiophene (BDT) cores with different anchoring modes and measured the single‐molecule electrical conductance (SMEC) by scanning tunneling microscopy‐break junction method, validating that antiaromatic cores significantly enhance the conductance. For the diagonally anchored systems, the antiaromatic Ind derivative exhibits SMEC approximately 10 times higher than that of its aromatic BDT analogue, thanks to the cooperative effects of a linear conjugation pathway and the narrow gap between the highest occupied molecular orbital (HOMO) and the lowest unoccupied molecular orbital (LUMO) that brings favorable frontier molecular orbital (FMO) alignment with the electrode Fermi level. Notably, for the horizontally anchored systems, the Ind derivative exhibits SMEC 2.8 times higher than that of its BDT counterpart, as its antiaromaticity‐driven FMO alignment overtakes the cross‐conjugation pathway. Additionally, introduction of electron‐withdrawing trifluoromethyl groups to the Ind core shifts the LUMO closer to the Fermi level, resulting in 1.5 times higher conductance. These results definitively demonstrate the enhancement of SMEC driven by antiaromaticity.

## Graphical abstract

Within the same molecular length scale, antiaromatic *s*‐indacene exhibits a marked increase in conductance compared to its aromatic benzodithiophene counterpart. Notably, the inherently small HOMO–LUMO gap of antiaromatic systems maintains a certain level of conductance even in the presence of cross‐conjugation. By exploiting molecular antiaromaticity and precise orbital engineering, this study introduces a transformative approach to developing next‐generation, high‐performance molecular wires.

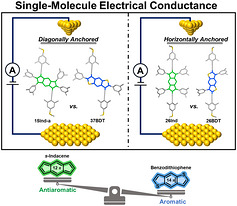

## 1 Introduction

Single‐molecule electrical conductance (SMEC) is a fundamental property in molecular electronics, offering insights into charge transport at the quantum level and paving the way for next‐generation molecular‐scale electronics [1, 2, 3, 4, 5]. It represents the ability of a single molecule to transport electrical current when connected to metallic electrodes with atomic scale precision. Experimental techniques such as scanning tunneling microscopy‐break junction (STM‐BJ) and mechanically controlled break‐junction (MCBJ) methods have been extensively used to measure electrical conductance (*G*) of single‐molecule junctions [6, 7, 8, 9]. These methods involve the formation of molecular junctions by trapping a molecule between mechanically manipulated electrodes and measuring the resulting current flow. The value of *G* depends on intrinsic molecular properties, such as molecular structures, electronic configurations, and charges, as well as extrinsic factors, including electrode materials and atomic‐level details of the molecule‐electrode contacts [10, 11]. Major efforts in this field have been focused on quantifying *G* for precisely tailored molecules to unravel charge transport mechanisms through structure‐property relationships [3, 4, 12]. Recently, molecular wires with high *G* were achieved using unconventional molecular bridges such as cumulenes [13, 14, 15], open‐shell diradicaloid molecules [16, 17, 18, 19, 20], and radical ions [21, 22], which exhibit significant increase in *G* relative to their closed‐shell counterparts. Despite the high efficiencies, the open‐shell bridge units would suffer from the drawback of low robustness due to the intrinsic reactivity of (di)radicals toward environmental dioxygen molecules, even if they are persistent during *G* measurements. Therefore, there remains a strong demand for bridge molecules featuring both high stability and high *G*. Antiaromatic compounds are among the potential candidates, as originally proposed by Breslow [23, 24], principally due to the narrow highest occupied molecular orbital (HOMO)–lowest unoccupied molecular orbital (LUMO) gaps, if they are reasonably stabilized by steric hindrance and/or benzo‐fusion, although this remains a topic of controversy. Herein, we present a definitive demonstration of enhanced *G* through antiaromatic *s*‐indacene bridges and its tunability through modulation of the MO energy levels by introduction of functional groups.

**FIGURE 1:**
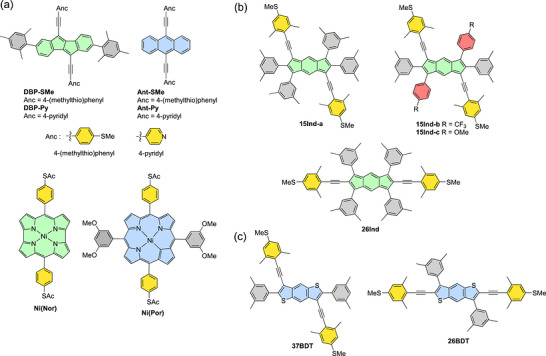
(a) Representative antiaromatic compounds **DBP‐SMe**, **DBP‐Py**, and **Ni(Nor)** and their aromatic counterparts **ANT‐SMe**, **ANT‐Py**, and **Ni(Por)** investigated for SMEC. (b) Structures of antiaromatic *s*‐indacenes **15Ind‐a**, **15Ind‐b**, **15Ind‐c**, and **26Ind**, and (c) aromatic benzodithiophenes **37BDT** and **26BDT**.

In contrast to the abundant use of aromatic compounds for SMEC experiments, antiaromatic compounds have been scarcely investigated due to the limited accessibility and stability, despite Breslow's hypothesis [23, 24]. In this respect, the impact of antiaromaticity on SMEC has begun to attract significant attention; nevertheless, there are few studies that definitively confirm the advantage of antiaromaticity. Initially, the hypothesis was supported indirectly by the observation that *G* decreases with increasing aromaticity in a series of conjugated five‐membered ring compounds [24, 25, 26]. Subsequent reports do not support a clear (anti)aromaticity‐*G* relation. For example, heteroaromatic compounds of aromatic [27, 28, 29] or antiaromatic character [30] were revealed to show no aromaticity‐related differences in SMEC, either experimentally or theoretically. More recently reported antiaromatic compounds shown in Figure S1 do not critically demonstrate the effects of antiaromaticity either, except for a few instances shown in Figure 1a. Specifically, an antiaromatic dibenzo[*a,e*]pentalene equipped with thiomethyl anchor groups (**DBP‐SMe**) exhibited conductance comparable to that of the aromatic anthracene derivative **ANT‐SMe** (1.3 times) [31]. Moreover, the pyridine‐anchored derivative **DBP‐Py** showed an appreciable increase relative to **ANT‐Py** (1.7 times) [32]. The difference was explained in terms of the different frontier molecular orbitals (FMOs) involved in charge transport—the HOMO in **DBP‐SMe** and the LUMO in **DBP‐Py**—highlighting the importance of the energy gap between the relevant MO and the electrode Fermi level. However, even in **DBP‐Py**, the impact of antiaromaticity on SMEC was not remarkable. A more pronounced increase in *G* was observed for the Ni(II)‐norcorrole complex (**Ni‐Nor**), an antiaromatic metal complex featuring a 16 π‐electron macrocycle, which exhibits a *G* value approximately 25 times higher than its aromatic analog, the 18 π‐electron Ni(II)‐porphyrin complex (**Ni‐Por**) [33]. This enhancement was attributed to the LUMO level of **Ni‐Nor** being closer to the electrode Fermi level than that of **Ni‐Por** due to its antiaromatic electronic configuration. However, the difference may also be partially attributed to other factors, such as different interjunction distances and conformational difference of the macrocycles. In this manner, a straightforward demonstration of the impact of antiaromaticity on enhanced SMEC compared with aromatic counterpart under comparable conditions remains lacking, although it is not an easy task to perform. Herein, we report SMEC measurements using an antiaromatic 12 π‐electron hydrocarbon, *s*‐indacene, as the bridge molecules to verify this hypothesis by detailed comparison.

For this purpose, we envisioned that *s*‐indacene is a suitable building block because of the following features: (1) moderate antiaromaticity as indicated by the nucleus‐independent chemical shift (NICS) [34] values (+21.6 at the five‐membered rings and +17.6 at the six‐membered ring for the *C*_2_ *_h_* geometry at the B3LYP/6‐31G(d) level) [35, 36]. (2) Kinetic stability sufficient not only for steady *G* measurements but also as robust device components [34, 35]. (3) A non‐uniform FMO distribution with near degenerate HOMO and HOMO−1 energy levels, enabling modulation by site‐specific substitution [36, 37, 38, 39], (4) versatile functionalization based on the established method, allowing precise substitution at designated positions [36], and (5) the high potential for π‐electron delocalization, theoretically estimated based on the inter‐fragment delocalization index [40].

Based on these considerations, we designed two *s*‐indacene derivatives, **15Ind‐a** and **26Ind**, wherein the thioxylenyl anchor groups attached through a triple‐bond linkage at either the C1/C5 or C2/C6 positions of the *s*‐indacene core, respectively (Figure 1b). To enable a rigorous assessment of the impact of antiaromaticity, we synthesized the corresponding aromatic benzodithiophene analogues, **37BDT** and **26BDT**, which possess 14 π‐electrons (Figure 1c). The S─S distances in diagonally anchored **15Ind‐a** and **37BDT** are presumed to be nearly identical to each other as are those in horizontally anchored **26Ind** and **26BDT**, although the distances between the diagonal and horizontal anchoring types would be slightly different. To elucidate the substituent effects on *G*, we also synthesized **15Ind‐b** and **15Ind‐c**, which possess electron‐withdrawing or electron‐donating substituents, respectively. Using the STM‐BJ technique, we measured the single‐molecule *G* of these compounds at room temperature. Our results demonstrate that the diagonally anchored antiaromatic **15Ind‐a** exhibits significantly higher single‐molecule *G* relative to its aromatic **37BDT**. Similarly, in the horizontally anchored systems, the antiaromatic **26Ind** exhibits a higher *G* value than that of the aromatic **26BDT**, although the difference is small. Furthermore, electronic modulation of the **15Ind** core enables tuning of the conductance, with **15Ind‐b** exhibiting higher conductance than **15Ind‐a**.

To corroborate the experimental results and gain deeper mechanistic insights, these measurements were complemented by theoretical calculations based on the nonequilibrium Green's function method combined with density functional theory (NEGF‐DFT). This comparative experimental and theoretical approach allows for a direct evaluation of how core aromaticity versus antiaromaticity dictates charge transport efficiency.

## 2 Results and Discussion

### 2.1 Synthesis and Characterization of Bridge Molecules

**SCHEME 1:**
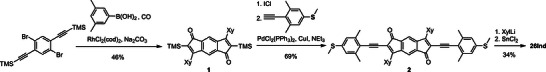
Synthesis of antiaromatic bridge molecule **26Ind**.

The synthesis of **15Ind‐a**, **15Ind‐b**, and **15Ind‐c** was carried out using the same method [36] as reported previously (Scheme S1). The synthesis of **26Ind** deserves mention, regarding a new approach to diketone **1** developed utilizing the Rh‐catalyzed carbonylative cyclization [41] (Scheme 1). The scope and limitation of this approach will be reported elsewhere. Subsequently, the attachment of the anchor groups followed by usual transformations gave **26Ind**. The aromatic analogs **37BDT** and **26BDT** were prepared using the known procedures (Schemes S3 and S4). Detailed synthetic procedures are described in the Supporting Information.

**FIGURE 2:**
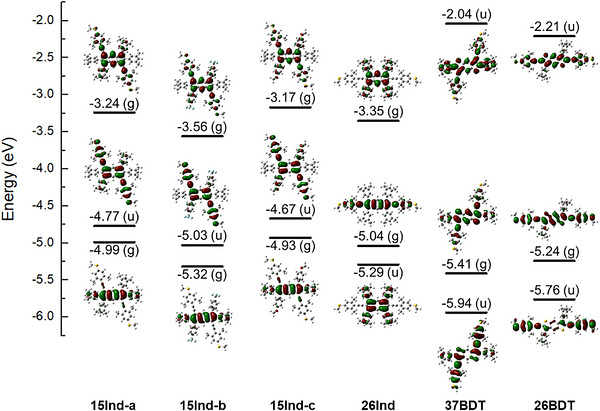
FMO diagrams. To differentiate the symmetry types of the MOs, the signs (g) and (u) are used to denote *gerade* and *ungerade* distribution of wave functions in the *s*‐indacene core, respectively. Note that the sequences of the HOMO and HOMO−1 of **15Ind‐a**, **15Ind‐b**, and **15Ind‐c** are inverted compared with **26Ind**.

The FMO energy levels and distributions were evaluated by DFT calculations at the B3LYP/6‐311G(d,p) level of theory because of the satisfactory performance in reproducing molecular geometries and MO energy levels. The theoretical FMO levels, HOMO−LUMO gaps (Δ*E*_gap1_), and (HOMO−1)−LUMO gaps (Δ*E*_gap2_) are summarized in Table S1 together with the experimental orbital levels estimated by cyclic voltammetry (CV). The MO diagrams (Figure 2) show a clear dependence of FMO energy levels and orbital distributions on the position of the anchor groups. Antiaromatic **15Ind** system with the anchor groups at C1 and C5 exhibits HOMO of *ungerade* (u) symmetry type MO with significant orbital distribution on the anchor groups, as does the LUMO, which is of *gerade* (g) symmetry. **15Ind‐a** shows a HOMO–LUMO gap (Δ*E*_gap1_) of 1.53 eV. Notably, the HOMO of **26Ind** with anchors on C2 and C6 is of *gerade* (g) symmetry with significant wavefunction distribution on the anchor groups, whereas the LUMO exbibits negligible distribution, although it belongs to the same *gerade* symmetry type. As a result of the symmetry switch between HOMOs of **15Ind‐a** and **26Ind**, **26Ind** exhibits a slightly larger Δ*E*_gap1_ (1.69 eV) compared to **15Ind‐a**. In marked contrast, Δ*E*_gap1_ values of aromatic **37BDT** and **26BDT** (3.37 eV and 3.03 eV, respectively) are much larger than those of **15Ind‐a** and **26Ind**.

To confirm the theoretical trend in the HOMO and LUMO energy levels, CV measurements in CH_2_Cl_2_ were conducted. The voltammograms are shown in Figure S2. While the **15Ind** derivatives and **26Ind** exhibit reversible reduction and irreversible oxidation waves, both reduction and oxidation of **37BDT** and **26BDT** were irreversible. The HOMO and LUMO levels, determined based on the onset potentials, are compiled in Table S1. The experimentally estimated HOMO and LUMO energy levels of **15Ind‐a** and **26Ind** were −5.05/−3.93 eV and −5.10/−3.95 eV, respectively, leading to the corresponding energy gap (*E*_g_^ele^) values of 1.12 and 1.15 eV, respectively. For aromatic **37BDT**, the HOMO and LUMO levels were estimated to be −5.25 and −2.95 eV, respectively (*E*_g_^ele^ = 2.30 eV). In contrast, **26BDT** displays a similar HOMO level of −5.23 eV and a lower‐lying LUMO level of −3.02 eV, resulting in a smaller bandgap of 2.21 eV. The trend in the CV‐based MO levels is consistent with that of the theoretical levels, validating the theoretical estimates shown in Figure 2.

**FIGURE 3:**
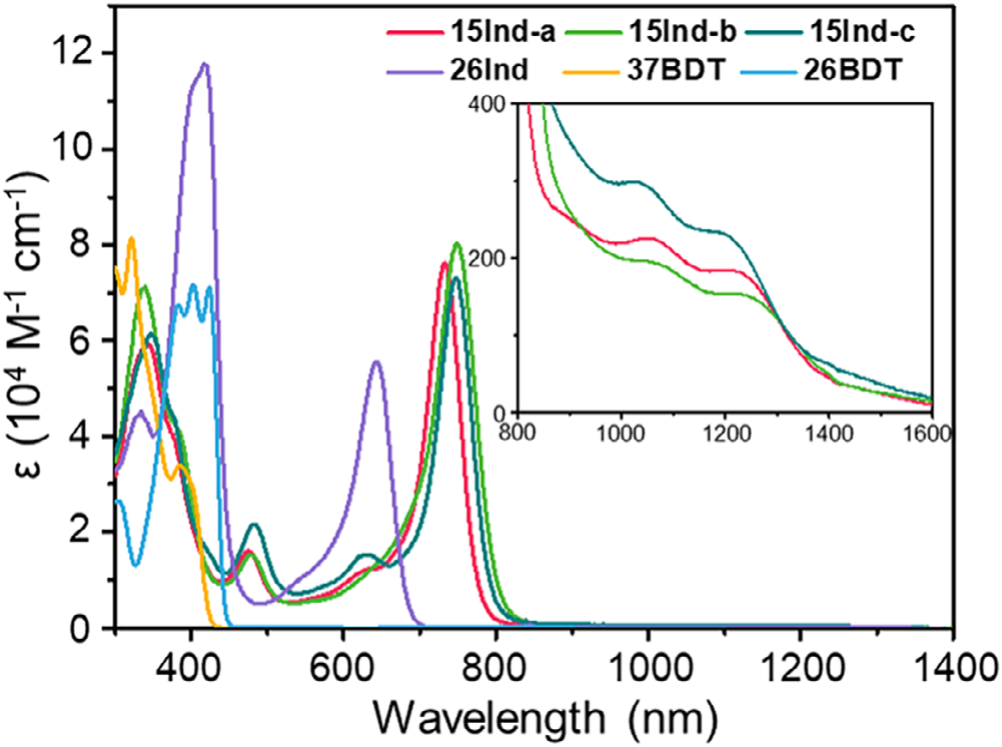
Absorption spectra of bridge molecules in CH_2_Cl_2_. Due to low solubility, the absorption spectrum of **26Ind** at the NIR region was not measured.

The MO energetics of the molecular bridges are also reflected in the absorption spectra measured in CH_2_Cl_2_ at 298 K (Figure 3). The absorption wavelengths and extinction coefficients are summarized in Table S2, together with the assignments of the bands based on time‐dependent density functional (TD‐DFT) calculations (Tables S3–S9). The **15Ind‐a** exhibits a significant bathochromic shift and a larger absorption coefficient in visible region compared with **26Ind**. These absorption bands are assigned to the HOMO (u) → LUMO (g) and HOMO–1 (u) → LUMO (g) transitions, respectively. Specifically, **15Ind‐a** shows an absorption maximum (*λ*_max2_) at 776 nm with an optical gap (*E*_g_^opt^) of 1.60 eV, whereas **26Ind** exhibits λ_max2_ at 644 nm. However, *E*_g_^opt^ for **26Ind** cannot be determined because its primary transition arises from the HOMO–1 (u) → LUMO (g) transition, rendering a direct comparison unfeasible.

The closed‐shell electronic configurations of **15Ind** derivatives and **26Ind** were confirmed by spin‐unrestricted DFT calculations, furnishing small diradical character indices (*y*_0_) evaluated using Yamaguchi's scheme [42] (Table S10). To confirm antiaromaticity of the **15Ind** derivatives and **26Ind**, we estimated the NICS(1)*_zz_* values—the component corresponding to the principal axis perpendicular to the ring plane of nucleus‐independent chemical shift evaluated at 1 Å above the plane— [34, 43, 44] together with the theoretical and experimental ^1^H NMR chemical shifts of the hydrogen atom located at the central ring of the indacene core (Table S11). For the five‐ and six‐membered ring centers, **15Ind‐a** exhibits the NICS(1)*_zz_* values of 33.6/23.0 ppm, respectively, whereas **26Ind** displays 35.0/26.7 ppm. With increasing NICS value, the chemical shift of the proton moves slightly upfield both theoretically and experimentally. However, in view of the small difference, the magnitude of antiaromaticity based on the magnetic criterion is regarded as approximately the same for all *s*‐indacene derivatives.

In single‐molecule junctions, the efficiency of charge transport is primarily governed by three key molecular and electronic characteristics: (1) the HOMO–LUMO gap, which dictates the energy alignment between the FMOs and the Fermi level of the metal electrodes, with a narrower gap generally reducing the energy barrier for electron tunneling and thereby enhancing molecular conductance [4, 12], (2) the inter‐anchor distance which defines the physical length of the tunneling path and primarily governs the exponential length‐dependent decay of conductance (*G *= *A*e^−^ *^βL^*: *β*, conductance decay constant; *L*, molecular length) [3, 45, 46, 47, 48], and (3) the conjugation mode (e.g., linear versus cross‐conjugation), which not only determines the degree of electron delocalization along the molecular backbone but also strictly modulates quantum interference effects and the resulting transmission pathways [4, 12, 49, 50, 51, 52]. Collectively, the intricate interplay of these three factors dictates the overall charge transport behavior of a single molecule.

In our study, the DFT calculations reveal that the diagonally anchored molecules **15Ind‐a** and **37BDT** exhibit nearly identical S─S distances of 2.13 Å and 2.11 Å, respectively, so do the horizontally anchored molecules **26Ind** (2.41 Å) and **26BDT** (2.42 Å). By effectively keeping the physical length of the tunneling path constant, we can rule out the influence of distance‐dependent decay on the measured conductance.

**FIGURE 4:**
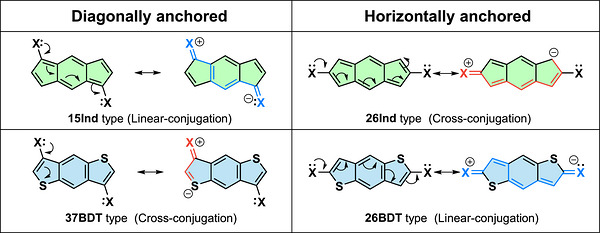
Conjugation pathways in diagonally and horizontally anchored Ind and BDT models.

For the conjugation modes, the possible resonance forms connecting the two anchor groups, represented by X, are illustrated using curly arrows (Figure 4) [53]. In **15Ind** and **26BDT** types, continuous conjugation pathways can be drawn between the anchors, defining a linearly conjugated π system. Conversely, **26Ind** and **37BDT** types are cross‐conjugated, with no continuous π pathway connecting the two anchoring groups. This cross‐conjugated topology induces destructive quantum interference (DQI), leading to significantly suppressed molecular conductance [4, 49, 50, 51, 52]. As a result, the effects of antiaromaticity and conjugation modes are expected to impact the SMEC either cooperatively or oppositely.

### 2.2 Single‐Molecule Electrical Conductance (*G*)

We utilized the STM‐BJ technique to create single‐molecule junctions of all compounds and subsequently measured their electrical conductance (*G)*. Detailed information for the measurements and data analysis is described in the Supporting Information. Briefly, a gold STM tip was repeatedly brought into contact with a gold substrate modified with the target molecules. Molecular junctions were formed when the contact between the gold tip and the gold substrate was broken during the tip retraction process. Steps or plateaus were observed in the conductance traces measured during tip retraction when single‐molecule junctions were formed. All measurements were carried out in an Ar atmosphere. We conducted measurement with bias voltages of 100, 200, and 500 mV (Figures S5 and S6; Table S12). No significant dependence of *G* on bias voltage was observed. In the following discussion, *G* values at a bias voltage of 200 mV are used because clear plateaus for **37BDT** could not be observed at a bias voltage of 100 mV due to its small conductance.

**FIGURE 5:**
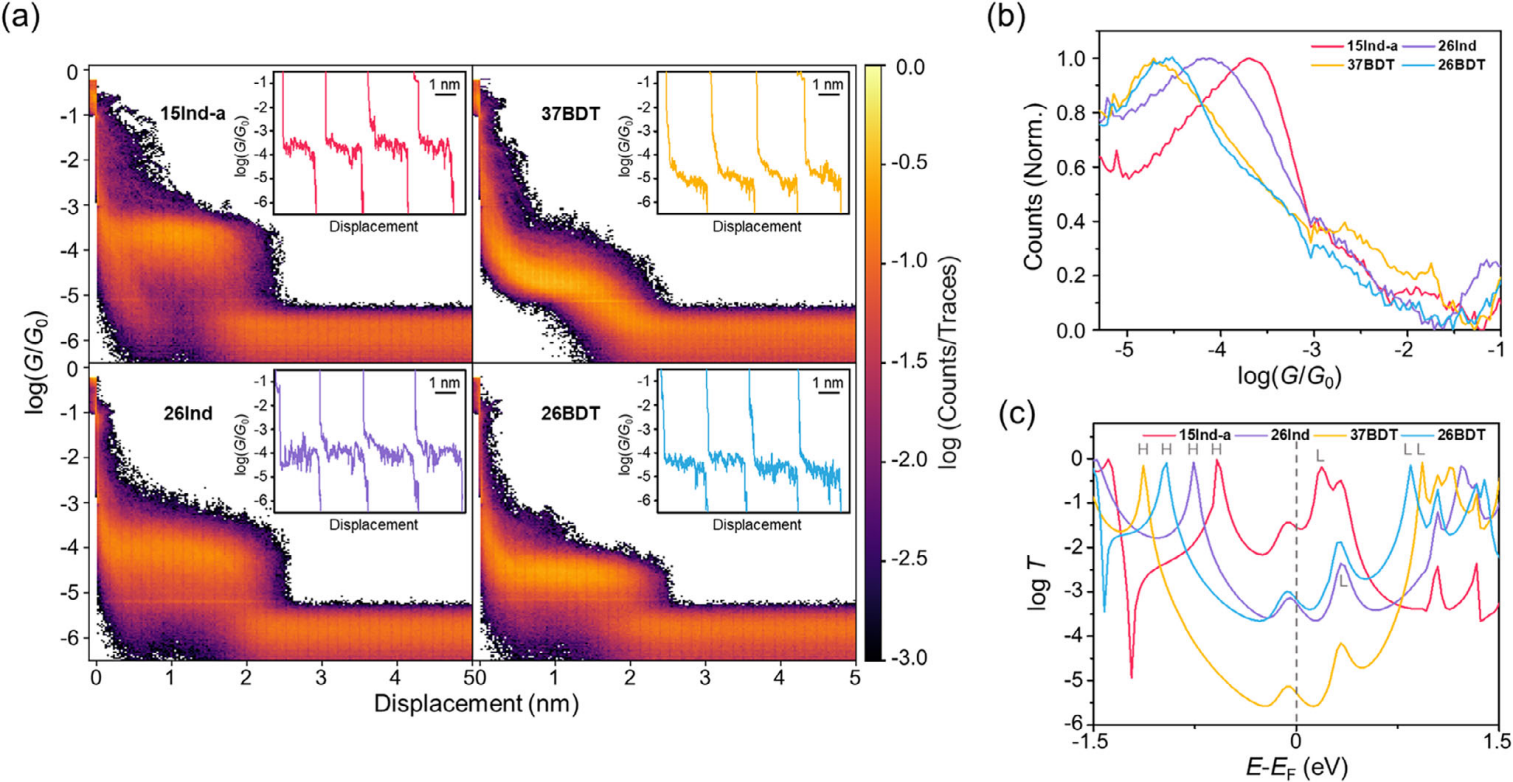
Results of STM‐BJ experiments for **15Ind‐a** (1299 traces), **26Ind** (1548 traces), **37BDT** (1373 traces), and **26BDT** (1119 traces). (a) 2D conductance histograms with conductance traces shown in the inset. (b) 1D conductance histograms. (c) Spectra of calculated zero‐bias transmission (*T*), where H and L (shown in gray) denote the HOMO and LUMO, respectively. The Fermi level (*E*_F_) is indicated by the gray dashed line at 0 eV.

The formation of plateaus is also evident in the 2D histograms for the four compounds (Figure 5a). In the 2D histogram of all compounds, there are significant distributions of *G* extended to ca. 2 nm followed by the abrupt change to the background signal level (log (*G*/*G*_0_) < −6), indicating the breaking of the single‐molecule junctions. The plateau lengths are estimated from the measured lengths by addition of a snap‐back distance of 0.5 nm [47, 54]. Specifically, the horizontally anchored configurations (**26Ind** and **26BDT**) exhibit experimental plateau distances of ca. 2.5 nm, whereas for the diagonally anchored counterparts (**15Ind‐a** and **37BDT**) the corresponding distance is ca. 2.2 nm. These distances are in agreement with the S─S distance estimated for each type of compounds (Table 1). The *G* values for each compound were determined by fitting a Gaussian to the conductance peak in the one‐dimensional (1D) histogram (Figure 5b) because the *G* distribution assigned as single‐molecule junctions in the 2D histogram corresponds to the peak in the 1D histogram. We obtained the most probable conductance (log(*G*/*G*_0_)), where *G*_0_ = 2*e* ^2^/*h* (*e*: elementary charge and *h*: Planck's constant) (Table 1). The antiaromatic **15Ind‐a** exhibits a value of −3.66 (2.2 × 10^−4^ *G*_0_), whereas its structural isomer **26Ind** shows a lower value of −4.14 (7.2 × 10^−5^ *G*_0_). For the aromatic BDT system, **37BDT** and **26BDT** exhibit conductance values of −4.68 (2.1 × 10^−5^ *G*_0_) and −4.58 (2.6 × 10^−5^ *G*_0_), respectively, which are smaller than those of the corresponding antiaromatic molecules with the same anchoring mode. These values clearly demonstrate that both (anti)aromatic core and anchoring mode have pronounced effects on charge transport, resulting in the approximate order **15Ind‐a** > **26Ind** > **26BDT** > **37BDT**. Despite the similar S─S distances, the diagonally anchored molecule **15Ind‐a** exhibits a conductance approximately 10 times higher than that of **37BDT**, whereas the horizontally anchored molecules **26Ind** shows a conductance about 2.8 times higher than that of **26BDT**.

**TABLE 1:** Single‐molecule electrical conductance and length data from STM‐BJ experiments of **15Ind‐a**, **26Ind**, **37BDT**, and **26BDT**

Compd	Measured *G* ^a^ (log(*G*/*G*_0_))	Theoretical *G* (log(*G*/*G*_0_))	Measured length (nm)	Measured length + 0.5^b^ (nm)	Theoretical S–S distance^c^ (nm)	*E*_g_^ele^ (eV)
**15Ind‐a**	−3.66 (0.85)	−1.54	1.70 ± 0.29	2.20	2.09 (2.13)	1.12
**26Ind**	−4.14 (1.79)	−3.25	1.99 ± 0.25	2.49	2.39 (2.41)	1.15
**37BDT**	−4.68 (0.98)	−5.26	1.67 ± 0.22	2.16	2.08 (2.11)	2.30
**26BDT**	−4.58 (0.91)	−3.16	1.99 ± 0.30	2.49	2.42 (2.42)	2.21

^a^ Values in parentheses are the full width at half maximum (FWHM) of the peak.

^b^ Sum of the measured length and snap‐back length of 0.5 nm.

^c^ Obtained from molecular junction structures optimized by DFT. Values in parentheses are obtained from DFT calculations for free molecules.

To further examine the influence of antiaromaticity on conductance, we first investigated the spatial distribution of the FMOs for each molecule chemically bonded to the gold electrodes by NEGF‐DFT calculations. As depicted in Figure S11, the antiaromatic **15Ind‐a** exhibits significant electron density on the anchor groups in both HOMO and LUMO. In contrast, for **26Ind**, although the HOMO displays significant density localized on the anchor groups, the LUMO density does not extend to these anchoring sites. For the aromatic series, both **37BDT** and **26BDT** show considerable orbital distributions on the anchor groups in both HOMO and LUMO. These results corroborate the MO distributions of free bridge molecules shown in Figure 2.

Following the FMO analysis, we calculated the zero‐bias transmission spectra for each molecule (Figure 5c). In these spectra, the gray dashed line indicates the Fermi level (*E*_F_) of the gold electrodes. The conductance of the molecular junction is determined by the transmission value at this Fermi level; consequently, a transmission peak which aligns closer to *E*_F_ and exhibits a higher magnitude corresponds to a higher molecular conductance. Our calculations reveal that a LUMO‐mediated channel is primarily responsible for charging transport for antiaromatic *s*‐indacene molecules, as determined by the orbital whose energy level lies closest to the Fermi level.

The theoretical conductance values (log(*G*/*G*_0_)) from these spectra are calculated to be −1.54 for **15Ind‐a**, −3.25 for **26Ind**, −5.26 for **37BDT**, and −3.16 for **26BDT**. The calculated trend qualitatively agrees with our experimental observations. Similar to the theoretical calculation in vacuum (Figure 2), antiaromatic compounds generally exhibit a narrower HOMO–LUMO gap. As shown in Figure 5c, both antiaromatic **15Ind‐a** and **26Ind** have smaller energy gaps than the aromatic BDT series, with both HOMO and LUMO levels located closer to the Fermi level. However, the transmission spectrum of **26Ind** indicates the significantly suppressed, nearly vanishing LUMO peak near the Fermi level (Figures S8 and S9). This suppression is attributed to the negligible LUMO density on its sulfur anchor groups, which humpers efficient LUMO‐mediated charge transport and contributes to the lower conductance than that of **15Ind‐a**. As can be seen in Figure S11, because there is little difference between the bonding geometries of the sulfur atoms to the gold electrodes, the atomic level details of molecule‐electrode contact are regarded to be the same. Moreover, considering the relatively small *y*_0_ values, it is unlikely that the difference between the open‐shell character plays a significant role to the different conductance, although the *y*_0_ value of **15Ind‐a** is larger than that of **26Ind** (Table S10).

Despite the apparent absence of a strong LUMO transmission channel, the calculated conductance of **26Ind** is comparable to that of the aromatic **26BDT**. To elucidate the reason for this result, the transmission spectrum near *E*_F_ was magnified (Figure S10). This closer inspection reveals that a significant contribution from the HOMO channel becomes accessible. Although the primary LUMO transmission is weak due to poor orbital overlap at the anchoring sites, the intrinsically small HOMO–LUMO gap of the antiaromatic core places the HOMO sufficiently close to the Fermi level, allowing it to contribute to the transmission and thereby enhancing the conductance for **26Ind**. In contrast, the aromatic BDT derivatives, **37BDT** and **26BDT** (Figure 5c), generally exhibit larger HOMO–LUMO gaps, which shift the FMOs father from the Fermi level and lead to lower transmission values and consequently lower calculated conductance.

By integrating these electronic and topological analyses, we can comprehensively rationalize the significant conductance contrasts observed in our experimental data. For the diagonally anchored systems, the antiaromatic **15Ind‐a** intrinsically features a narrow HOMO–LUMO gap that aligns its FMOs closely with the Fermi level, as well as a linearly conjugated pathway that facilitates highly efficient charge transmission. Under the cooperative effects of these two favorable characteristics, the conductance of **15Ind‐a** significantly exceeds that of **37BDT**. On the other hand, for the horizontally anchored counterparts, the superior conductance of **26Ind** over **26BDT** arises from the competing effects of electronic and topological origin. Specifically, the impact of narrow HOMO–LUMO gap predominates over the effect of cross‐conjugation, serving as the dominant contributor to the enhanced conductance.

Next, as a preliminary study of FMO modulation effects, we replaced the two xylyl substituents of **15Ind‐a** with either electron‐withdrawing 4‐(trifluoromethyl)phenyl group (**15Ind‐b**) or electron‐donating 4‐methoxyphenyl group (**15Ind‐c**) to explore whether such modifications enable systematic tuning of the conductance magnitude, in view of previous studies on substituent effects [55, 56, 57, 58]. The synthetic details of **15Ind‐b** and **15Ind‐c** are provided in Supporting Information. As shown in Figure 2, DFT calculations indicate that introduction of electron‐withdrawing trifluoromethyl group significantly lowers both the HOMO and LUMO levels in **15Ind‐b** to −5.03 eV and −3.56 eV, respectively, resulting in a reduced Δ*E*_gap1_ of 1.47 eV. In contrast, an electron‐donating methoxy group, as in **15Ind‐c**, raises the HOMO and LUMO levels to −4.67 eV and −3.17 eV, respectively, with a Δ*E*_gap1_ of 1.50 eV. The theoretical S─S distances of **15Ind‐b** (2.10 Å) and **15Ind‐c** (2.11 Å) are virtually identical with that of **15Ind‐a** (2.09 Å). Experimental CV results validate this trend, as shown in Table S1 and Figure S2. Specifically, **15Ind‐b** exhibits lowered HOMO/LUMO levels of −5.13/−4.10 eV (*E*_g_^ele^ = 1.03 eV), whereas **15Ind‐c** shows slightly elevated levels to −5.02/−3.92 eV (*E*_g_^ele^ = 1.10 eV). Consistently, in the absorption spectra, **15Ind‐b** shows the most red‐shifted λ_max2_ at 802 nm, corresponding to the smallest *E*_g_^opt^ of 1.55 eV, while **15Ind‐c** exhibits *λ*_max2_ at 790 nm with an *E*_g_^opt^ of 1.57 eV (Figure 3; Table S2).

**FIGURE 6:**
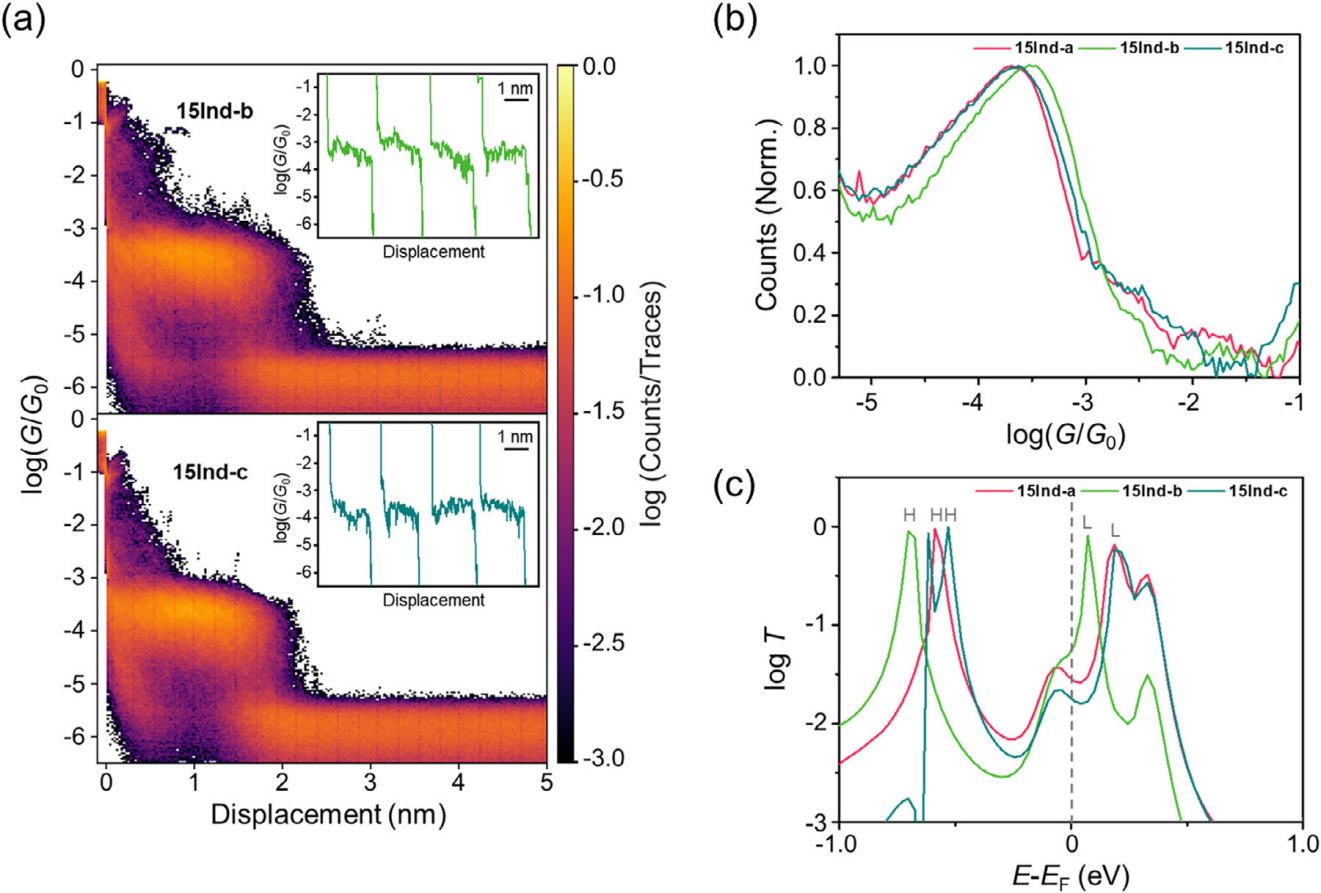
Results of STM‐BJ experiments for **15Ind‐b** (1220 traces) and **15Ind‐c** (1151 traces). (a) 2D conductance histograms with conductance traces shown in the inset. (b) Comparison of the 1D conductance histograms with that of 15Ind‐a. (c) Spectra of calculated zero‐bias transmission (*T*) compared with **15Ind‐a**, where H and L (shown in gray) denote the HOMO and LUMO, respectively. The Fermi level (*E*_F_) is indicated by the gray dashed line at 0 eV.

The 1D and 2D conductance histograms of **15Ind‐b** and **15Ind‐c** are summarized in Figure 6; Table 2. The 1D conductance histograms (Figure 6b) show a single conductance peak at approximately log(*G*/*G*_0_) = −3.49 (3.2 × 10^−4^ *G*_0_) for **15Ind‐b**, which exhibits the highest conductance among the three derivatives, whereas **15Ind‐c** displays a conductance value of log(*G*/*G*_0_) = −3.64 (2.3 × 10^−4^ *G*_0_), nearly identical with that of **15Ind‐a**. Thus, introduction of two electron‐withdrawing substituents leads to a clear enhancement of conductance, with **15Ind‐b** showing an increase of about 1.5 times relative to **15Ind‐a**. These results indicate that introducing electron‑withdrawing group to lower the FMO energy level effectively enhances the conductance.

**TABLE 2:** Single‐molecule electrical conductance and length data from STM‐BJ experiments of **15Ind‐b** and **15Ind‐c**

Compd	Measured *G* ^a^ (log(*G*/*G*_0_))	Theoretical *G* (log(*G*/*G*_0_))	Measured length (nm)	Measured length + 0.5^b^ (nm)	Theoretical S─S distance^c^ (nm)	*E*_g_^ele^ (eV)
**15Ind‐b**	−3.49 (0.94)	−1.25	1.61 ± 0.29	2.11	2.10 (2.11)	1.03
**15Ind‐c**	−3.64 (1.01)	−1.74	1.67 ± 0.23	2.17	2.11 (2.13)	1.10

^a^ Values in parentheses are the full width at half maximum (FWHM) of the peak.

^b^ Sum of the measured length and snap‐back length of 0.5 nm.

^c^ Obtained from molecular junction structures optimized by DFT. Values in parentheses are obtained from DFT calculations for free molecules.

To illustrate how changing substituents affects the conductance, we next discuss the zero‐bias transmission calculations for the 15Ind derivatives, as shown in Figure 6c. The theoretical conductance values (log(*G*/*G*_0_)) derived from the transmission spectra were calculated to be −1.25 for **15Ind‐b** and −1.74 for **15Ind‐c**, resulting in the order **15Ind‐b** > **15Ind‐a **> **15Ind‐c**. Since charge transport in the 15Ind system is dominated by a LUMO‐mediated channel, the electron‐withdrawing substituents in **15Ind‐b** lower the LUMO energy toward the Fermi level, thereby increasing the conductance. In contrast, the electron‐donating substituents in **15Ind‐c** raise the LUMO energy, shifting it slightly away from the Fermi level and resulting in lower calculated conductance than that of **15Ind‐a**.

Although NEGF–DFT calculations tend to overestimate conductance values because of the overestimated interaction between the molecule and electrodes, the qualitative trend is well reproduced. It should be noted that **15Ind‐a** and **15Ind‐c** exhibit nearly identical conductance experimentally, in contrast to the NEGF‐DFT results. This behavior can be rationalized by considering their experimentally determined FMO energy levels. While the DFT calculations indicate that the LUMO energy level of **15Ind‐c** is slightly higher than that of **15Ind‐a** by 0.07 eV, CV measurements reveal a much smaller LUMO difference of only 0.01 eV. This minimal energy difference is consistent with the very small experimentally observed difference in their conductance values.

These results demonstrate that the conductance of antiaromatic 15Ind‐based molecular bridges can be effectively tuned by substituent modifications, highlighting the feasibility of modulating charge transport through antiaromatic systems.

## 3 Conclusion

In conclusion, this study aimed to directly demonstrate the influence of molecular antiaromaticity on single‐molecule electrical conductance by synthesizing and characterizing length‐matched molecular wires based on 12 π‐electron antiaromatic *s*‐indacene derivatives **15Ind‐a** and **26Ind** and comparing them with the 14 π‐electron aromatic benzodithiophenes **37BDT** and **26BDT**, respectively. Our results demonstrate that antiaromatic *s*‐indacene cores enhance single‐molecule electrical conductance relative to aromatic BDT analogues. This clearly highlights the crucial roles of both antiaromatic character and conjugation pathway in governing charge transport and electric conductance.

Although the horizontally anchored **26Ind** exhibit negligible orbital distribution in the LUMO, its electrical conductance is higher than that of the aromatic **26BDT**. Theoretical calculations provide insight into this observation; the intrinsically small HOMO–LUMO gap arising from the antiaromatic character of **26Ind** brings the HOMO close to the Fermi level, allowing it to contribute partially to the overall conductance and thereby sustain efficient charge transport, effectively surmounting the disadvantages of cross‐conjugation and the weak LUMO coupling. Furthermore, by introducing substituents of different electronic characters into the 15Ind system to yield **15Ind‐b** and **15Ind‐c**, the FMO energies can be effectively modulated. Because charge transport in these systems proceeds mainly through a LUMO‐mediated channel, the lowered LUMO energy of **15Ind‐b** aligns it closer to the Fermi level, resulting in an increased conductance.

Overall, these findings underscore the intricate interplay among core (anti)aromaticity, FMO energetics and spatial distributions, anchor point connectivity, and π‐conjugation pathways in determining single‐molecule electrical conductance. Notably, this work demonstrates, for the first time in a pure hydrocarbon system, highly advantageous effects of antiaromaticity on single‐molecule electrical conductance, while emphasizing the importance of optimizing the coupling of critical orbitals to the electrodes. This study thus provides a valuable framework for the rational design of highly conductive molecular wires through the exploitation of molecular antiaromaticity and orbital engineering.

## Conflicts of Interest

The authors declare no conflicts of interest.

## Supporting information




**Supporting File**: anie73075‐sup‐0001‐SuppMat.pdf.
Supporting information can be found online in the Supporting Information section, which includes details for the synthetic experiments, cyclic voltammetry, and theoretical calculations, and additional data for STM‐BJ experiments. The authors have cited additional references within the Supporting Information [59, 60, 61, 62, 63, 64, 65, 66, 67, 68, 69, 70, 71, 72, 73, 74, 75, 76].

## Data Availability

The data that support the findings of this study are available in the Supporting Information of this article.
