# Supplementary material for: Antiaromaticity‐Driven Enhancement of Single‐Molecule Electrical Conductance by s‐Indacene Molecular Bridges
Source: Angew Chem Int Ed Engl. 2026 Jun 12;65(33):e3299896. doi: 10.1002/anie.3299896 (PMC13452568; doi:10.1002/anie.3299896)
Supplement: Supplementary file 1 [file ANIE-65-e3299896-s001.pdf]

Supporting Information for

Antiaromaticity-Driven Enhancement of Single-Molecule Electrical Conductance by *s*-Indacene Molecular Bridges

Ching-Piao Chu,[#] Yuki Hanamura,[¶] Tatsuhiko Ohto,[ǂ,¶] Seiya Maeda,[¶] Ryo Yamada,[¶] Siao-Lei Yu,[#] Yen-Ju Cheng,[#,§,*] Hirokazu Tada,[¶,*] Yoshito Tobe[#,*]

[#] Department of Applied Chemistry, National Yang Ming Chiao Tung University, 1001 Ta Hsueh Road, Hsinchu 30010, Taiwan

[¶] Graduate School of Engineering Science, The University of Osaka, 1-3 Machikaneyama, Toyonaka 560-8531, Japan

[ǂ] Graduate School of Engineering, Nagoya University, Furo-cho, Chikusa-ku, Nagoya 464-8603, Japan

[§] Center for Emergent Functional Matter Science, National Yang Ming Chiao Tung University, 1001 Ta Hsueh Road, Hsinchu 30010, Taiwan

Contents:

1. General Experimental Procedures
2. Synthesis of **15Ind-a**, **15Ind-b, 15Ind-c, 26Ind**, **37BDT**, and **26BDT**
3. Previous Comparative Studies on Antiaromatic Systems
4. Cyclic Voltammetry of Bridge Molecules
5. Optical Properties of Bridge Molecules
6. Theoretical Calculations
   6.1. General
   6.1.1. Theoretical and Experimental Electronic Transitions
   6.1.2. Diradical Characters
   6.1.3. NICS Calculations
   6.2. NEGF-DFT Calculations
7. STM-BJ Experiments
   7.1. Hardware Details
   7.2. Experimental Details
   7.3. Extraction of Conductance Traces
   7.4. Conductance Analysis
   7.5. Plateau Length Analysis
8. Results of Transport Calculations
9. References

10. ^{1}H and ^{13}C NMR Spectra of New Compounds
11. Cartesian Coordinates for Optimized Geometries of **15Ind-a**, **15Ind-b, 15Ind-c, 26Ind**, **37BDT**, and **26BDT**

1. General Experimental Procedures

Melting points were measured with a MANDARIN SCIENTIFIC MP-2D apparatus. ^{1}H and ^{13}C NMR spectra were measured with a Aligent 400-MR DD2, Bruker AVANCEIII-400, or a Bruker AVANCEIII-500 spectrometer at 25 °C unless otherwise stated in chloroform-*d*, dichloromethane-d_2, tetrachloroethane-d_2 (at 60 °C or 100 °C). For the ^{1}H NMR measurements, the residual solvent signals were used as references for $CDCl_3$ (7.26 ppm) and $C_2D_2Cl_4$ (6.00 ppm). For the ^{13}C NMR measurements, the carbon signals were used as references at 77.16 ppm for $CDCl_3$ and 73.78 ppm for $C_2D_2Cl_4$. IR and UV-vis-NIR spectra were recorded with Perkin Elmer Spectrum One FT-IR and Shimadzu UV-3600 spectrophotometers, respectively. Mass spectrometry was conducted with a JEOL JMS-T200GC AccuTOF GCx mass spectrometer in FD (Field Desorption) mode. Cyclic voltammetric measurements were performed with a CV apparatus CHI611D (CH Instruments) using a glassy carbon working electrode, a Pt counter electrode, and an Ag/$AgNO_3$ reference electrode at room temperature using 1 – 5 mM of analyte in dichloromethane (DCM) containing 0.1 M Bu_4NPF_6 as the supporting electrolyte. Column chromatography and TLC were performed with UNI-WARD SiliaFlash Irregular Silica Gel G60, 60-200 µm, 60 Å (R10040B) (70–230 mesh ASTM) and Merck silica gel 60 F254, respectively. For chromatography on aluminum oxide gel, Macherey-Nagel Aluminum Oxide 90 Neutral was used. 1-Ethynyl-3,5-dimethylbenzene,[59] 5,5'-((2,5-dibromo-1,4-phenylene)bis(ethyne-2,1-diyl))bis(1,3-dimethylbenzene),[36] *N*,*N*,3,5-tetramethylbenzamide,[60] *N*,*N*-dimethyl-4-(trifluoromethyl)benzamide,[61] 4-methoxy-*N*,*N*-dimethylbenzamide,[61] 2,6-dimethyl-4-(methylthio)benzaldehyde,[62] and (3,7-diiodobenzo[1,2-b:4,5-b']dithiophene-2,6-diyl)bis(trimethylsilane)[63] (**10**) were prepared according to the literature methods. Other reagents were used as supplied. Tetrahydrofuran (THF) was dried using a solvent purification system SD-300 (Asia Wong Enterprise).

2. Synthesis of 15Ind-a, 15Ind-b, 15Ind-c, 26Ind, 37BDT, and 26BDT

Synthesis of 15Ind-a, 15Ind-b, and 15Ind-c

The synthetic route of **15Ind** derivatives is shown in Scheme S1.

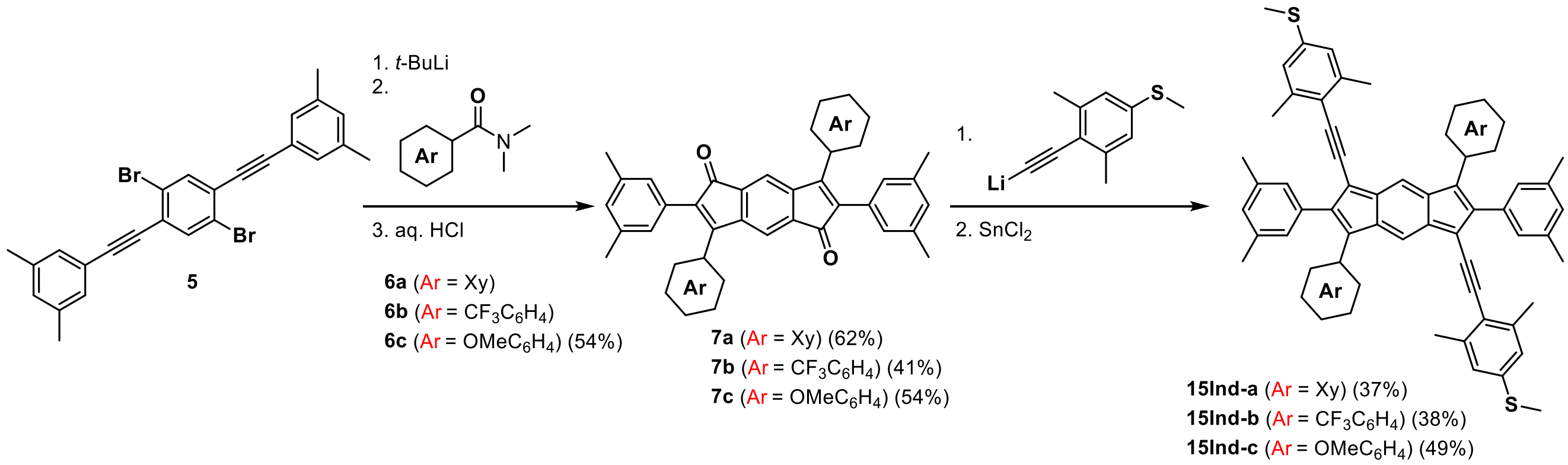

Scheme S1. Synthetic scheme for **15Ind-a, 15Ind-b** and **15Ind-c** with respective yields in parentheses.

Synthesis of diketones 7a-7c

7a:

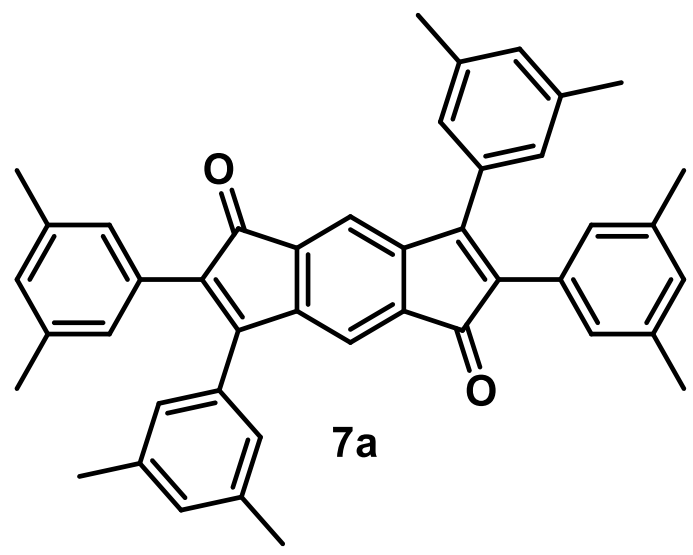

Dibromide **5**[36] (1.00 g, 2.03 mmol) was dissolved in dry THF (37 mL) in an oven-dried 100 mL double neck round bottom flask. After cooling to −90 °C, *t*-BuLi (1.9 M in hexane, 5.30 mL, 10.2 mmol) was added to the reaction mixture via a syringe under a nitrogen atmosphere. After stirring for 1 h, *N*,*N*,3,5-tetramethylbenzamide **6a**[60] (2.16 g, 12.2 mmol) dissolved in THF (10 mL) was added dropwise over a 15 min period at the same temperature. The reaction mixture was stirred at –90 °C for 1 h then warmed to room temperature. The mixture was heated to reflux for 2 h. After cooling to room temperature, the reaction mixture was quenched by addition of 5M HCl solution (20 mL) and stirred at 80 °C for 12 h, during which period blue precipitates were formed. The solid was collected by suction filtration and washed with methanol and cold acetone, and dried in an oven to give **7a** (755 mg, 62%) as a blue solid. The ^{1}H NMR spectrum agreed with the literature.[36]

7b:

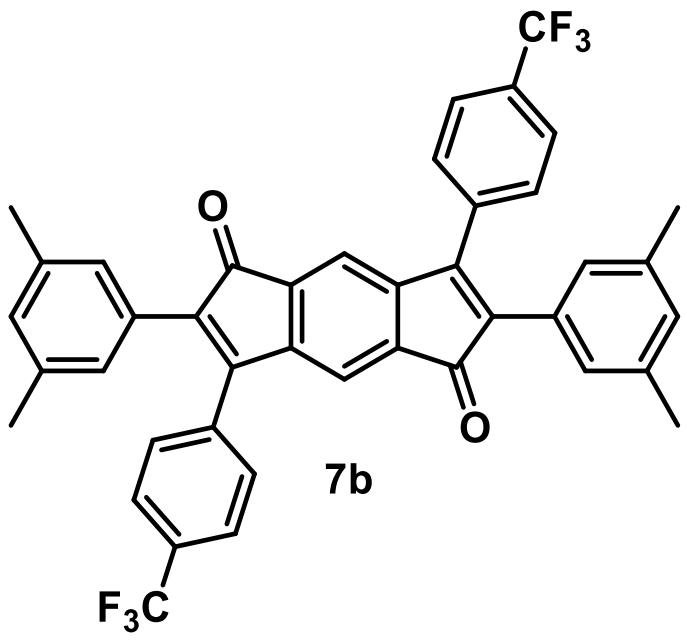

7b

Using dibromide **5**[36] (1.00 g, 2.03 mmol), *t*-BuLi (1.9 M in hexane, 4.28 mL, 8.13 mmol), and *N*,*N*-dimethyl-4-(trifluoromethyl)benzamide **6b**[61] (971 mg, 4.47 mmol), then reflux for 3h, **7b** (541 mg, 41%) was obtained after washing with methanol and cold acetone as a blue solid. mp 321.3–322.1 °C; ^{1}H NMR (400 MHz, $CDCl_3$) δ 7.72 (d, *J* = 8.0 Hz, 4H), 7.52 (d, *J* = 8.0 Hz, 4H), 7.34 (s, 2H), 6.93 (s, 2H), 6.81 (s, 4H), 2.22 (s, 12H); ^{13}C{^{1}H}{^{19}F} NMR (100 MHz, $CDCl_3$) δ 195.5, 152.6, 147.1, 138.0, 136.2, 134.6, 133.7, 131.6, 130.5, 129.5, 129.1, 127.7, 126.1, 123.9, 115.7, 21.4; FT-IR $\tilde{\nu}$ 2917 (w), 1705 (m), 1590 (w), 1439 (w), 1320 (m), 1163 (m), 1119 (m), 1067 (m), 1017 (m), 844 (m), 686 (m), 671 (m) cm^{-1}. HRMS (FD) *m/z* calcd for $C_{42}H_{28}F_6O_2$ [M^+] 678.1988, found 678.1978.

3c:

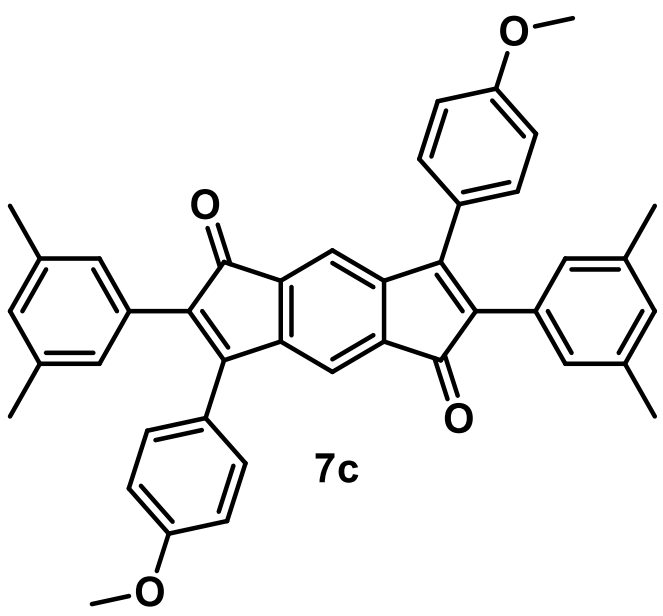

7c

Using dibromide **5**[36] (1.00 g, 2.03 mmol), *t*-BuLi (1.9 M in hexane, 4.28 mL, 8.13 mmol), and 4-methoxy-*N*,*N*-dimethylbenzamide **6c**[61] (801 mg, 4.47 mmol), then reflux for 2h, **7c** (655 mg, 54%) was obtained after washing with methanol and cold acetone as a blue solid. mp 284.2–284.8 °C; ^{1}H NMR (400 MHz, $CDCl_3$) δ 7.45 (s, 2H), 7.37 (d, *J* = 8.8 Hz, 4H), 6.95 (d, *J* = 8.8 Hz, 4H), 6.90 (s, 2H), 6.87 (s, 4H), 3.88 (s, 6H), 2.23 (s, 12H); ^{13}C{^{1}H} NMR (100 MHz, $CDCl_3$) δ 196.0, 160.7, 154.3, 147.1, 137.6, 134.9, 131.4, 130.5, 130.3, 129.6, 127.7, 124.5, 115.5, 114.3, 55.4, 21.3; FT-IR $\tilde{\nu}$ 2921 (m), 1702 (m), 1601 (m), 1507 (m), 1437 (w), 1334 (w), 1292 (w), 1174 (m), 1132 (m), 1026 (m), 900 (w), 852 (m), 804 (m), 765 (m), 689 (m) cm^{-1}. HRMS (FD) *m/z* calcd for $C_{42}H_{34}O_4$ [M^+] 602.2452, found 602.2457.

Synthesis of 15Ind derivatives

15Ind-a:

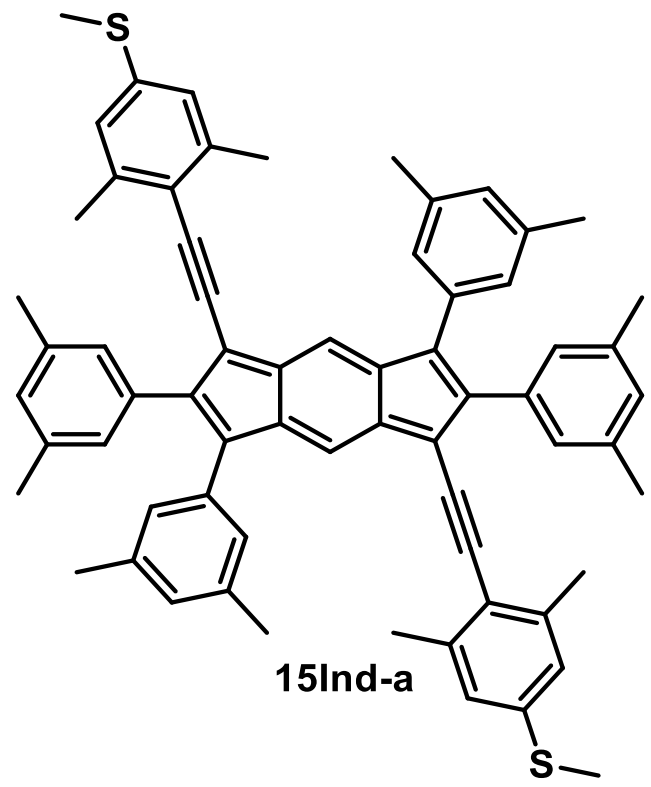

15Ind-a

(4-Ethynyl-3,5-dimethylphenyl)(methyl)sulfane **15**[62] (147 mg, 0.835 mmol) was dissolved in dry THF (10 mL) in an oven-dried 50 mL double neck round bottom flask and cooled to 0 °C. *n*-BuLi (2.5 M in hexane, 0.33 mL, 0.83 mmol) was added to this solution slowly during a 3 min period and the solution was kept at the same temperature for 1 h. A solution of dione **7a**[36] (100 mg, 0.167 mmol) dissolved in dry THF (5 mL) was added to the above acetylide solution via a syringe during a 10 min period, then the solution was maintained at −90 °C for 1 h. After warming to room temperature, the solution was stirred for 30 min before it was quenched by addition of saturated NH_4Cl solution. The mixture was extracted with EtOAc, and the extract was washed with water and brine, and the organic layer was dried over $MgSO_4$. The solvent was removed under vacuum to give the crude diol, which was carried on without further purification.

In a two-neck round-bottom flask, the crude diol was added, and the flask was charged with nitrogen. The diol was dissolved in dry toluene (17 mL) and $SnCl_2$ (127 mg, 0.668 mmol) was added under a nitrogen flow. The mixture was stirred at 0 °C for 2 h and 40 min. The reaction mixture was directly subjected to alumina column (elusion with chloroform) to remove inorganic materials. The solvent was evaporated under reduced pressure to give a dark green solid, which was recrystallized from chloroform/acetone and washed successively with MeOH, acetone, and pentane to give **15Ind-a** (64 mg, 42%) as a dark green solid. ^{1}H NMR (400 MHz, $CDCl_3$) δ 6.90 (s, 2H), 6.85 (s, 4H), 6.81 (s, 6H), 6.73 (s, 4H), 6.67 (s, 2H), 2.45 (s, 6H), 2.23 (s, 12H), 2.21 (s, 12H), 2.19 (s, 12H); ^{13}C{^{1}H} NMR (100 MHz, $CDCl_3$) δ 152.6, 143.1, 141.1, 139.7, 138.8, 137.2, 136.8, 135.4, 134.3, 134.1, 133.4, 130.0, 128.7, 127.5, 127.4, 127.1, 124.1, 120.2, 104.1, 97.2, 21.5, 21.4, 21.4, 15.3; FT-IR $\tilde{\nu}$ 2902 (w), 2144 (w), 1579 (w), 1513 (m), 1371 (m), 1254 (m), 1091 (m), 852 (m), 842 (m), 705 (m) cm^{-1}. HRMS (FD) *m/z* calcd for $C_{66}H_{60}S_2$ [M^+] 916.4131, found 916.4148.

15Ind-b:

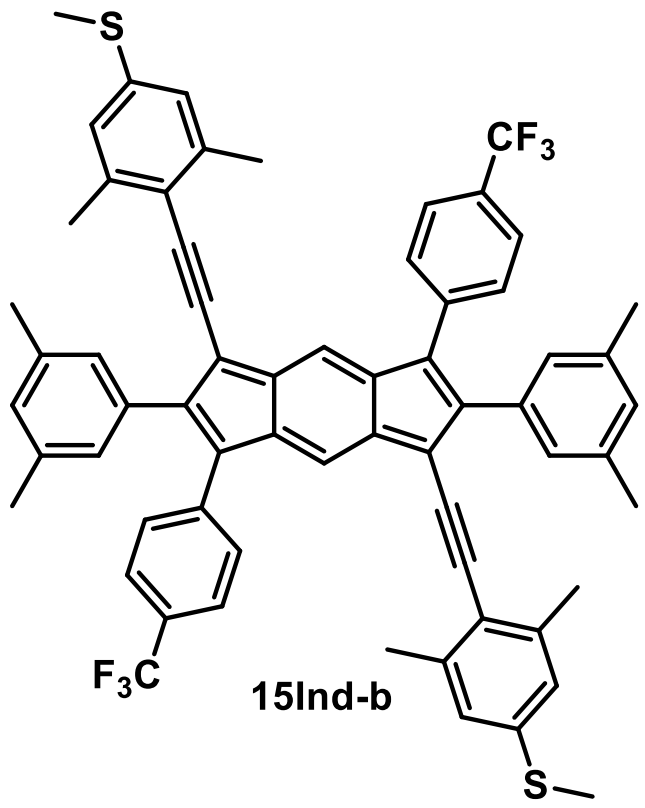

15Ind-b

Using compound **15**[62] (260 mg, 1.47 mmol), *n*-BuLi (2.5 M in hexane, 0.56 mL, 1.40 mmol), dione **7b** (100 mg, 0.147 mmol) and $SnCl_2$ (112 mg, 0.589 mmol), by stirring at 0 °C for 3 h, **15Ind-b** (100 mg, 68%) was obtained as blue solid after washing with MeOH, acetone, and pentane. ^{1}H NMR (400 MHz, $CDCl_3$) δ 7.50 (d, J = 8.0 Hz, 4H), 7.25 (d, overlapping with $CDCl_3$, J = 8.0 Hz, 4H), 6.87 (overlapping s + s, 6H), 6.80 (s, 4H), 6.62 (s, 2H), 2.47 (s, 6H), 2.22 (s, 12H), 2.19 (s, 12H); ^{13}C{^{1}H}{^{19}F} NMR (100 MHz, $CDCl_3$) δ 148.6, 142.8, 141.3, 140.9, 138.8, 137.9, 137.4, 136.7, 135.8, 133.5, 129.8, 129.7, 129.3, 127.2, 126.9, 124.9, 124.3, 124.2, 119.7, 107.2, 97.5, 21.4, 21.3, 15.2; FT-IR $\tilde{\nu}$ 2914 (w), 2147 (m), 1583 (m), 1512 (m), 1372 (m), 1317 (m), 1236 (m), 1163 (m), 1120 (m), 1064 (m), 1061 (m), 1013 (m), 843 (m) cm^{-1}. HRMS (FD) *m/z* calcd for $C_{64}H_{50}F_6S_2$ [M$^+$] 966.3253, found 966.3255.

15Ind-c:

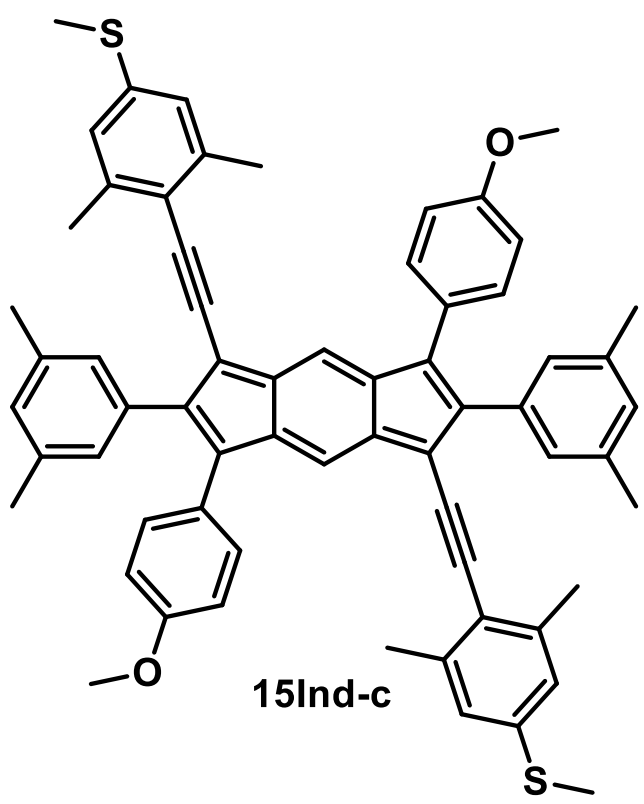

15Ind-c

Using compound **15**[62] (292 mg, 1.66 mmol), *n*-BuLi (2.5 M in hexane, 0.63 mL, 1.58 mmol), dione **7c** (100 mg, 0.166 mmol) and $SnCl_2$ (126 mg, 0.664 mmol), by stirring at 0 °C for 2 h, **15Ind-c** (83 mg, 54%) was obtained as blue solid after washing with MeOH, acetone, and pentane. ^{1}H NMR (400 MHz, $CDCl_3$) δ 7.11 (d, J = 8.8 Hz, 4H), 6.87 (s, 4H), 6.84 (s, 6H), 6.81 (s, 2H), 6.79 (d, J = 8.8

Hz, 4H), 3.82 (s, 6H), 2.46 (s, 6H), 2.29 (s, 12H), 2.20 (s, 12H); $^{13}C\{^{1}H\}$ NMR (100 MHz, $CDCl_3$) δ 159.9, 151.8, 142.4, 141.0, 139.6, 138.9, 137.0, 134.6, 134.5, 132.8, 131.2, 128.8, 127.2, 127.1, 127.1, 124.2, 120.3, 113.5, 103.6, 97.0, 55.5, 21.4, 15.3; FT-IR $\tilde{\nu}$ 2914 (w), 2148 (m), 1600 (m), 1583 (m), 1523 (m), 1503 (m), 1378 (m), 1246 (m), 1174 (m), 1102 (m), 1030 (m) cm^{-1}. HRMS (FD) *m/z* calcd for $C_{64}H_{56}O_2S_2$ [M^+] 920.3716, found 920.3707.

Synthesis of 26Ind

The synthetic route of **26Ind** is shown in Scheme S2.

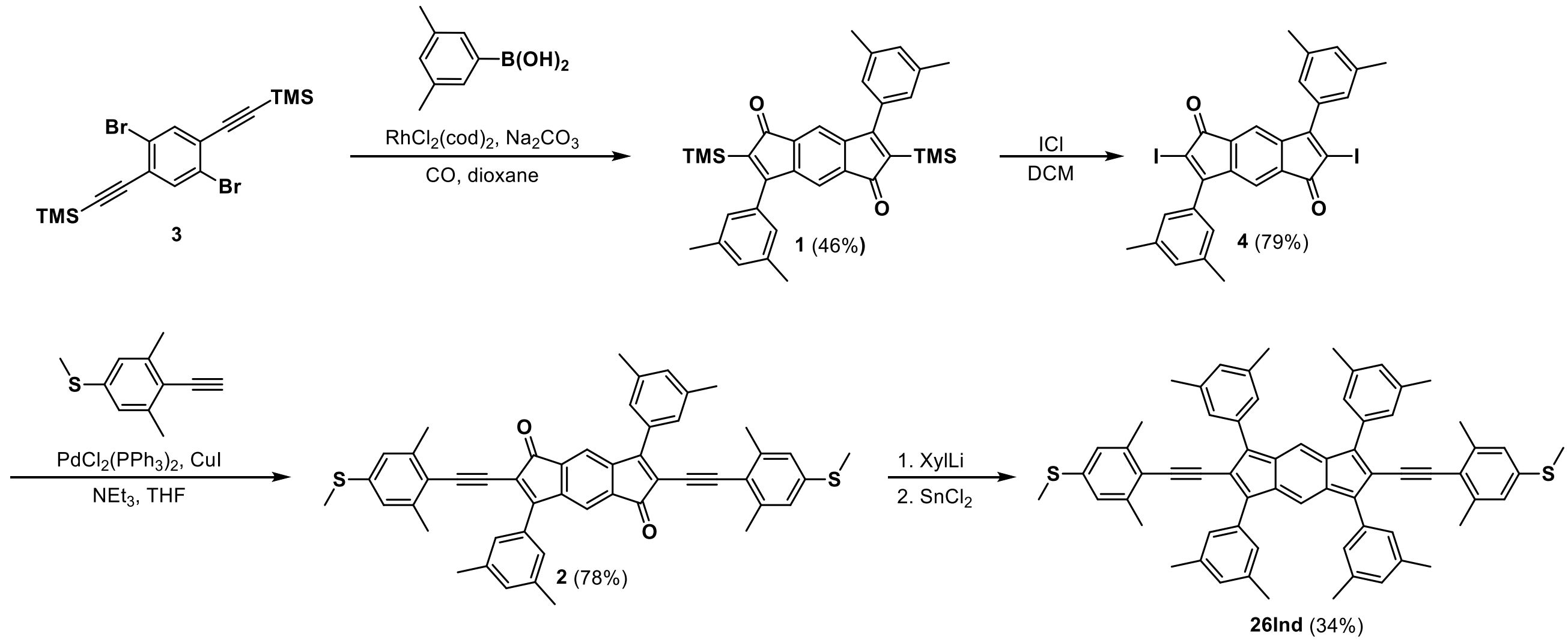

Scheme S2. Synthetic scheme for **26Ind** with respective yields in parentheses.

Synthesis of diketone 1:

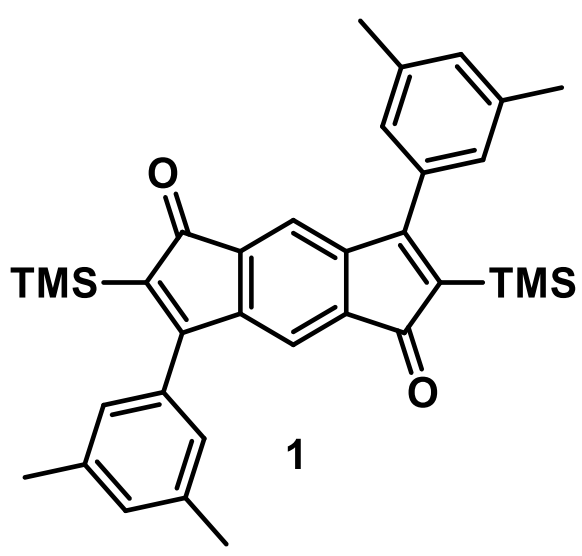

A 10-mL two-necked flask was charged with dibromide **3**[64] (100 mg, 0.233 mmol), sodium carbonate (99 mg, 0.9 mmol), (3,5-dimethylphenyl)boronic acid (210 mg, 1.40 mmol), and $[RhCl(cod)]_2$ (17 mg, 0.035 mmol). The system was carefully evacuated and filled with carbon monoxide (1 atm). The reagents were dissolved in dry dioxane (2.3 mL) that had been deaerated by bubbling carbon monoxide for 30 min, and a balloon filled with carbon monoxide was attached to the flask. The flask was then immersed in an oil bath at 100 ˚C for 40 h. The reaction mixture was filtered through a pad of Celite. After removal the solvent under reduced pressure, the residue was purified by

column chromatography on silica gel (hexane/DCM, v/v 7/3) to give **1** (57 mg, 46%) as a red solid. mp 283.7-284.5 ˚C; ^{1}H NMR (400 MHz, $CDCl_3$) δ 7.09 (s, 2H), 7.08 (s, 2H), 6.94 (s, 4H), 2.38 (s, 12H), 0.04 (s, 18H); ^{13}C{^{1}H} NMR (100 MHz, $CDCl_3$) δ 201.2, 170.8, 149.4, 138.2, 136.5, 134.4, 131.2, 125.4, 115.0, 21.5, 0.0; FT-IR $\tilde{\nu}$ 2954 (w), 1697 (m), 1545 (w), 1427 (w), 1320 (w), 1245 (m), 1229 (m), 1128 (m), 871 (m), 821 (m) cm^{-1}. HRMS (FD) *m/z* calcd for $C_{34}H_{38}O_2Si_2$ [M^+] 534.2405, found 534.2410.

Synthesis of diketone 4:

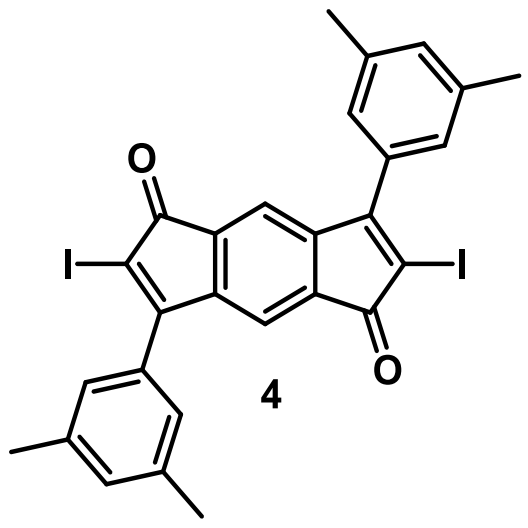

Dione **1** (150 mg, 0.28 mmol) was dissolved in DCM (4 mL), then the solution was cooled to 0 ˚C and kept for 10 min. Iodine chloride (100 mg, 0.62 mmol) dissolved in DCM (1 mL) was slowly added to the solution. The reaction mixture was stirred at room temperature for overnight, then quenched with aqueous $NaHCO_3$. The mixture was diluted with water and the aqueous phase was extracted with DCM (3 × 20 mL). After removing the solvent from the extract under reduced pressure, the solid was collected by suction filtration, washed with acetone, and dried in an oven to yield **4** (160 mg, 89%) as a green solid. ^{1}H NMR (400 MHz, $CDCl_3$) δ 7.34 (s, 2H), 7.19 (overlapping s + s, 6H), 2.44 (s, 12H); ^{13}C{^{1}H} NMR (100 MHz, $CDCl_3$) δ 190.8, 163.4, 147.7, 138.9, 134.8, 132.7, 132.1, 125.6, 116.3, 94.7, 21.6; FT-IR ṽ 2916 (w), 1707 (m), 1597 (m), 1547 (m), 1429 (m), 1302 (m), 1222 (m), 1132 (m), 1078 (m), 1020 (m), 905 (m), 857 (m), 825 (m), 705 (m) cm^{-1}. HRMS (FD) *m/z* calcd for $C_{28}H_{20}I_2O_2$ [M^+] 641.9547, found 641.9547.

Synthesis of diketone 2:

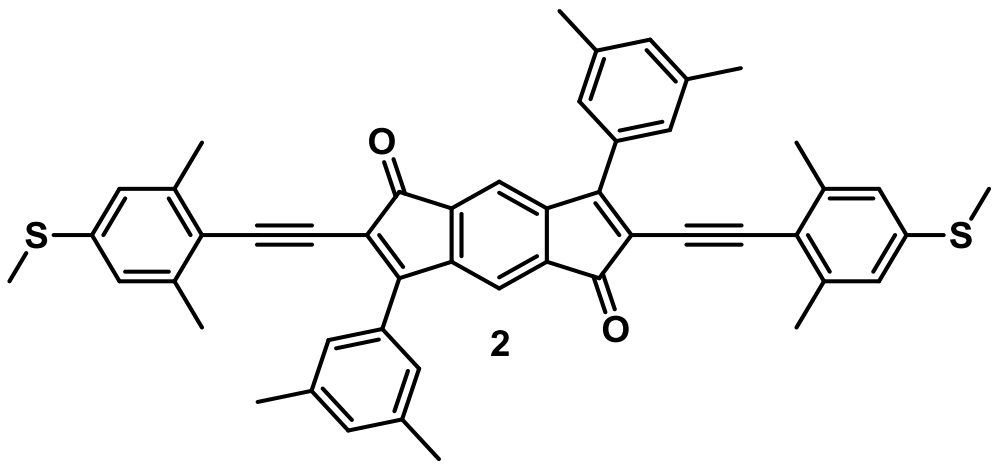

In a 50 mL two-neck round-bottom flask, dione **4** (80 mg, 0.13 mmol) and sulfane **15**[62] (55 mg, 0.31 mmol) were dissolved in 1:1 (v/v) triethylamine (6 mL) / THF (6 mL). The reaction mixture was

deaerated by nitrogen bubbling and $PdCl_2(PPh_3)_2$ (8.7 mg, 0.012 mmol, 10 mol%) and CuI (2.4 mg, 0.012 mmol, 10 mol%) were added. The mixture was stirred at 70 °C for 12 h. After removal of the solvent under reduced pressure, the solid was collected by suction filtration, washed successively with methanol, acetone, and ethyl acetate and pentane and dried in an oven to yield **2** (72 mg, 78%) as a green solid. ^{1}H NMR (500 MHz, $C_2D_2Cl_4$, 100 °C) δ 7.61 (s, 2H), 7.54 (s, 4H), 7.24 (s, 2H), 7.00 (s, 4H), 2.50 (overlapping s + s, 30H); ^{13}C{^{1}H} NMR (125 MHz, $C_2D_2Cl_4$, 100 °C) δ 192.2, 157.5, 147.0, 141.2, 139.6, 138.4, 135.1, 132.5, 131.7, 125.7, 124.6, 119.5, 117.9, 116.3, 99.0, 90.4, 21.1, 20.8, 15.3; FT-IR $\tilde{\nu}$ 2921 (w), 2177 (m), 1717 (m), 1589 (m), 1561 (m), 1443 (m), 1145 (m), 1090 (m), 882 (m), 842 (m) cm^{-1}. HRMS (FD) *m/z* calcd for $C_{50}H_{42}O_2S_2$ [M$^+$] 738.2621, found 738.2608.

Synthesis of 26Ind:

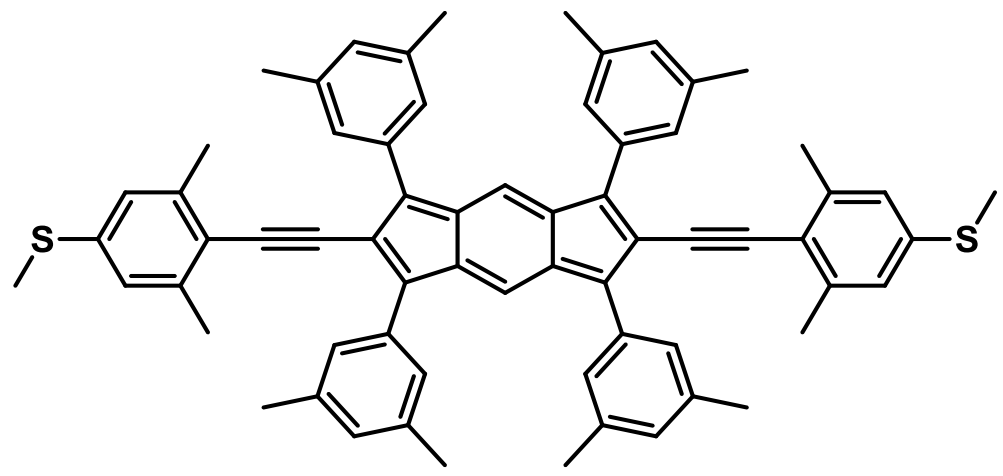

26Ind

1-Bromo-3,5-dimethylbenzene (188 mg, 1.02 mmol) was dissolved in dry THF (3 mL) in an oven-dried 25 mL double neck round bottom flask and cooled to –90 °C. *n*-BuLi (2.5 M in hexane, 0.39 mL, 0.98 mmol) was added to this solution slowly during a 3 min period and the solution was kept at the same temperature for 1 h. A solution of dione **2** (50 mg, 0.068 mmol) dissolved in dry THF (4 mL) was added to the above aryllithium solution via a syringe during a 10 min period The solution was maintained at −90 °C for 1 h before warming up to room temperature during 1 h. After warming to room temperature, the solution was stirred for 1 h before it was quenched by addition of saturated NH_4Cl solution. The mixture was extracted with EtOAc, and the extract was washed with water and brine, and the organic layer was dried over $MgSO_4$. After removing the solvent under reduced pressure, the residue was passed through a column on alumina (hexane/ethyl acetate, v/v 4/6) to remove polar impurities, affording a crude product containing the diol.

In a two-neck round-bottom flask was added the crude diol and the flask was charged with nitrogen. The crude diol was dissolved in dry toluene (7 mL) and $SnCl_2$ (58 mg, 0.30 mmol) was added under a nitrogen flow. The mixture was stirred at 80 °C for 1 h. The reaction mixture was directly subjected on filter paper (dissolved with DCM) to remove inorganic materials. The solvent was evaporated under reduced pressure to give a dark green solid, which was recrystallized from chloroform/methanol and washed successively with methanol, acetonitrile, and pentane to give **26Ind**

(21 mg, 34%) as a dark green solid. ^{1}H NMR (400 MHz, $CDCl_3$) δ 7.24 (s, 8H), 6.98 (s, 4H), 6.80 (s, 4H), 6.63 (s, 2H), 2.42 (s, 6H), 2.32 (s, 24H), 2.10 (s, 12H); ^{13}C{^{1}H}NMR (125 MHz, $C_2D_2Cl_4$, 100 °C) δ 155.2, 140.4, 137.4, 135.3, 133.6, 132.0, 130.6, 126.9, 124.3, 121.2, 120.7, 120.4, 93.7, 92.2, 21.1, 20.8, 15.4; FT-IR $\tilde{\nu}$ 2913 (w), 2182 (w), 1596 (w), 1530 (m), 1432 (m), 1392 (m), 1341 (w), 1245 (m), 850 (m), 722 (w), 691 (m) cm^{-1}. HRMS (FD) *m/z* calcd for $C_{66}H_{60}S_2$ [M$^+$] 916.4131, found 916.4128.

Synthesis of 37BDT

The synthetic route of **37BDT** is shown in Scheme S3.

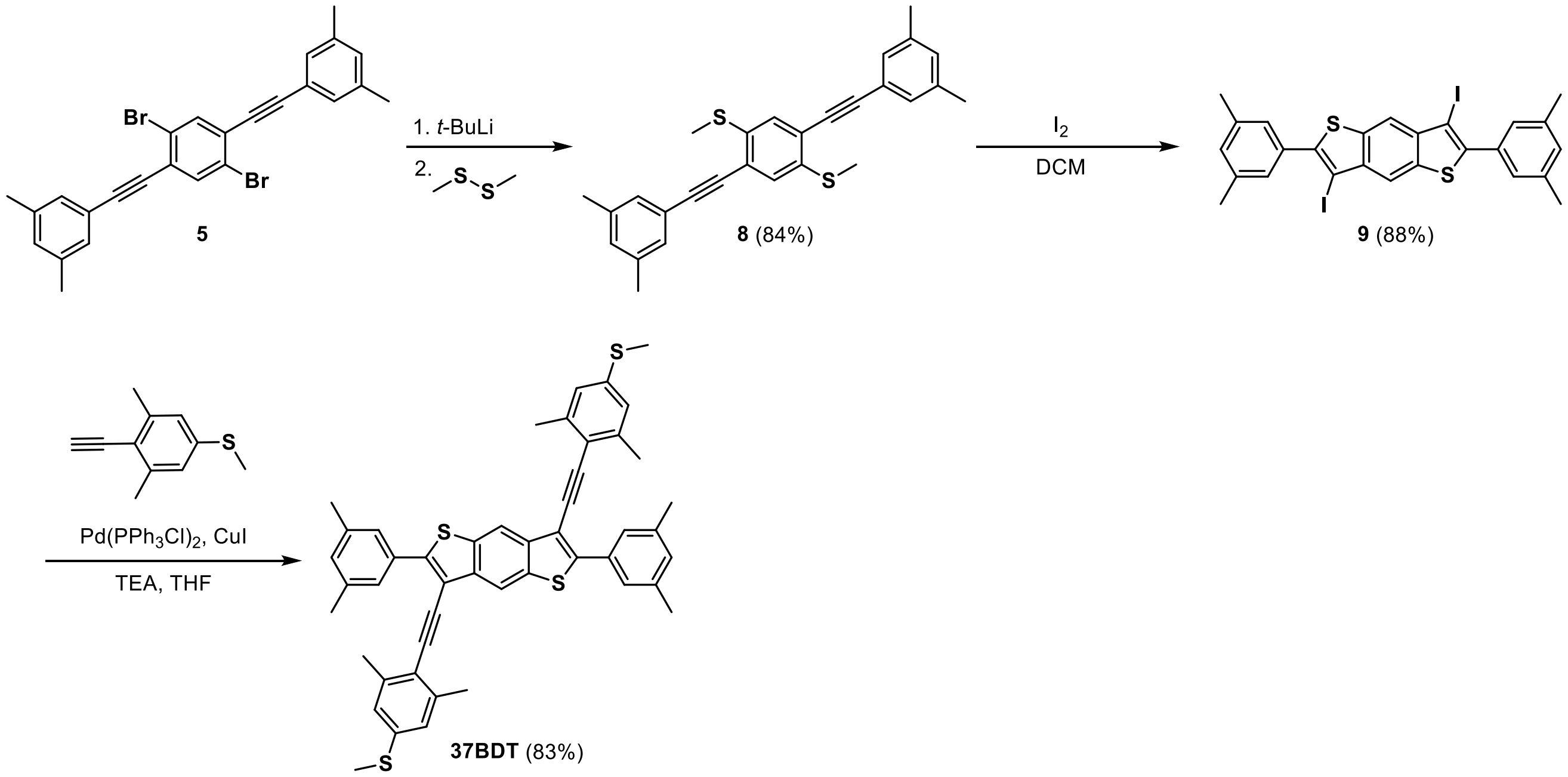

Scheme S3. Synthetic scheme for **37BDT** with respective yields in parentheses.

Synthesis of diacetylene 8:

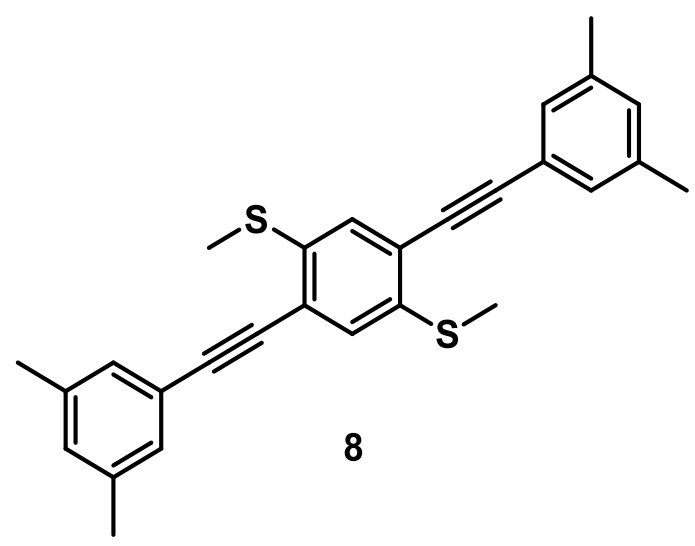

8

Dibromide **5**[36] (1.00 g, 2.03 mmol) was dissolved in dry THF (20 mL) in an oven-dried 50 mL double neck round bottom flask. After cooling to −90 °C, *t*-BuLi (1.9 M in hexane, 4.30 mL, 8.13 mmol) was added to the reaction mixture via a syringe under a nitrogen atmosphere. After stirring for 1 h, 1,2-dimethyldisulfane (421 mg, 4.47 mmol) was added dropwise over a 5 min period at the same temperature. The reaction mixture was stirred at –90 °C for 1 h then warmed to room temperature.

After warming to room temperature, the reaction mixture quenched by addition of saturated NH_4Cl solution. The mixture was extracted with EtOAc, and the extract was washed with water and brine, and the organic layer was dried over $MgSO_4$. After remove the solvent under reduced pressure, the residue was purified by column chromatography on silica gel (hexane/DCM, v/v 5/1) to give **8** (726 mg, 84%) as a yellow solid. mp 215.9–216.8 °C; ^{1}H NMR (400 MHz, $CDCl_3$) δ 7.27 (s, 2H), 7.22 (s, 4H), 7.00 (s, 2H), 2.53 (s, 6H), 2.32 (s, 12H); ^{13}C{^{1}H} NMR (100 MHz, $CDCl_3$) δ 138.1, 137.7, 130.8, 129.5, 128.1, 122.6, 122.3, 98.0, 86.2, 21.3, 15.6; FT-IR $\tilde{\nu}$ 2919 (w), 1590 (w), 1423 (w), 1362 (w), 1259 (w), 1099 (w), 1038 (w), 861 (w), 840 (m), 681 (m) cm^{-1}. HRMS (FD) *m/z* calcd for $C_{28}H_{26}S_2$ [M$^+$] 426.1470, found 426.1476.

Synthesis of diiodo-BDT 9:

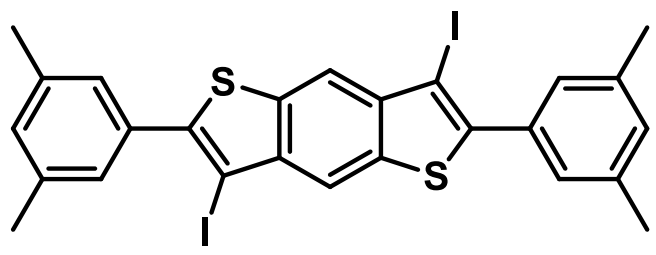

9

Compound **8** (0.50 g, 1.17 mmol) and iodine (0.74 g, 2.93 mmol) were placed in a 25 mL round-bottom flask and dissolved with DCM (11 mL). After stirring the solution at ambient temperature for 3 h, then quenched with aqueous NaOH. The mixture was diluted with water and the aqueous phase was extracted with DCM (3 × 20 mL). The combined organic layer was dried over $MgSO_4$, and the solvent was removed under reduced pressure. The crude product was washed with methanol followed by pentane to give **9** as a light brown solid (0.63 g, 88%). mp 286.4–287.2 °C; ^{1}H NMR (400 MHz, $CDCl_3$) δ 8.25 (s, 2H), 7.35 (s, 4H), 7.11 (s, 2H), 2.43 (s, 12H); ^{13}C{^{1}H} NMR (100 MHz, $CDCl_3$) δ 144.0, 140.8, 138.3, 137.1, 134.5, 131.0, 127.9, 119.5, 21.5; FT-IR $\tilde{\nu}$ 2909 (w), 1730 (w), 1600 (w), 1439 (w), 1371 (w), 1226 (w), 1037 (w), 908 (w), 858 (m), 846 (m), 798 (m), 728 (w), 690 (m) cm^{-1}. HRMS (FD) *m/z* calcd for $C_{26}H_{20}I_2S_2$ [M$^+$] 649.9090, found 649.9089.

Synthesis of 37BDT:

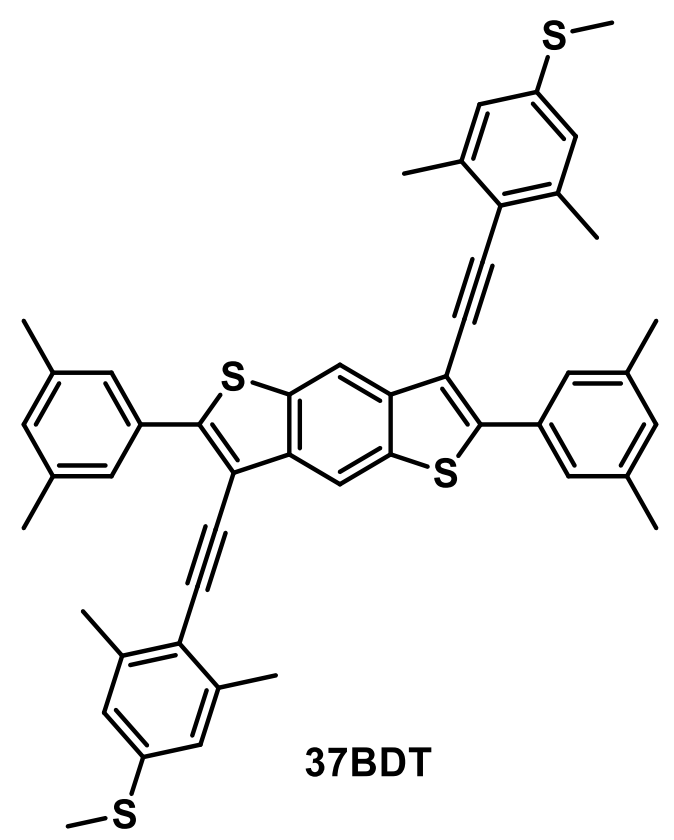

37BDT

In a 50 mL two-neck round-bottom flask, compound **9** (100 mg, 0.154 mmol), sulfane **15**[62] (60 mg, 0.34 mmol) were dissolved in 1:1 (v/v) triethylamine (8 mL) / THF (8 mL). The reaction mixture was deaerated by nitrogen bubbling and $PdCl_2(PPh_3)_2$ (5.4 mg, 0.0077 mmol, 5 mol%) and CuI (1.5 mg, 0.0077 mmol, 5 mol%) were added. The mixture was stirred at 70 °C for 12 h. After removal of the solvent under reduced pressure, the solid was collected by suction filtration, washed successively with methanol, acetone, and pentane and dried in an oven to yield **37BDT** (95 mg, 83%) as a light green solid. mp 287.2–288.4 °C; ^{1}H NMR (400 MHz, $CDCl_3$) δ 8.44 (s, 2H), 7.68 (s, 4H), 7.06 (s, 2H), 6.99 (s, 4H), 2.58 (s, 12H), 2.51 (s, 6H), 2.39 (s, 12H); ^{13}C{^{1}H} NMR (125 MHz, $C_2D_2Cl_4$, 100°C) δ 146.9, 140.6, 139.5, 138.7, 138.1, 135.5, 133.7, 130.7, 126.4, 124.9, 119.9, 116.0, 113.0, 92.5, 92.0, 21.3, 21.0, 15.5; FT-IR $\tilde{\nu}$ 2914 (w), 1586 (w), 1467 (w), 1429 (w), 1256 (w), 1179 (w), 1107 (w), 841 (m), 723 (w), 690 (m) cm^{-1}. HRMS (FD) *m/z* calcd for $C_{48}H_{42}S_4$ [M^+] 746.2164, found 746.2162.

Synthesis of 26BDT

The synthetic route of **26BDT** is shown in Scheme S4.

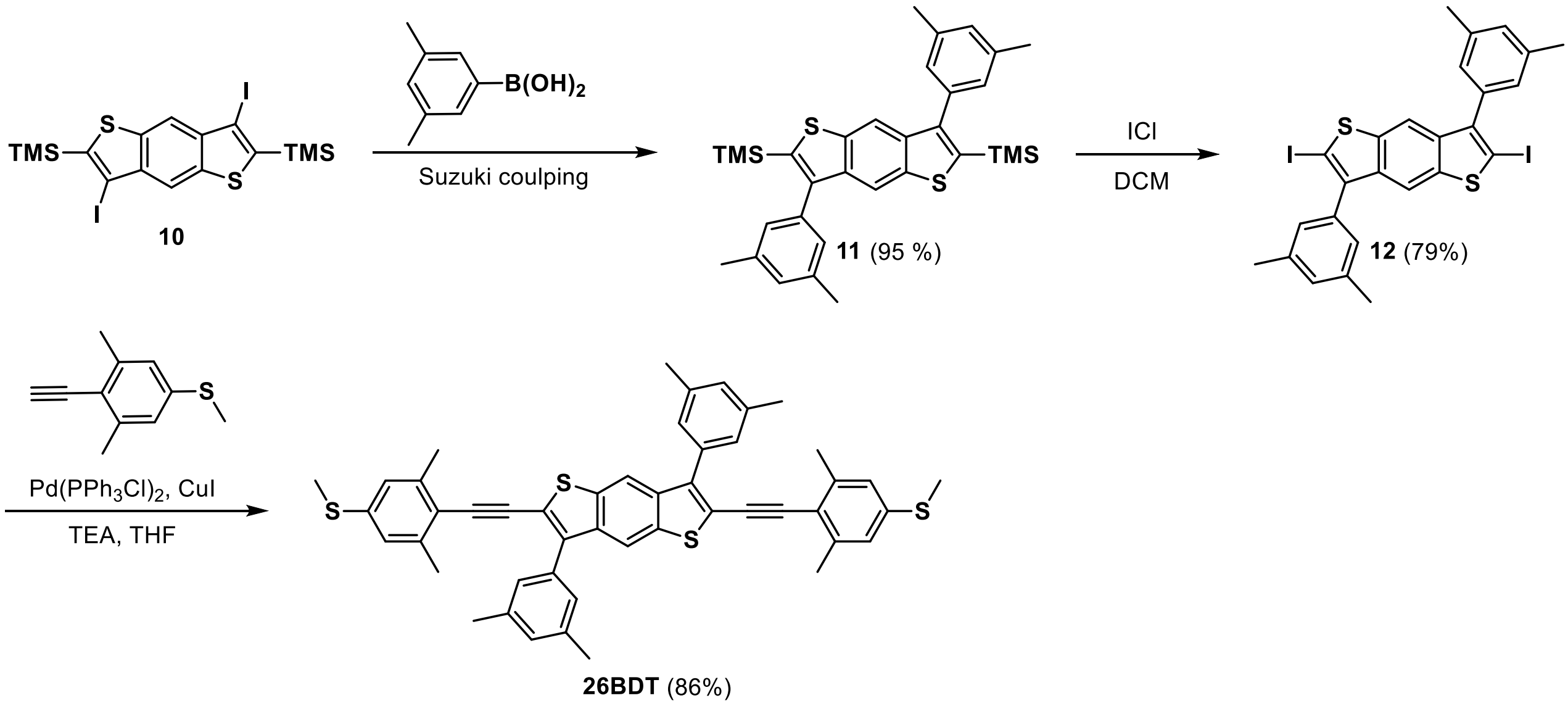

Scheme S4. Synthetic scheme for **26BDT** with respective yields in parentheses

Synthesis of bisTMS-BDT 11:

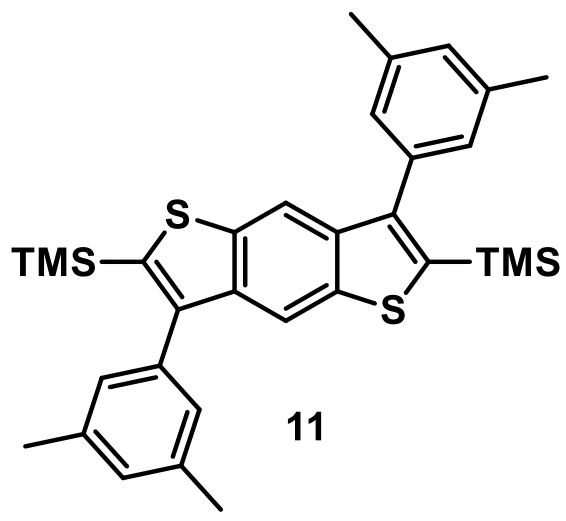

In a 10 mL two-neck round-bottom flask, compound **10**[63] (0.2 g, 0.34 mmol), (3,5-dimethylphenyl)boronic acid (0.26 g, 1.7 mmol), K_2CO_3 (0.24 g, 1.7 mmol) and $Pd(PPh_3)_4$ (39 mg, 0.034 mmol, 10 mol%) were dissolved in 9:1 (v/v) toluene (4.5 mL)/H_2O (0.5 mL). The mixture was stirred at 90 °C for 16 h. The reaction mixture was filtered through a pad of Celite. After removing the solvent under reduced pressure, the residue was purified by column chromatography on silica gel (hexane) to give **11** (0.18 g, 95%) as a white solid. mp 294.9–296.0 °C; ^{1}H NMR (400 MHz, $CDCl_3$) δ 7.93 (s, 2H), 7.09 (s, 2H), 7.02 (s, 4H), 2.40 (s, 12H), 0.14 (s, 18H); ^{13}C{^{1}H} NMR (100 MHz, $CDCl_3$) δ 144.7, 140.2, 139.8, 138.9, 137.7, 137.5, 129.4, 128.3, 115.9, 21.5, 0.5; FT-IR $\tilde{\nu}$ 2917 (w), 1595 (w), 1301 (w), 1247 (m), 1040 (w), 984 (w), 923 (m), 864 (m), 833 (m), 758 (m), 718 (m), 695 (m), 628 (m) cm^{-1}. HRMS (FD) *m/z* calcd for $C_{32}H_{38}S_2Si_2$ [M$^+$] 542.1948, found 542.1953.

Synthesis of diiodo-BDT 12:

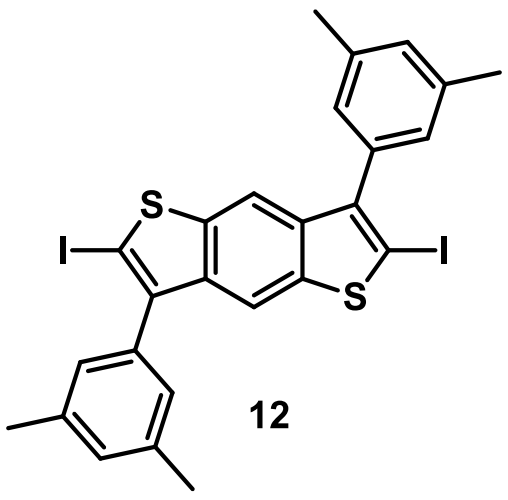

12

Compound **11** (0.30 g, 0.55 mmol) was dissolved with DCM (7 mL), and the solution was cooled to 0 °C for 10 minutes. Iodine chloride (0.22 g, 1.4 mmol) dissolved in DCM (3 mL) was slowly added to the solution. The reaction mixture was stirred at room temperature for overnight, then quenched with aqueous $NaHCO_3$. The mixture was diluted with water and the aqueous phase was extracted with DCM (3 × 20 mL). After removal of the solvent under reduced pressure, the solid was recrystallized from chloroform/methanol and washed with methanol to give **12** (0.29 mg, 79%) as a white solid. mp 291.1–291.7 °C; ^{1}H NMR (400 MHz, $CDCl_3$) δ 7.87 (s, 2H), 7.13 (s, 2H), 7.06 (s, 4H), 2.44 (s, 12H); ^{13}C{^{1}H} NMR (100 MHz, $CDCl_3$) δ 142.3, 141.1, 138.5, 136.5, 135.6, 130.2, 127.9, 115.3, 81.8, 21.6; FT-IR $\tilde{\nu}$ 2913 (w), 1732 (w), 1598 (w), 1428 (w), 1373 (w), 1321 (w), 1300 (w), 1233 (w), 1064 (w), 1028 (w), 918 (w), 868 (m), 854 (m), 821 (w), 717 (m), 688 (m) cm^{-1}. HRMS (FD) *m/z* calcd for $C_{26}H_{20}I_2S_2$ [M^{+}] 649.9090, found 649.9088.

Synthesis of 26BDT:

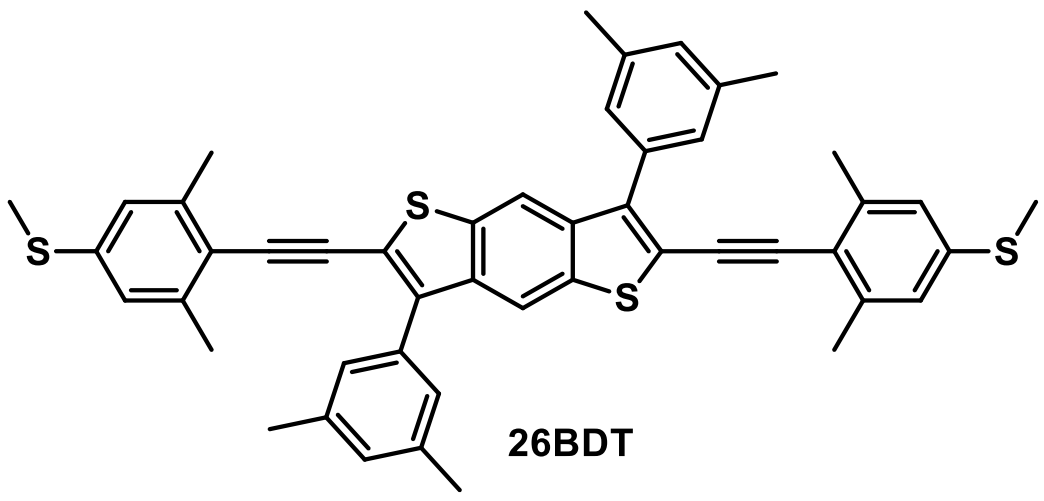

26BDT

In a 50 mL two-neck round-bottom flask, compound **12** (70 mg, 0.11 mmol), sulfane **15**[62] (47 mg, 0.27 mmol) were dissolved in 1:1 (v/v) triethylamine (6 mL) / THF (6 mL). The reaction mixture was deaerated by nitrogen bubbling and $PdCl_2(PPh_3)_2$ (8.0 mg, 0.011 mmol, 10 mol%) and CuI (2.0 mg, 0.011 mmol, 10 mol%) were added. The mixture was stirred at 70 °C for 12 h. After removal of the solvent under reduced pressure, the solid was collected by suction filtration, washed successively with methanol, acetone and pentane and dried in an oven to yield **26BDT** (70 mg, 86%) as a green solid. mp 319.5–320.1 °C; ^{1}H NMR (400 MHz, $CDCl_3$) δ 8.10 (s, 2H), 7.32 (s, 4H), 7.11 (s, 2H), 6.89 (s, 4H), 2.46 (s, 6H), 2.43 (s, 12H), 2.33 (s, 12H); ^{13}C{^{1}H} NMR (125 MHz, $C_2D_2Cl_4$, 100 °C) δ 140.7, 139.6, 139.1, 137.9, 137.6, 137.3, 134.3, 129.7, 127.4, 124.7, 120.7, 119.3, 116.3, 95.2, 91.7, 21.1,

20.6, 15.4; FT-IR $\tilde{\nu}$ 2906 (w), 2179 (w), 1589 (w), 1435 (w), 1374 (w), 1311 (w), 1236 (w), 1117 (w), 1069 (w), 840 (m), 715 (w), 688 (w) cm^{-1}. HRMS (FD) *m/z* calcd for $C_{48}H_{42}S_4$ [M^+] 746.2164, found 746.2174.

The synthetic route of anchor unit **9** is shown in Scheme S5.

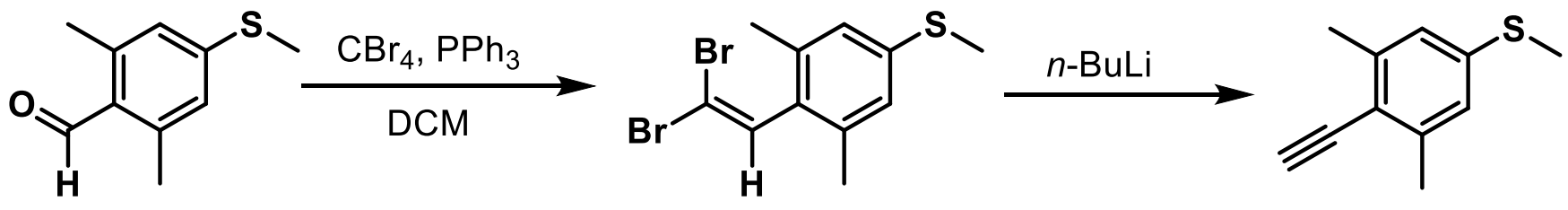

13 **14** **15** (59%)

Scheme S5. Synthetic scheme for **9** with respective yields in parentheses.

Synthesis of Compound 15

2,6-Dimethyl-4-(methylthio)benzaldehyde (**13**)[62] (2.46 g, 13.7 mmol) and PPh_3 (10.7 g, 40.9 mmol) were dissolved in DCM (68 mL) in an oven-dried 250 mL round-bottom flask and cooled to 0 °C. CBr_4 (6.80 g, 20.5 mmol) was then slowly added to the solution over a period of 3 minutes, and the mixture was maintained at the same temperature for 1 h. After warming to room temperature, hexane was added. The residue was purified by flash column chromatography on silica gel (hexane) to remove triphenylphosphine oxide, affording a crude product of **14** (3.09 g).

The crude product was dissolved in dry THF (37 mL) in an oven-dried 100 mL double neck round bottom flask. After cooling to −90 °C, *n*-BuLi (2.5 M in hexane, 9.2 mL, 23 mmol) was added to the reaction mixture via a syringe under a nitrogen atmosphere. The reaction mixture was stirred at –90 °C for 1 h. After warming to room temperature, the solution was stirred for 30 min before it was quenched by addition of saturated NH_4Cl solution. The mixture was extracted with EtOAc, and the extract was washed with water and brine, and the organic layer was dried over $MgSO_4$. After removal of the solvent under reduced pressure, the residue was purified by column chromatography on silica gel (hexane) to give **15** (1.42 g, 59%) as a yellow liquid. The ^1H NMR spectrum agreed with the literature.[62]

3. Previous Comparative Studies on Antiaromatic Systems

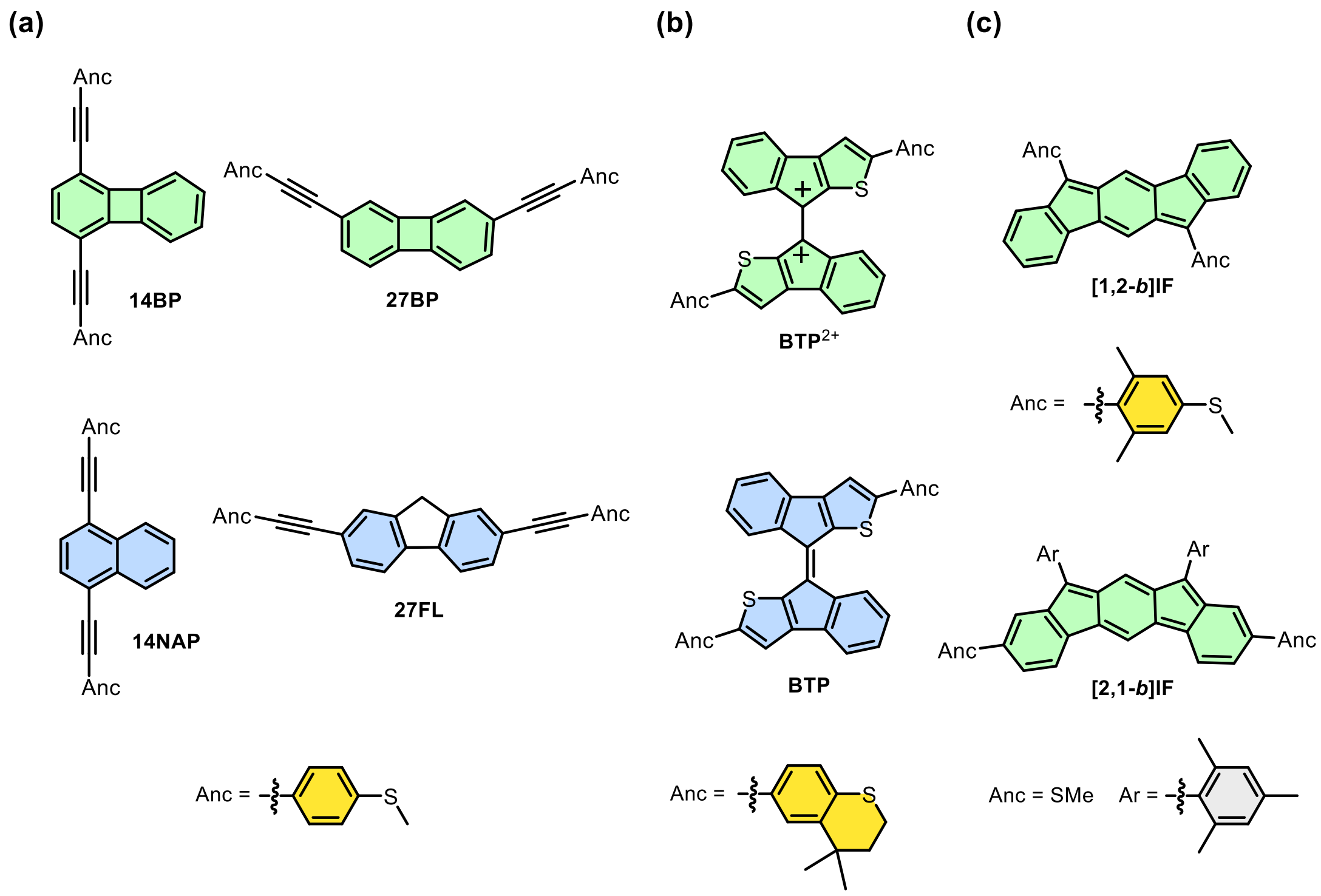

Figure S1. Representative antiaromatic compounds (turquoise) and their aromatic counterparts (light blue) with anchor groups (Anc: yellow) investigated for single-molecule *G*. (a) **14BP**/**14NAP** and **27BP**/**27FL**, (b) **BTP**$^{2+}$/**BTP**, and (c) open-shell antiaromatic compounds **[1,2-*b*]IF** and **[2,1-*b*]IF**.

A significant challenge in single-molecule electronic conductance experiments arises from the difficulty of comparing antiaromatic compounds with aromatic counterparts under identical conditions, because they typically consist of a distinct number of electrons. For example, the antiaromatic biphenylene derivatives **14BP** and **27BP** did not exhibit higher conductance than the aromatic naphthalene and fluorene analogues **14NAP** and **27FL** (Figure S1a).[65] Although the 12 π −12 π-electron antiaromatic dication of biindenothiophenylidene was shown to exhibit a conductance value approximately 70 times higher than neutral BTP (Figure S1b), the external effects of charge and counterions remain significant variables that cannot be ignored.[66] It is also important to note that while open-shell indenofluorene derivatives such as **[1,2-*b*]IF** and **[2,1-*b*]IF** possess 20 π-electron antiaromatic frameworks (Figure S1c), previous research focused primarily on their diradical character rather than explicitly evaluating the impact of antiaromaticity itself.[17-19]

4. Cyclic Voltammetry of Bridge Molecules

CVs were recorded using 1 – 5 mM of analyte in 0.1 M NBu_4PF_6/CH_2Cl_2 at a scan rate of 100 mV/s with a glassy carbon working electrode, a Pt wire counter electrode, and a Ag wire pseudo−reference electrode. The voltammograms are shown in Figure S2. The experimental HOMO and LUMO levels are estimated based on the onset potentials[67] (E_{onset}^{ox} and E_{onset}^{red}) which are determined as shown below in Table S1.

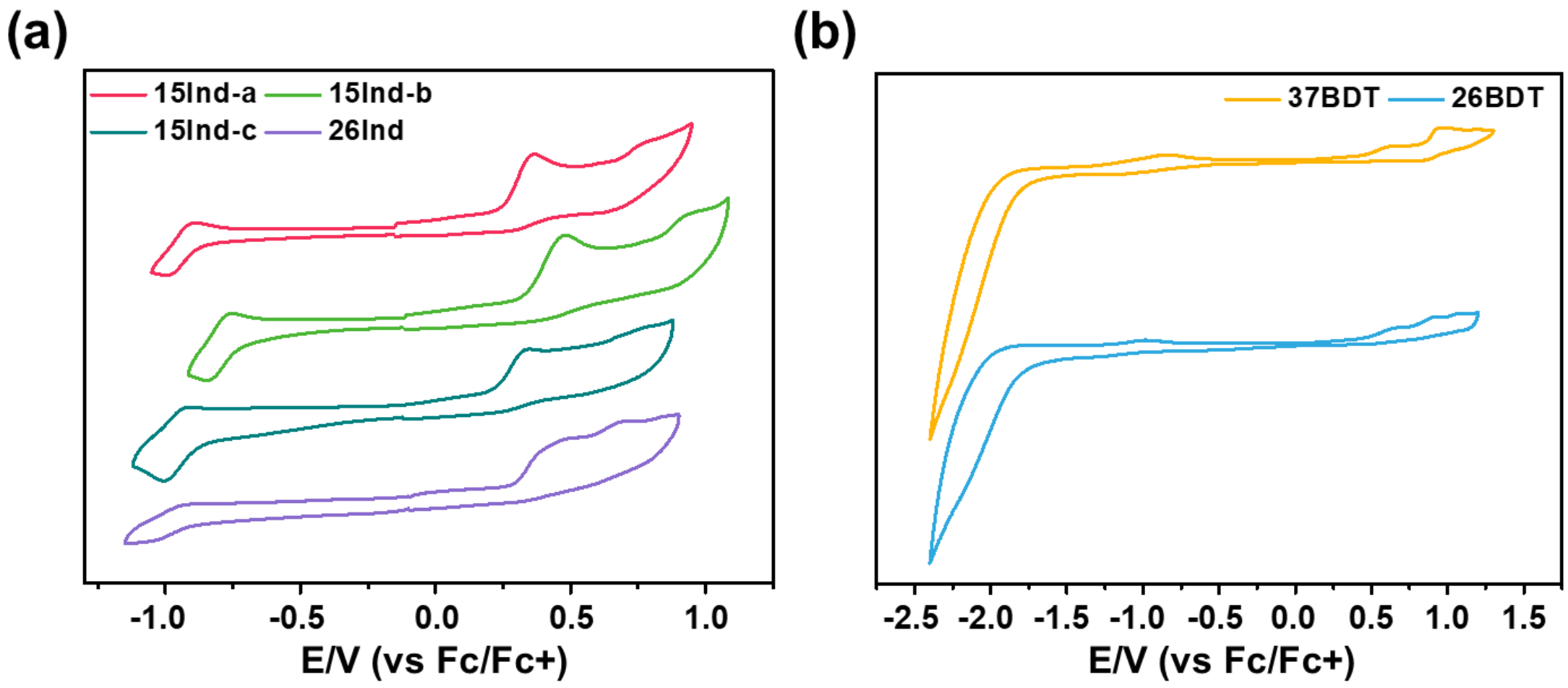

Figure S2. Cyclic voltammograms of (a) **15Ind-a, 15Ind-b, 15Ind-c**, and **26Ind**, and (b) **37BDT** and **26BDT**.

Table S1. Theoretical and Electrochemical Frontier MO Levels

Compd	Theoretical MO levels[a]					Electrochemical potentials and MO levels[b]				
	E_{HOMO-1} (eV)	E_{HOMO} (eV)	E_{LUMO} (eV)	E_{gap1}[c] (eV)	E_{gap2}[d] (eV)	E_{onset}^{ox} (V)	E_{onset}^{red} (V)	E_{HOMO} (eV)	E_{LUMO} (eV)	E_{gap} (eV)
**15Ind-a**	−4.99[e]	−4.77[f]	−3.24	1.53	1.75	0.25	−0.87	−5.05	−3.93	1.12
**15Ind-b**	−5.32[e]	−5.03[f]	−3.56	1.47	1.76	0.33	−0.70	−5.13	−4.10	1.03
**15Ind-c**	−4.93[e]	−4.67[f]	−3.17	1.50	1.76	0.22	−0.84	−5.02	−3.92	1.10
**26Ind**	−5.29[f]	−5.04[e]	−3.35	1.69	1.94	0.30	−0.85	−5.10	−3.95	1.15
**37BDT**	−5.94	−5.41	−2.04	3.37	3.90	0.45	−1.85	−5.25	−2.95	2.30
**26BDT**	−5.76	−5.24	−2.21	3.03	3.55	0.47	−1.78	−5.23	−3.02	2.21

[a]Calculations were performed at the RB3LYP/6-311G(d,p) level of theory. [b]CVs were recorded using 1−5 mM of analyte in 0.1 M NBu_4PF_6/ DCM at a scan rate of 100 mV/s with a glassy carbon working electrode, a Pt wire counter electrode, and an Ag wire pseudo-reference electrode. E^{ox} and E^{red} are the peak potentials for oxidation and reduction processes, respectively. Since all oxidation processes are irreversible and reduction processes are quasi-reversible, the experimental HOMO and LUMO levels

are estimated based on the onset potentials (E_{onset}^{ox} and E_{onset}^{red}). The HOMO and LUMO energy levels were calculated from the equation HOMO/ LUMO = −($E_{onset}^{ox/red}$ − E_{onset}(ferrocene) + 4.8) eV using ferrocence as the standard, whose HOMO is set at −4.8 eV with respect to zero vacuum level.[67] ^{c}Energy gap between HOMO and LUMO. ^{d}Energy gap between HOMO−1 and LUMO. ^{e}*gerade* MO. ^{f}*ungerade* MO.

5. Optical Properties of Bridge Molecules

Table S2. Experimental Electronic Transitions

Compd	Electronic absorption maxima^{a}			
	λ_{max1}^{b}, ε (nm, L mol^{-1} cm^{-1})	λ_{max2}^{c}, ε (nm, L mol^{-1} cm^{-1})	λ_{onset} (nm)	E_g^{opt} (eV)^{d}
**15Ind-a**	1060, 316	735, 78500	776	1.60
**15Ind-b**	1245, 151	750, 80100	802	1.55
**15Ind-c**	1197, 232	747, 73100	790	1.57
**26Ind**	– ^{e}	644, 50600	683	1.82^{f}
**37BDT**	386, 33900	–	423	2.93
**26BDT**	424, 70100	–	444	2.79

^{a}Measured in CH_2Cl_2. ^{b}Absorption maximum of the first excitation band and its molar extinction coefficient. ^{c}Absorption maximum of the second excitation band and its molar extinction coefficient. $^{d}E_g^{opt} = 1240/\lambda_{onset}$. ^{e}Not measured due to very low solubility. ^{f}Optical bandgap between HOMO−1 and LUMO.

6. Theoretical Calculations

6.1. General

DFT calculations in vacuum were performed using the Gaussian 09. Geometries were optimized at the B3LYP/6-311G(d,p) level of theory, and the optimized structures were confirmed to be minima through frequency analysis.

6.1.1. Theoretical and Experimental Electronic Transitions

Theoretical electronic transitions estimated by TD-DFT method performed at the B3LYP/6-311G(d,p) level (Table S4-S8) and the results are summarized in Table S3..

Table S3. Theoretical Electronic Transitions

Compd	Theoretical electronic transitions[a]	
	λ_1 (nm)/main MO,[b] f	λ_2 (nm)/main MO,[c] f
**15Ind-a**	1217/ HOMO–1, 0.0000	795/ HOMO, 0.8959
**15Ind-b**	1198/ HOMO–1, 0.0000	822/ HOMO, 0.9199
**15Ind-c**	1183/ HOMO–1, 0.0000	812/ HOMO, 0.8152
**26Ind**	1292/ HOMO, 0.0000	656/ HOMO–1, 0.4504
**37BDT**	407/ HOMO, 0.3880	–
**26BDT**	451/ HOMO, 1.7429	–

[a]Calculations were performed at the RB3LYP/6-311G (d,p) level of theory. [b]Wavelength of the first transition by TD-DFT calculations, main contributing occupied MO, and oscillator strength. [c]Wavelength of the second transition by TD-DFT calculations, main contributing occupied MO, and oscillator strength.

Table S4. The First Three Electronic Transitions in TD-DFT Calculations for **15Ind-a**.

Excited State	Excitation energy / eV (wavelength / nm)	Excitation amplitude	Oscillator strength f
1	1.019 (1217)	0.699 (HOMO–1 – LUMO)	0.0000
2	1.560 (795)	0.715 (HOMO – LUMO)<br>–0.158 (LUMO – HOMO)	0.8959
3	2.228 (556)	0.692 (HOMO–2 – LUMO)	0.4059

Table S5. The First Three Electronic Transitions in TD-DFT Calculations for **15Ind-b**.

Excited State	Excitation energy / eV (wavelength / nm)	Excitation amplitude	Oscillator strength *f*
1	1.035 (1198)	0.699 (HOMO–1 – LUMO)	0.0000
2	1.508 (822)	0.717 (HOMO – LUMO)<br>–0.163 (LUMO – HOMO)	0.9199
3	2.159 (574)	0.693 (HOMO–2 – LUMO)	0.0000

Table S6. The First Three Electronic Transitions in TD-DFT Calculations for **15Ind-c**.

Excited State	Excitation energy / eV (wavelength / nm)	Excitation amplitude	Oscillator strength *f*
1	1.048 (1183)	0.697 (HOMO–1 – LUMO)	0.0000
2	1.526 (812)	0.716 (HOMO – LUMO)<br>–0.159 (LUMO – HOMO)	0.8152
3	2.140 (579)	0.698 (HOMO–2 – LUMO)	0.5755

Table S7. The First Three Electronic Transitions in TD-DFT Calculations for **26Ind**.

Excited State	Excitation energy / eV (wavelength / nm)	Excitation amplitude	Oscillator strength *f*
1	0.960 (1292)	0.701 (HOMO – LUMO)	0.0000
2	1.891 (656)	0.696 (HOMO–1 – LUMO)<br>0.139 (HOMO – LUMO+1)<br>–0.158 (LUMO – HOMO–1)	0.4504
3	2.159 (574)	0.702 (HOMO–2 – LUMO)	0.0994

Table S8. The First Three Electronic Transitions in TD-DFT Calculations for **37BDT**.

Excited State	Excitation energy / eV (wavelength / nm)	Excitation amplitude	Oscillator strength *f*
1	3.048 (407)	0.698 (HOMO – LUMO)	0.3880
2	3.286 (377)	0.701 (HOMO – LUMO+1)	0.0018
3	3.456 (359)	0.699 (HOMO–1 – LUMO)	0.0022

Table S9. The First Three Electronic Transitions in TD-DFT Calculations for **26BDT**.

Excited State	Excitation energy / eV (wavelength / nm)	Excitation amplitude	Oscillator strength *f*
1	2.747 (451)	0.702 (HOMO – LUMO)	1.7429
2	3.188 (389)	0.220 (HOMO–2 – LUMO)<br>0.655 (HOMO–1 – LUMO)	0.1227
3	3.326 (373)	0.495 (HOMO–2 – LUMO)<br>–0.130 (HOMO–1 – LUMO)<br>–0.476 (HOMO – LUMO+1)	0.3542

6.1.2. Diradical Characters

The degree of diradical character (y_0) was calculated using the LC-UBLYP/6-311G(d,p) method, setting the μ-parameter (range-separation parameter), from the default value of 0.4 $bohr^{-1}$ to 0.33 $bohr^{-1}$. Here, y_0 is defined according to the Yamaguchi scheme[42], as shown in equation (1), where T (representing the orbital overlap) is evaluated from the occupation numbers (n) of the highest occupied nonbonding orbital (HONO) and the lowest unoccupied nonbonding orbital (LUNO), as defined in equation (2).

$$y_0 = 1 - \frac{2T}{1+T^2} \qquad (1)$$

$$T = \frac{n_{HONO} - n_{LUNO}}{2} \qquad (2)$$

Table S10. Diradical Character (y_0) of **15Ind-a**, **15Ind-b**, **15Ind-c**, and **26Ind**.

**Compd**	***n*HONO**	***n*LUNO**	***T***	$\boldsymbol{y_0}$
**15Ind-a**	1.510	0.490	0.510	0.191
**15Ind-b**	1.506	0.494	0.506	0.194
**15Ind-c**	1.510	0.490	0.510	0.190
**26Ind**	1.631	0.369	0.631	0.097

6.1.3. NICS Calculations

$NICS(1)_{zz}$ calculations were performed at the B3LYP/6-311G(d,p) level.

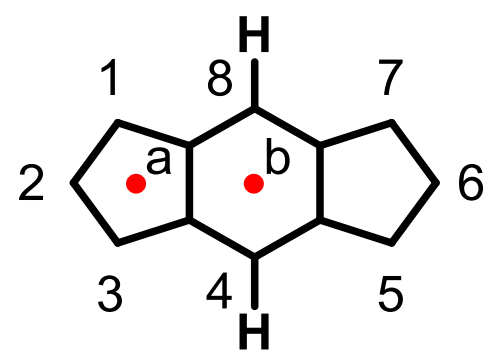

Figure S3. Positions (a, center of the five-membered ring and b, center of the six-membered ring) for the $NICS(1)_{zz}$ calculation and carbon numbering for the ^{1}H NMR chemical shift in Table S11.

Table S11. $NICS(1)_{zz}$ Values and ^{1}H NMR Chemical Shifts of **15Ind-a, 15Ind-b, 15Ind-c**, and **26Ind**.

Compd	$NICS(1)_{zz}$ / ppm		^{1}H Chemical Shift / ppm	
	a^a	b^b	Calcd^c	Obs^d
**15Ind-a**	33.6	23.0	6.07	6.67
**15Ind-b**	32.1	21.4	6.04	6.62
**15Ind-c**	31.4	20.4	6.23	6.81
**26Ind**	35.0	26.7	5.96	6.63

[a]At the five-membered ring center (Figure S3). [b]At the six-membered ring center (Figure S3). [c]Calculations were performed at the RB97-2/6-311G(d,p) level of theory. [d]Measured in $CDCl_3$ at 25 °C.

6.2. NEGF-DFT Calculations

The nonequilibrium Green's function method combined with density functional theory (NEGF-DFT) calculations were performed using the SMEAGOL code[68-70] and SIESTA package[71]. Single zeta plus polarization (SZP) and double zeta plus polarization (DZP) basis sets were used for Au and other atoms, respectively. We used the Perdew-Burke-Ernzerhof (PBE) exchange-correlation functional.[72] The electrode was modeled as an Au(111) slab with the p(6×6) periodicity, and the S atoms of $-SCH_3$ anchors adsorb on top of the Au atoms. The k points were sampled using a 2×2×1 uniform grid. A molecular-projected self-consistent Hamiltonian (MPSH) analysis was performed to investigate the contribution of molecular orbitals to transmission functions. The results are summarized in Section S8.

7. STM-BJ Experiments

7.1 Hardware Details

The STM-BJ measurement was performed using a PicoScan 2500 SPM controller (Molecular Imaging Inc.) controlled with PicoScan 5.3.3 software, using the I-S spectroscopy function of the software. A conductance trace was measured in 0.1 second with a resolution of 2,000 points. The electrical current was measured using a FEMTO DLPCA200 with current-voltage converter at a sensitivity of 1 μA/V. To enhance sensitivity to 0.01 μA/V, the amplifier's internal gain (100×) was used. The device was modified to output both signals, providing the dynamic range required for the measurements. For the data analysis, the electrical current signals from the1 μA/V and 0.01 μA/V channels were merged at $10^{-3}G_0$.

7.2. Experimental Details

STM-BJ measurements were carried out with a commercial STM (PicoSPM, Molecular Imaging Inc.). Au STM tips were prepared by cutting Au wires. Au substrates were 100 nm thick Au thin films prepared by thermal evaporation of Au on mica substrates whose temperature was kept at 570 K during evaporation. The substrates were cut into small pieces, gently cleaned with 2-propanol, and dried under Ar gas. Subsequently, the substrates were exposed to ozone for 30 minutes using an ozone cleaner (NL-UV253S, Nihon Laser Denshi Co., Ltd.) to remove organic contaminants. Before molecular modification, the substrate surface was subjected to a flame of butane burner to remove contaminants. The substrates were immersed in a 1 mM dichloromethane solution of each molecule for 10 min, then dried under an Ar atmosphere. For **37BDT**, sufficient molecular junction formation was not observed by immersion in the molecular solution. Therefore, the procedure of dropping the molecular solution onto the substrate followed by drying was repeated until a molecular junction was observed in the STM-BJ measurement.

STM-BJ measurements were conducted in Ar atmosphere at room temperature with bias voltages of 100 mV, 200 mV, and 500 mV.

7.3. Extraction of Conductance Traces

For the analysis, the conductance traces showing clear contact formation and rupture between the STM tip and the substrate were selected. Figure S4 illustrates the data analysis procedure using the measurement results for **15Ind-a** at a bias voltage of 200 mV.

Figure S4 (a) shows a two-dimensional (2D) conductance histogram constructed from the raw data without any trace selection. The horizontal axis corresponds to the tip displacement, and the

vertical axis represents logarithmic electrical conductance, with 200 bins used for each axis. A conductance plateau is observed in the 2D histogram. Based on this observation, the logarithmic conductance range corresponding to the plateau was determined to be between −3 and −5.

To more clearly identify the plateau position in the 2D histogram, conductance traces containing plateaus were selected for further analysis following the procedure described below. As shown in Figure S4 (b), a one-dimensional (1D) conductance histogram with 30 bins was constructed for each trace within the conductance range of the plateau identified in the 2D histogram. Traces with bin counts exceeding a threshold value of 30 were selected. Figure S4 (c) shows a 2D conductance histogram constructed from the selected traces. As a result of the trace selection procedure, the conductance plateau becomes more clearly visible due to the removal of traces dominated by tunneling currents without molecular bridging.

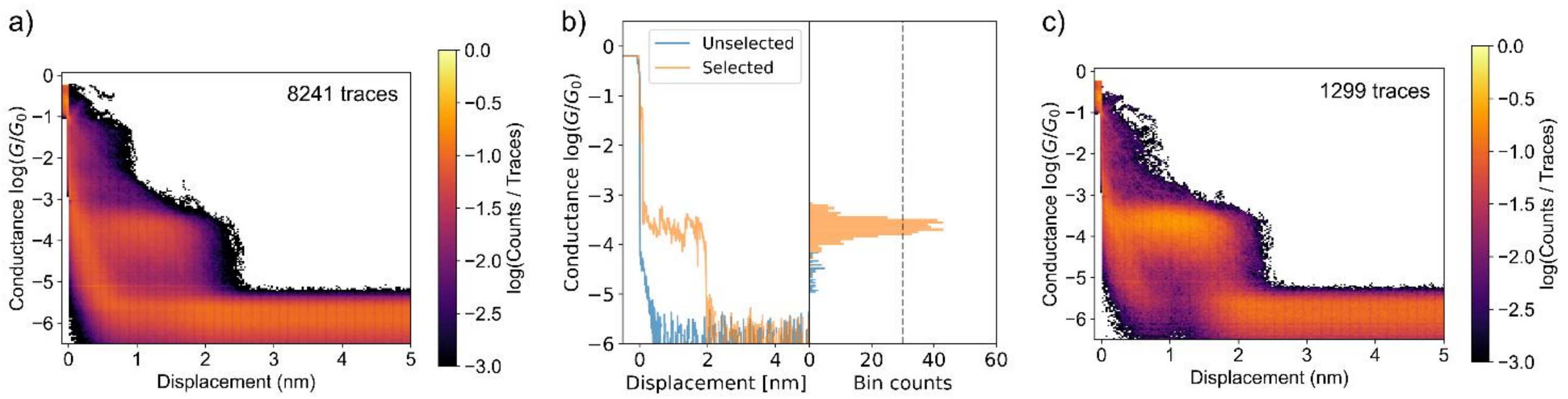

Figure S4. (a) 2D conductance histogram constructed from the raw data without trace selection (8241 traces) for **15Ind-a** at a bias voltage of 200 mV. (b) Representative individual conductance traces and the corresponding 1D conductance histograms generated from each trace; the dashed line denotes the threshold criterion used for trace selection. (c) 2D conductance histogram constructed from the selected traces (1299 traces).

7.4. Conductance Analysis

The conductance of the molecular junctions was evaluated using 1D conductance histograms constructed from the selected traces. Gaussian fitting was performed on the peak corresponding to the conductance plateau, and the peak value was defined as the conductance of the molecular junction. In the measurements at a bias voltage of 100 mV, in addition to the peak corresponding to the molecular junction, a peak attributed to the single-atom contact of the gold electrodes was observed near 1 G_0. Figures S5 and S6 show the 1D and 2D histograms used for the analysis. Table S12 summarizes the conductance values obtained from the analysis. It should be noted that the clear plateaus for **37BDT** were not observed at a bias voltage of 100 mV due to its low conductance.

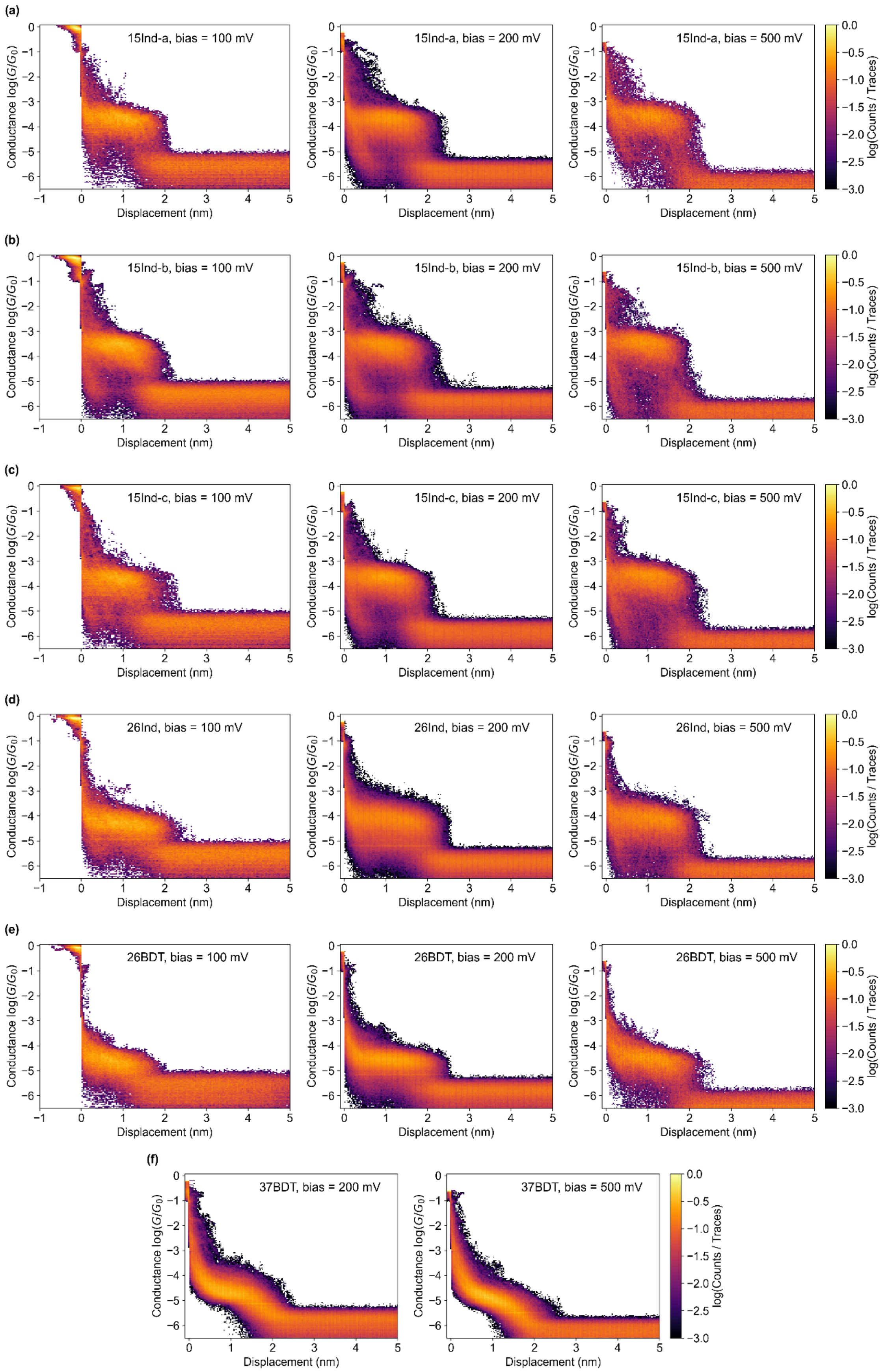

Figure S5. 2D conductance histograms at 100 mV, 200 mV, and 500 mV for (a) **15Ind-a**, (b) **15Ind-b**, (c) **15Ind-c**, (d) **26Ind**, and (e) **26BDT**, and at 200 mV and 500 mV for (f) **37BDT**.

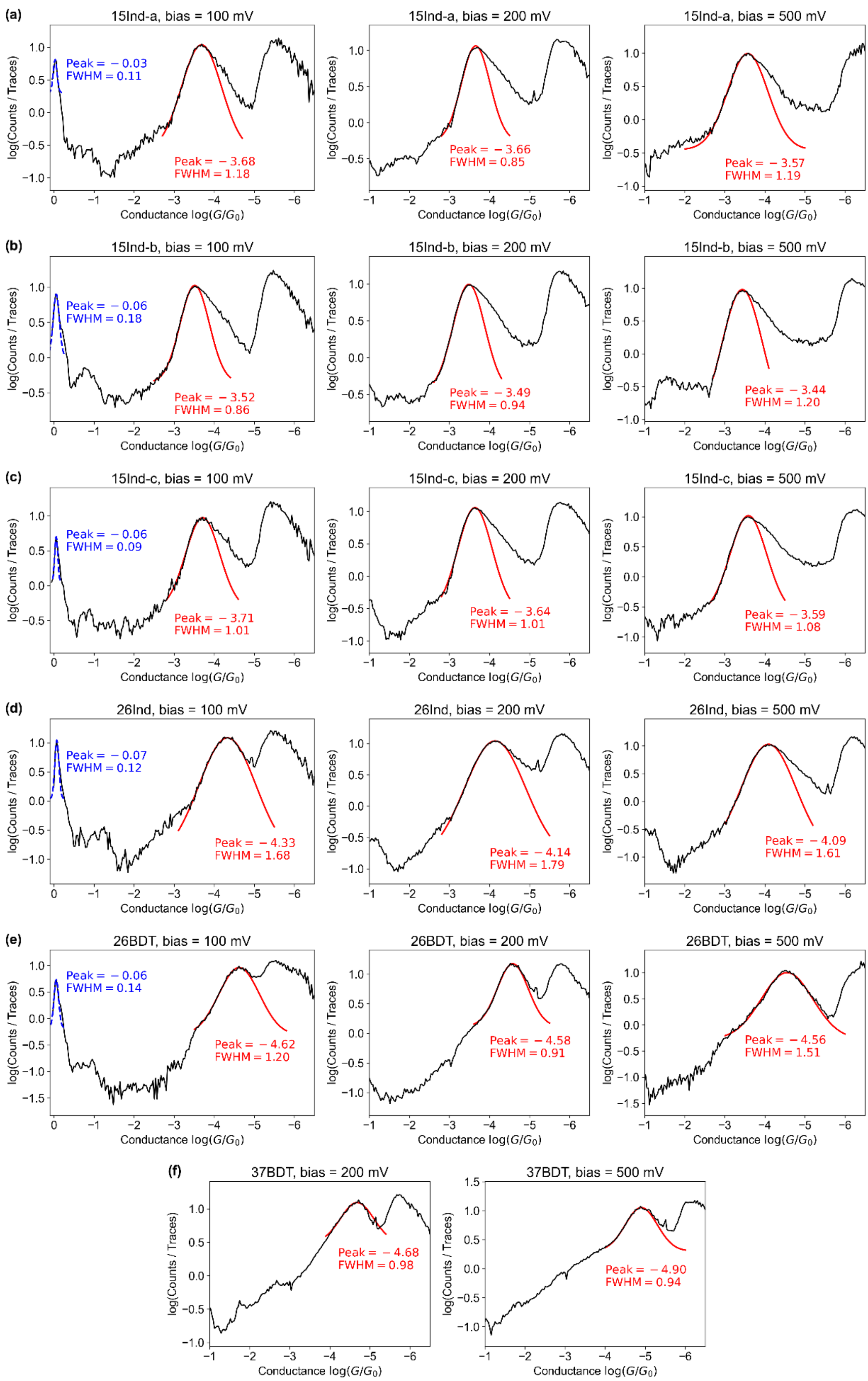

Figure S6. 1D conductance histograms with fitted Gaussian function at 100 mV, 200 mV, and 500 mV for (a) **15Ind-a**, (b) **15Ind-b**, (c) **15Ind-c**, (d) **26Ind**, and (e) **26BDT**, and at 200 mV and 500 mV for (f) **37BDT**.

Table S12. Summary of log(G/G_0) and FWHM.

Compd	Measured conductance (log(G/G_0))[a]		
	100 mV	200 mV	500 mV
**15Ind-a**	−3.68 (1.18)	−3.66 (0.85)	−3.57 (1.19)
**15Ind-b**	−3.52 (0.86)	−3.49 (0.94)	−3.44 (1.20)
**15Ind-c**	−3.71 (1.01)	−3.64 (1.01)	−3.59 (1.08)
**26Ind**	−4.33 (1.68)	−4.14 (1.79)	−4.09 (1.61)
**37BDT**	-[b]	−4.68 (0.98)	−4.90 (0.94)
**26BDT**	−4.62 (1.20)	−4.58 (0.91)	−4.56 (1.51)

[a]Values in parentheses are the full width at half maximum (FWHM) of the peak.

[b]Not available because the plateaus were in the noise level.

Table S13. Number of Raw and Selected Traces at Different Bias Voltages.

Compd	Bias voltage (mV)	Number of raw traces	Number of selected traces
**15Ind-a**	100	998	284 (29%)
	200	8241	1299 (16%)
	500	959	247 (26%)
**15Ind-b**	100	1839	532 (29%)
	200	7034	1220 (17%)
	500	1672	434 (26%)
**15Ind-c**	100	2030	289 (14%)
	200	2707	1151 (43%)
	500	2642	590 (22%)
**26Ind**	100	1977	251 (13%)
	200	11683	1548 (13%)
	500	1502	520 (35%)
**37BDT**	200	12662	1373 (11%)
	500	1836	1280 (70%)
**26BDT**	100	931	296 (32%)
	200	12503	1119 (9%)
	500	988	339 (34%)

7.5. Plateau Length Analysis

Plateau lengths were analyzed according to the procedure reported.[32] Two conductance thresholds, G_{INI} and G_{BK}, were defined to determine the initiation and breaking points of the plateaus, respectively. G_{INI} was set to approximately 3×10^{-3} G_0, while G_{BK} was set above the noise floor determined from 1D and 2D conductance histograms (approximately 6×10^{-6} G_0). The plateau length was calculated as the distance between the positions corresponding to G_{INI} and G_{BK}. Statistical values were determined from the peaks of Gaussian functions fitted to the plateau length histograms.

Figures displaying the plateau length histograms and the corresponding Gaussian fits for each molecule are provided as Figure S7 (molecular labels are indicated at the top of each panel). The detailed distance data are summarized in Tables 1 and 2, which include both the measured plateau lengths and the total distances adjusted for a 0.5 nm snap-back correction.

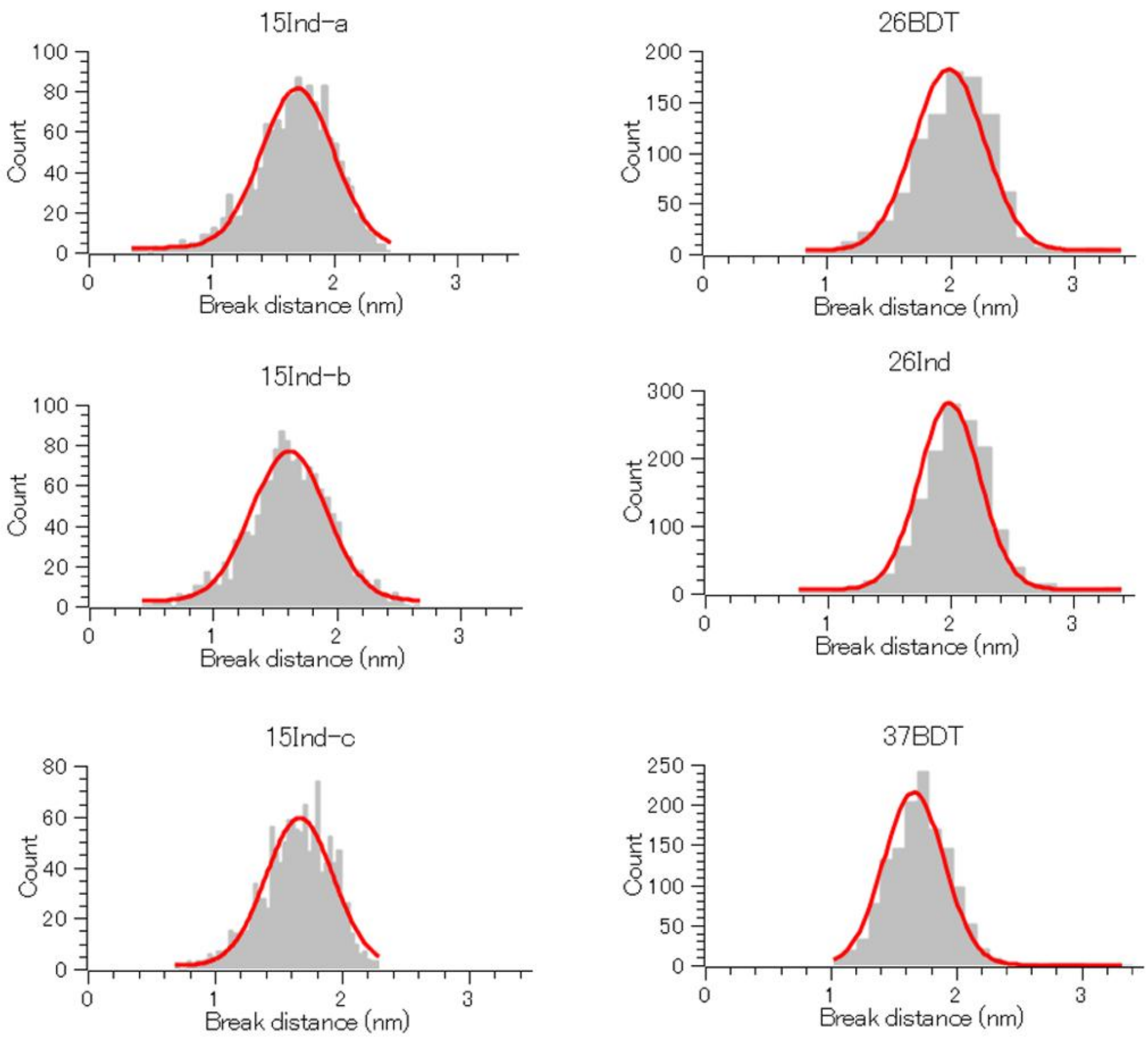

Figure S7. Plateau length distributions. Molecular labels are indicated in each panel.

8. Results of Transport Calculations

Figures S8 and Figures S9 show transmission function and the projected density of states (PDOS) of MPSH-MOs for all the molecule-junctions. The relation between the transmission and frontier orbitals of molecules, the LUMO and the HOMO, were found from these figures and indicated as L and H, respectively in Figures S8. Both **15Ind-a** and **26Ind** have smaller energy gaps between transmission peaks around the Fermi level due to antiaromaticity than the aromatic **37BDT** and **26BDT**. However, in spite of the small HOMO-LUMO gap, the LUMO peak is significantly small for **26Ind**, which is clarified by the logarithmic plot of the transmission function of **26Ind** as Figure S10. To reveal the origin of the suppression of LUMO transport, the MPSH-MO distributions of frontier orbitals are drawn in Figure S11. As displayed in Figure S11(b), the LUMO of **26Ind** is not distributed at anchoring groups, inhibiting the transport through LUMO. This absence of the wavefunction distribution stems from the fact that the connection to the anchoring group locates on the node of the wavefunction. On the other hand, the LUMO of **15Ind-a** is extended to the anchoring groups. These results indicate that both antiaromaticity and wavefunction symmetries are crucial for enhancing single-molecular conductance. Except **15-Ind** series, the peaks in the transmission close to the Fermi level originate from the LUMO and the HOMO, indicating that these MOs are responsible for the charge transport. For **15-Ind** series, HOMO-1 also contributes to the transport together with HOMO.

The major role of LUMO in most cases was deduced based on the reason described above, because MO level alignment with respect to the Femi level cannot be exactly determined by DFT calculations, primarily due to the underestimation of the HOMO-LUMO gap, which induces the overestimation of the calculated conductance values. Though several methodologies to correct the error have been proposed, such as self-interaction correction,[73] range-separated hybrid functional,[74] and DFT+Σ,[75] all these methods generally shift HOMO and LUMO away from the Fermi level, while they do not alter the dominant conducting MO (See Fig. 5 of reference 72). In particular, the energy shift is normally more effective for HOMO than LUMO (See Fig. 1 of reference 73). Therefore, when DFT predicts LUMO-mediate transport, it is unlikely that these corrections affect the transport channel.

In addition, the tiny peaks at 0 eV and 0.3 eV deserve mentioning. We assigned the transmission peaks based on the projected density of states of MPSH. The tiny peaks at 0 eV and 0.3 eV in Figures 5c, 6c, S8, and S10 are not attributed to the LUMO PDOS, though the peaks appear in all transmission spectra. We attribute the peaks at 0 eV and 0.3 eV to the hybridization between the -SCH_3 group and the Au surface, because such a peak was not observed in -S junctions.[76]

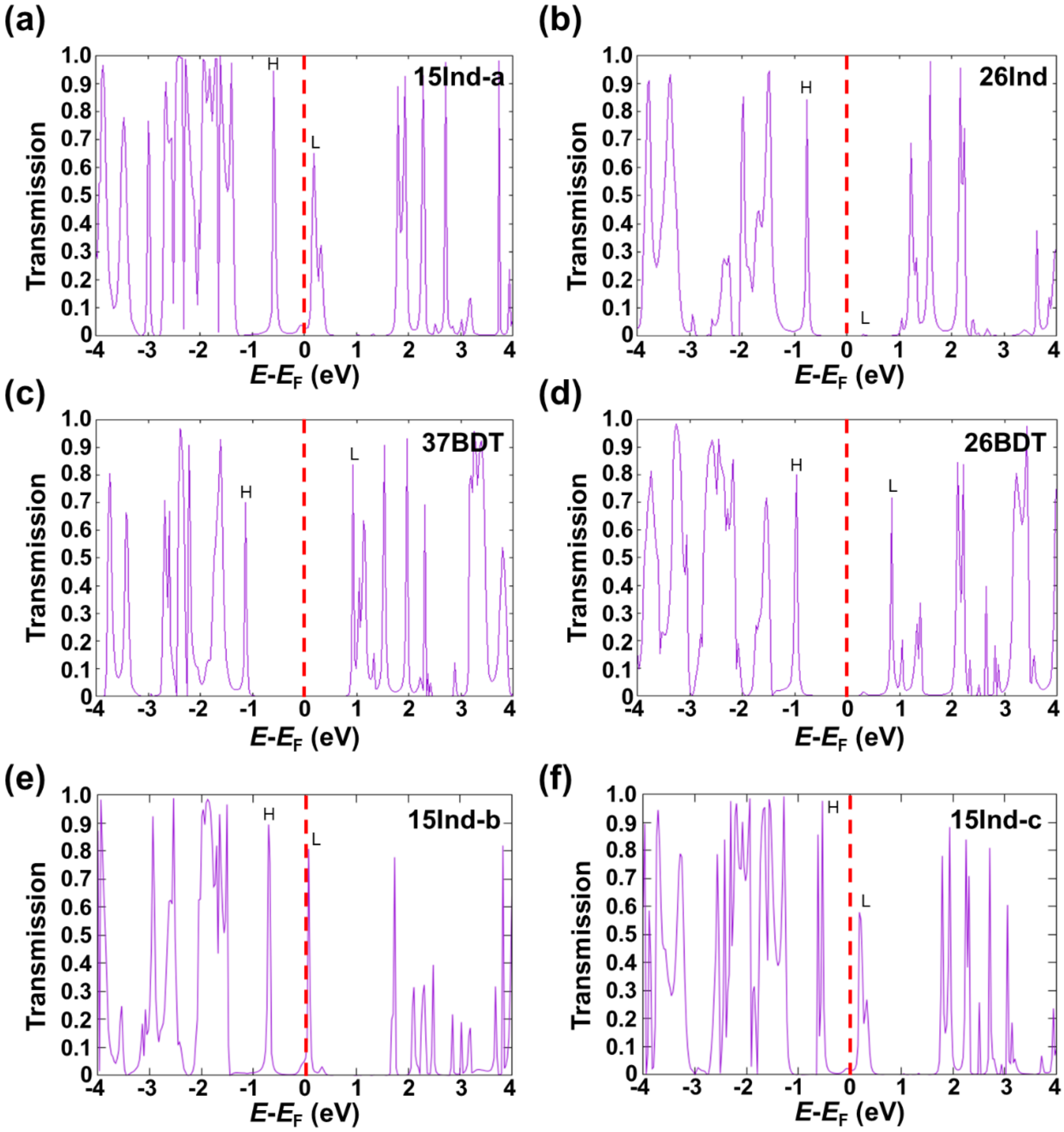

Figure S8. Calculated zero-bias transmission spectra for (a) **15Ind-a**, (b)**26Ind**, (c)**37BDT,** (d) **26BDT**, (e) **15Ind-b**, and (f) **15Ind-c** molecular junctions. The transmission value at the Fermi level (E_F, indicated by the red dashed line at 0 eV) determines the molecular conductance. H and L (shown in gray) denote the HOMO and LUMO, respectively.

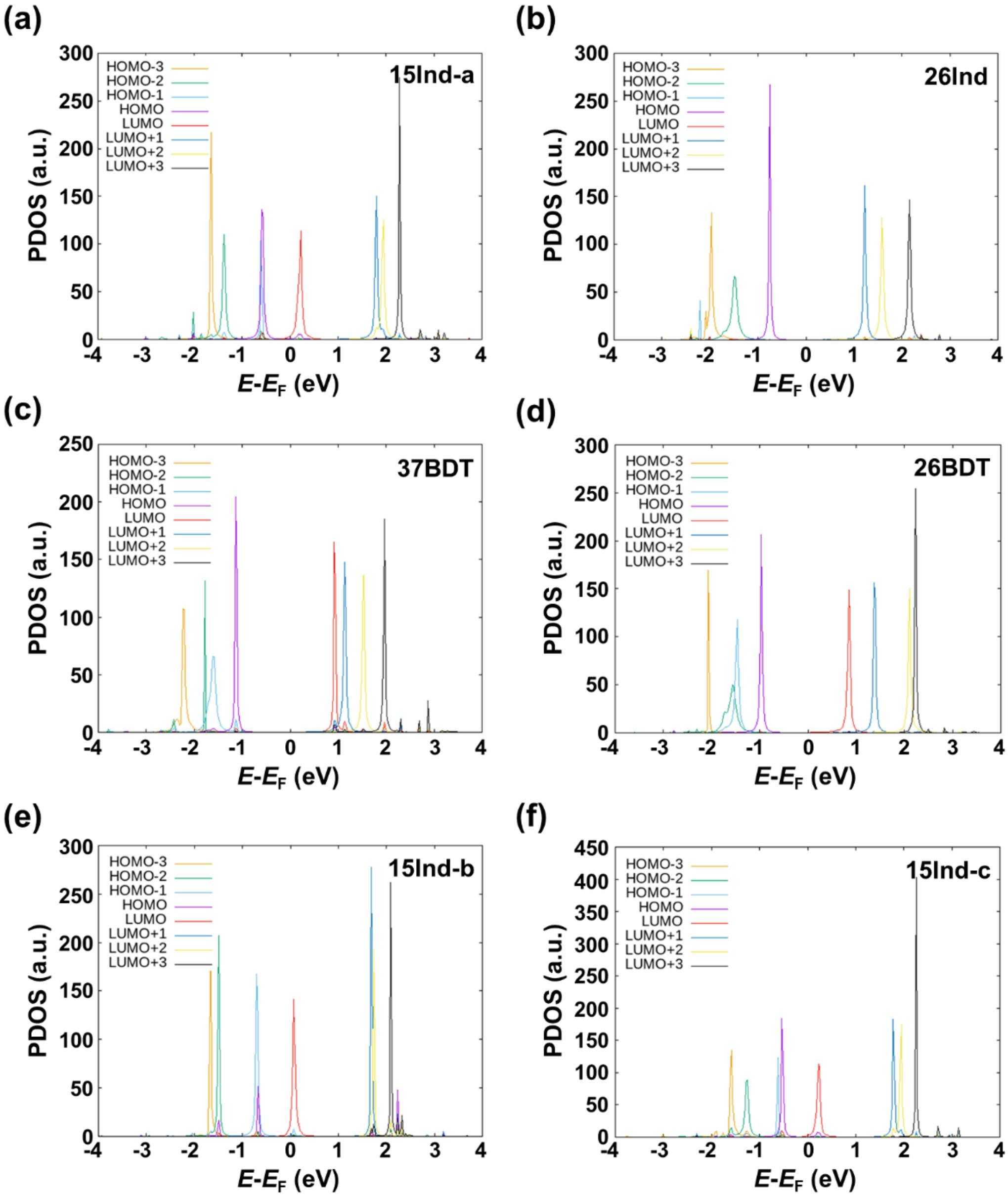

Figure S9. PDOS of MPSH-MOs (a) **15Ind-a**, (b)**26Ind**, (c)**37BDT,** (d) **26BDT**, (e) **15Ind-b**, and (f) **15Ind-c** molecular junctions.

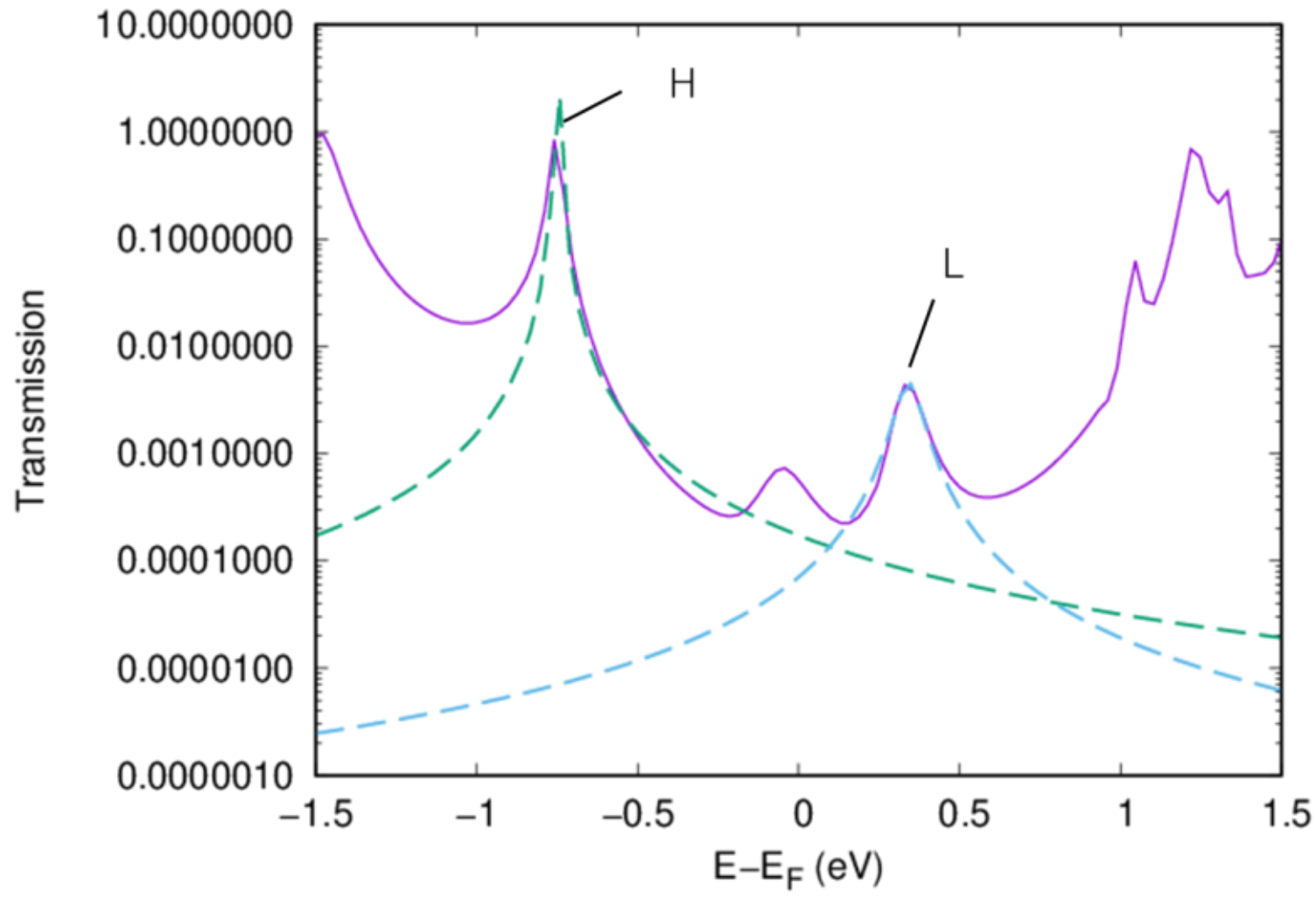

Figure S10. Magnified view of the calculated zero-bias transmission spectrum for **26Ind** near the Fermi level. The red dashed curve highlights the HOMO peak. H and L (shown in gray) denote the HOMO and LUMO, respectively.

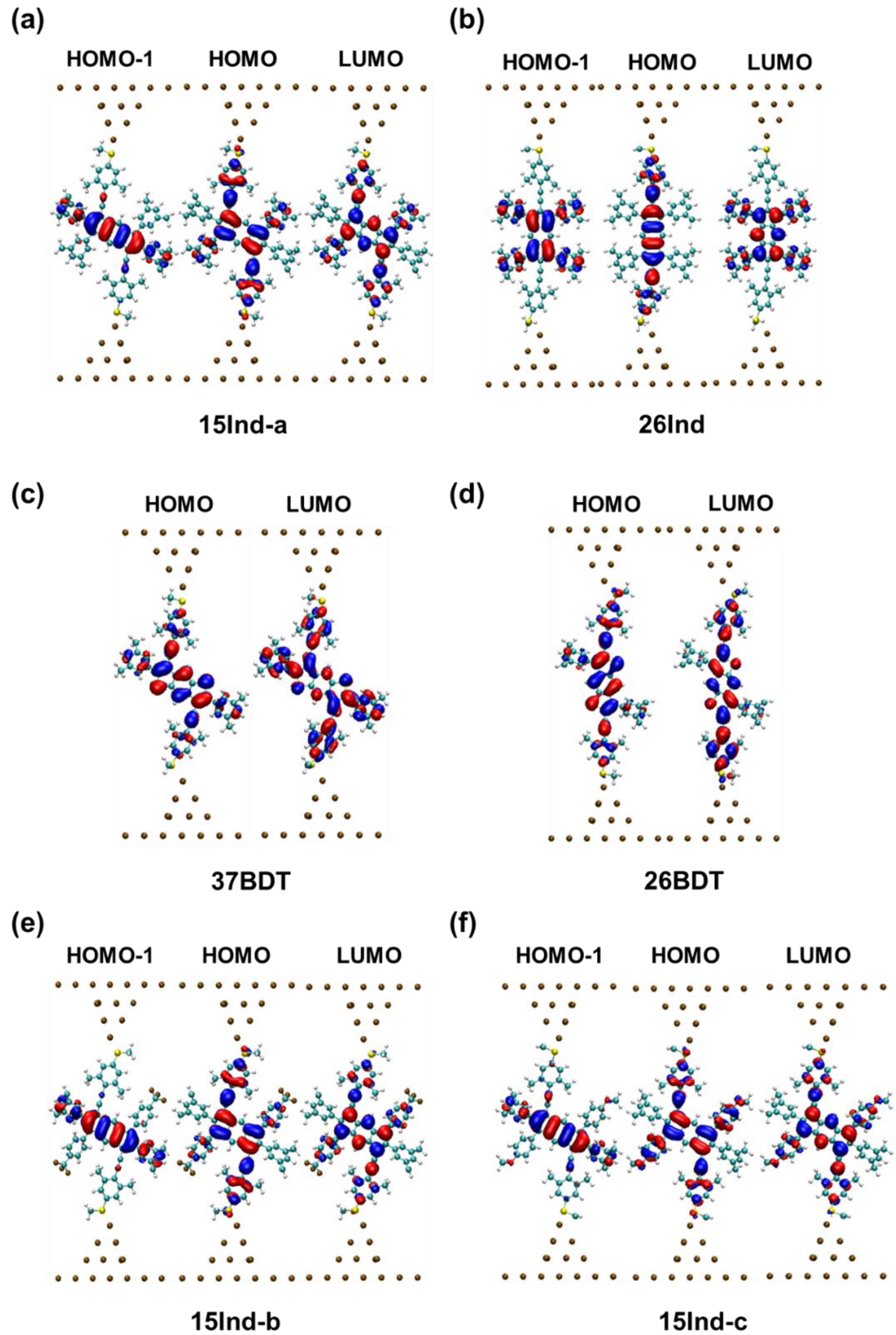

Figure S11. Calculated MPSH-MO distributions for (a) **15Ind-a**, (b) **26Ind**, (c) **37BDT**, (d) **26BDT**, (e) **15Ind-b**, and (f) **15Ind-c** bonded to gold electrodes.

9. References

[17] B. Lawson, E. Vidal, Jr., S. Luna, M. M. Haley, M. Kamenetska, Extreme Anomalous Conductance Enhancement in Neutral Diradical Acene-like Molecular Junctions, *ACS Nano* **2024**, *18*, 29059-29066. https://doi.org/10.1021/acsnano.4c10183

[18] A. Sil, L. Hamilton, J. M. F. Morris, A. H. S. Daaoub, J. H. H. Burrows, C. M. Robertson, et al., Zero-Bias Anti-Ohmic Behaviour in Diradicaloid Molecular Wires, *Angew. Chem. Int. Ed.* **2024**, *63*, e202410304. https://doi.org/10.1002/anie.202410304

[19] R. Casares, S. Rodríguez-González, Á. Martínez-Pinel, I. R. Márquez, M. T. González, C. Díaz, et al., Single-Molecule Conductance of Neutral Closed-Shell and Open-Shell Diradical Indenofluorenes, *J. Am. Chem. Soc.* **2024**, *146*, 29977-29986. https://doi.org/10.1021/jacs.4c13551

[32] M. Schmidt, L. Abellán Vicente, M. T. González, L. A. Zotti, B. Esser, E. Leary, Low-lying LUMO Boosts Conductance in Antiaromatic Dibenzopentalene Versus Aromatic Analogues, *Chem. - Eur. J.* **2024**, *30*, e202400935. https://doi.org/10.1002/chem.202400935

[36] S.-J. Jhang, J. Pandidurai, C.-P. Chu, H. Miyoshi, Y. Takahara, M. Miki, et al., s-Indacene Revisited: Modular Synthesis and Modulation of Structures and Molecular Orbitals of Hexaaryl Derivatives, *J. Am. Chem. Soc.* **2023**, *145*, 4716-4729. https://doi.org/10.1021/jacs.2c13159

[42] K. Yamaguchi, The electronic structures of biradicals in the unrestricted Hartree-Fock approximation, *Chem. Phys. Lett.* **1975**, *33*, 330-335. https://doi.org/10.1016/0009-2614(75)80169-2

[59] W. Zhong, M. Li, Y. Jin, H. Jiang, W. Wu, Synthesis of 2,5-disubstituted selenophenes via a copper-catalyzed regioselective [2+2+1] cyclization of terminal alkynes and selenium, *Chem. Commun.* **2022**, *58*, 6522-6525. http://dx.doi.org/10.1039/D2CC00958G

[60] L. Wang, L. Ackermann, Ruthenium-catalyzed ortho-C–H halogenations of benzamides, *Chem. Commun.* **2014**, *50*, 1083-1085. http://dx.doi.org/10.1039/C3CC47852A

[61] D. Marcos-Atanes, C. Vidal, C. D. Navo, F. Peccati, G. Jiménez-Osés, J. L. Mascareñas, Iridium-Catalyzed ortho-Selective Borylation of Aromatic Amides Enabled by 5-Trifluoromethylated Bipyridine Ligands, *Angew. Chem. Int. Ed.* **2023**, *62*, e202214510. https://doi.org/10.1002/anie.202214510

[62] R. Casares, Á. Martínez-Pinel, S. Rodríguez-González, I. R. Márquez, L. Lezama, M. T. González, et al., Engineering the HOMO–LUMO gap of indeno[1,2-b]fluorene, *J. Mater. Chem. C* **2022**, *10*, 11775-11782. http://dx.doi.org/10.1039/D2TC02475F

[63] H. Ebata, E. Miyazaki, T. Yamamoto, K. Takimiya, Synthesis, Properties, and Structures of Benzo[1,2-b:4,5-b‘]bis[b]benzothiophene and Benzo[1,2-b:4,5-b‘]bis[b]benzoselenophene, *Org. Lett.* **2007**, *9*, 4499-4502. https://doi.org/10.1021/ol701815j

[64] K. Takimiya, Y. Konda, H. Ebata, N. Niihara, T. Otsubo, Facile Synthesis, Structure, and Properties of Benzo[1,2-b:4,5-b‘]dichalcogenophenes, *J. Org. Chem.* **2005**, *70*, 10569-10571. https://doi.org/10.1021/jo051812m

[65] M. Gantenbein, X. Li, S. Sangtarash, J. Bai, G. Olsen, A. Alqorashi, et al., Exploring antiaromaticity in single-molecule junctions formed from biphenylene derivatives, *Nanoscale* **2019**, *11*, 20659-20666. http://dx.doi.org/10.1039/C9NR05375A

[66] X. Yin, Y. Zang, L. Zhu, J. Z. Low, Z.-F. Liu, J. Cui, et al., A reversible single-molecule switch based on activated antiaromaticity, *Sci. Adv.* **2017**, *3*, eaao2615. https://doi.org/10.1126/sciadv.aao2615

[67] J. Pommerehne, H. Vestweber, W. Guss, R. F. Mahrt, H. Bässler, M. Porsch, et al., Efficient two layer leds on a polymer blend basis, *Adv. Mater.* **1995**, *7*, 551-554. https://doi.org/10.1002/adma.19950070608

[68] A. R. Rocha, V. M. García-Suárez, S. Bailey, C. Lambert, J. Ferrer, S. Sanvito, Spin and molecular electronics in atomically generated orbital landscapes, *Phys. Rev. B* **2006**, *73*, 085414. https://link.aps.org/doi/10.1103/PhysRevB.73.085414

[69] I. Rungger, S. Sanvito, Algorithm for the construction of self-energies for electronic transport calculations based on singularity elimination and singular value decomposition, *Phys. Rev. B* **2008**, *78*, 035407. https://link.aps.org/doi/10.1103/PhysRevB.78.035407

[70] T. Ohto, I. Rungger, K. Yamashita, H. Nakamura, S. Sanvito, Ab initio theory for current-induced molecular switching: Melamine on Cu(001), *Phys. Rev. B* **2013**, *87*, 205439. https://link.aps.org/doi/10.1103/PhysRevB.87.205439

[71] M. S. José, A. Emilio, D. G. Julian, G. Alberto, J. Javier, O. Pablo, et al., The SIESTA method for ab initio order-N materials simulation, *J. Phys. Condens. Matter* **2002**, *14*, 2745. https://doi.org/10.1088/0953-8984/14/11/302

[72] J. P. Perdew, K. Burke, M. Ernzerhof, Generalized Gradient Approximation Made Simple, *Phys. Rev. Lett.* **1996**, *77*, 3865-3868. https://link.aps.org/doi/10.1103/PhysRevLett.77.3865

[73] C. Toher, S. Sanvito, Effects of self-interaction corrections on the transport properties of phenyl-based molecular junctions, *Phys. Rev. B* **2008**, *77*, 155402. https://link.aps.org/doi/10.1103/PhysRevB.77.155402

[74] A. Yamada, Q. Feng, A. Hoskins, K. D. Fenk, B. D. Dunietz, Achieving Predictive Description

of Molecular Conductance by Using a Range-Separated Hybrid Functional, *Nano Lett.* **2016**, *16*, 6092-6098. https://doi.org/10.1021/acs.nanolett.6b02241

[75] J. G. Fallaque, S. Rodríguez-González, F. Martín, C. Díaz, Self-energy corrected DFT-NEGF for conductance in molecular junctions: an accurate and efficient implementation for TRANSIESTA package applied to Au electrodes, *J. Phys. Condens. Matter* **2022**, *34*, 435901. https://doi.org/10.1088/1361-648X/ac89c4

[76] R. Yamada, K. Albrecht, T. Ohto, K. Minode, K. Yamamoto, H. Tada, Single-molecule rectifiers based on voltage-dependent deformation of molecular orbitals in carbazole oligomers, *Nanoscale* **2018**, *10*, 19818-19824. http://dx.doi.org/10.1039/C8NR06049E

10. ^{1}H and ^{13}C NMR Spectra of New Compounds

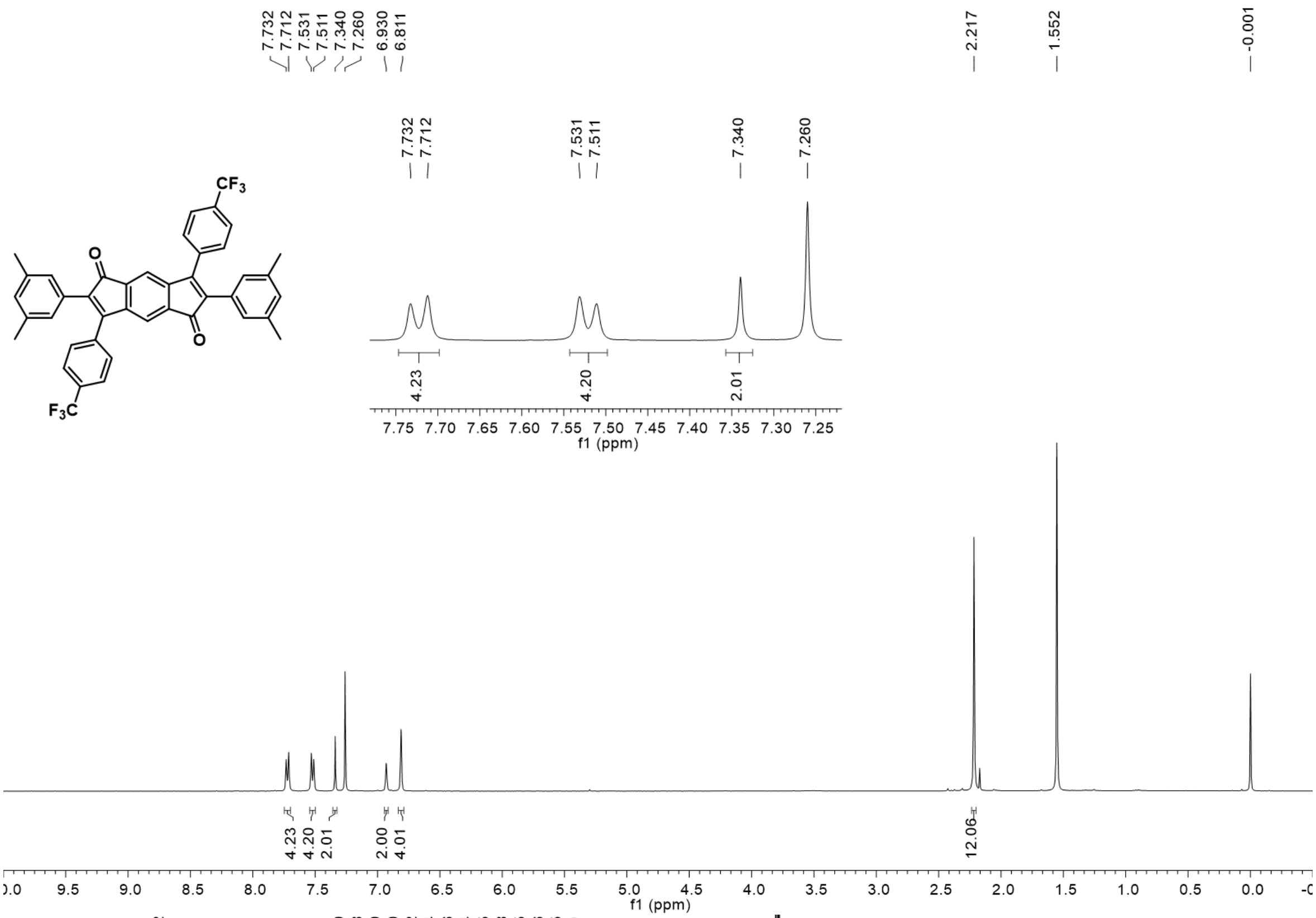

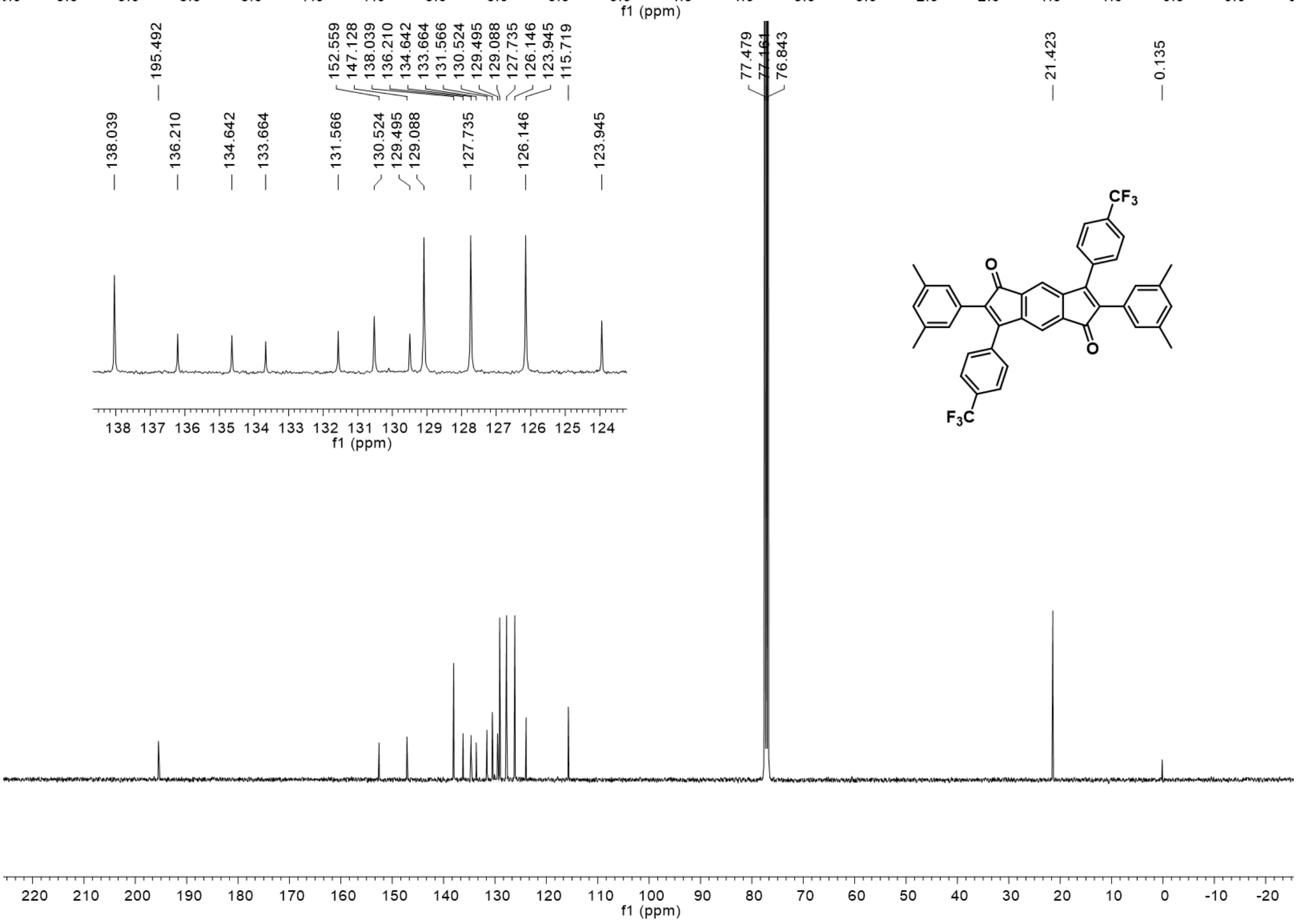

Figure S12. ^{1}H NMR (400 MHz, $CDCl_3$, 25 °C) and $^{13}C\{^{1}H\}\{^{19}F\}$ NMR (100 MHz, $CDCl_3$, 25 °C) spectra of compound **7b**.

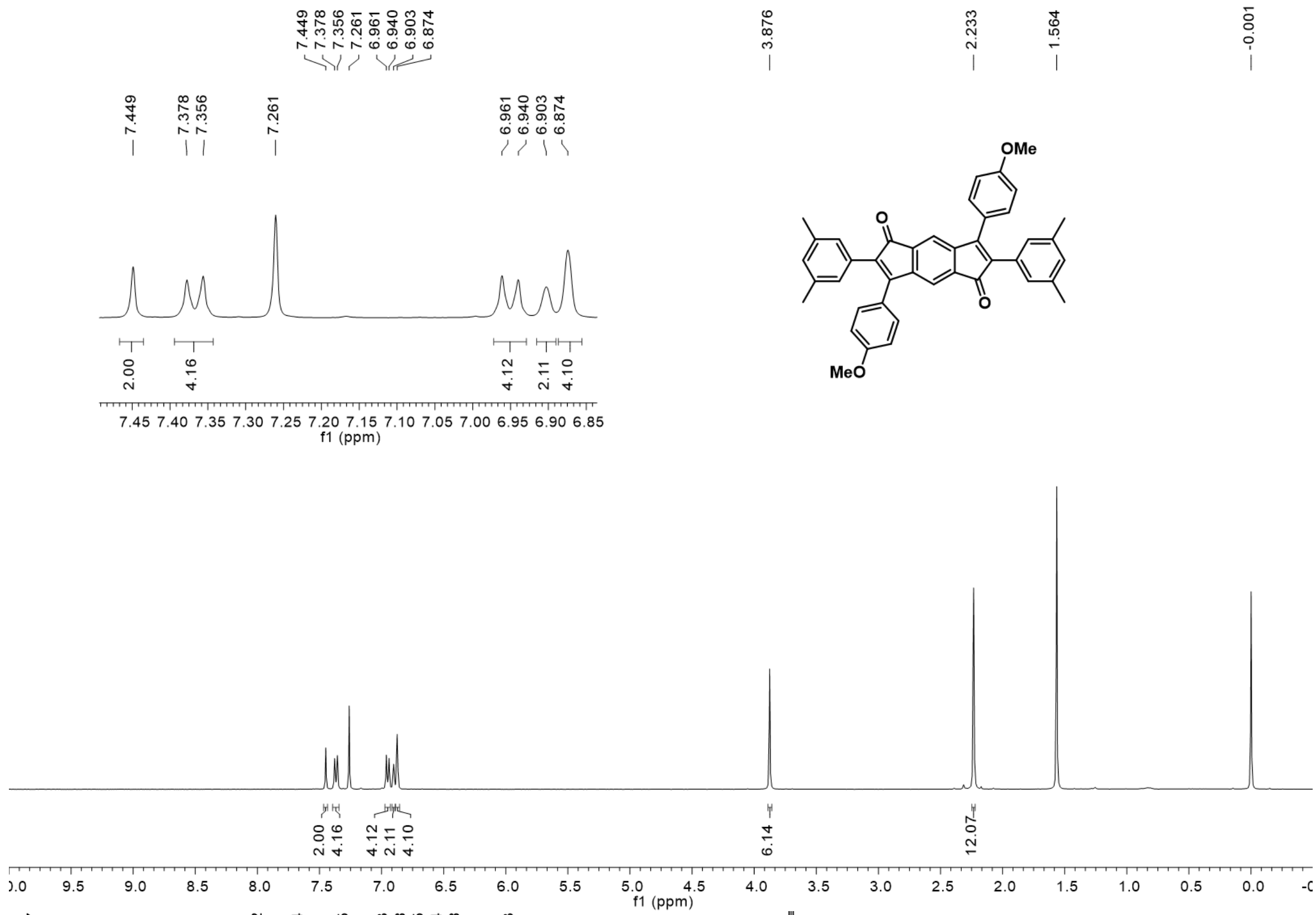

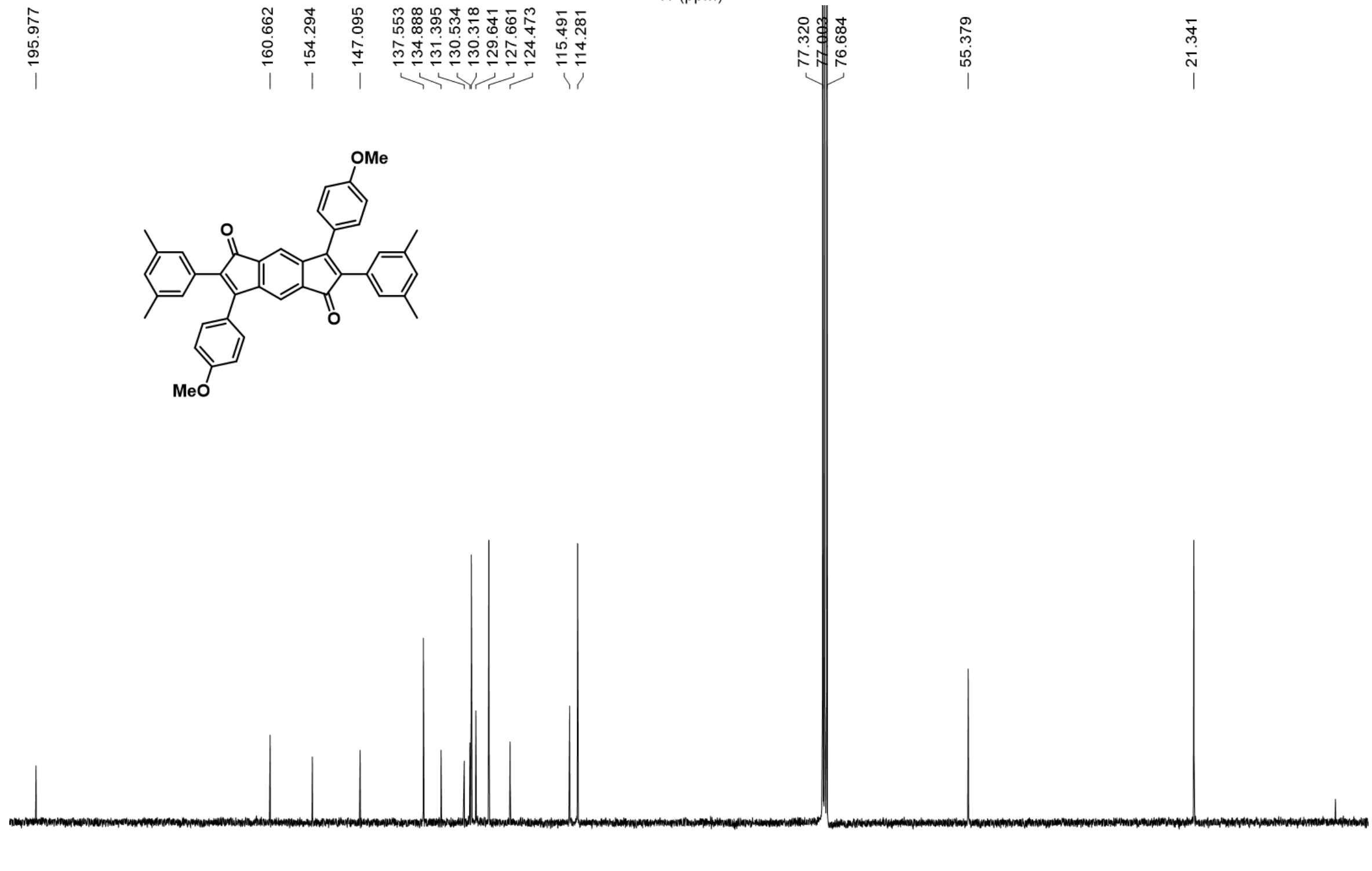

Figure S13. ^{1}H NMR (400 MHz, $CDCl_3$, 25 °C) and $^{13}C\{^{1}H\}$ NMR (100 MHz, $CDCl_3$, 25 °C) spectra of compound **7c**.

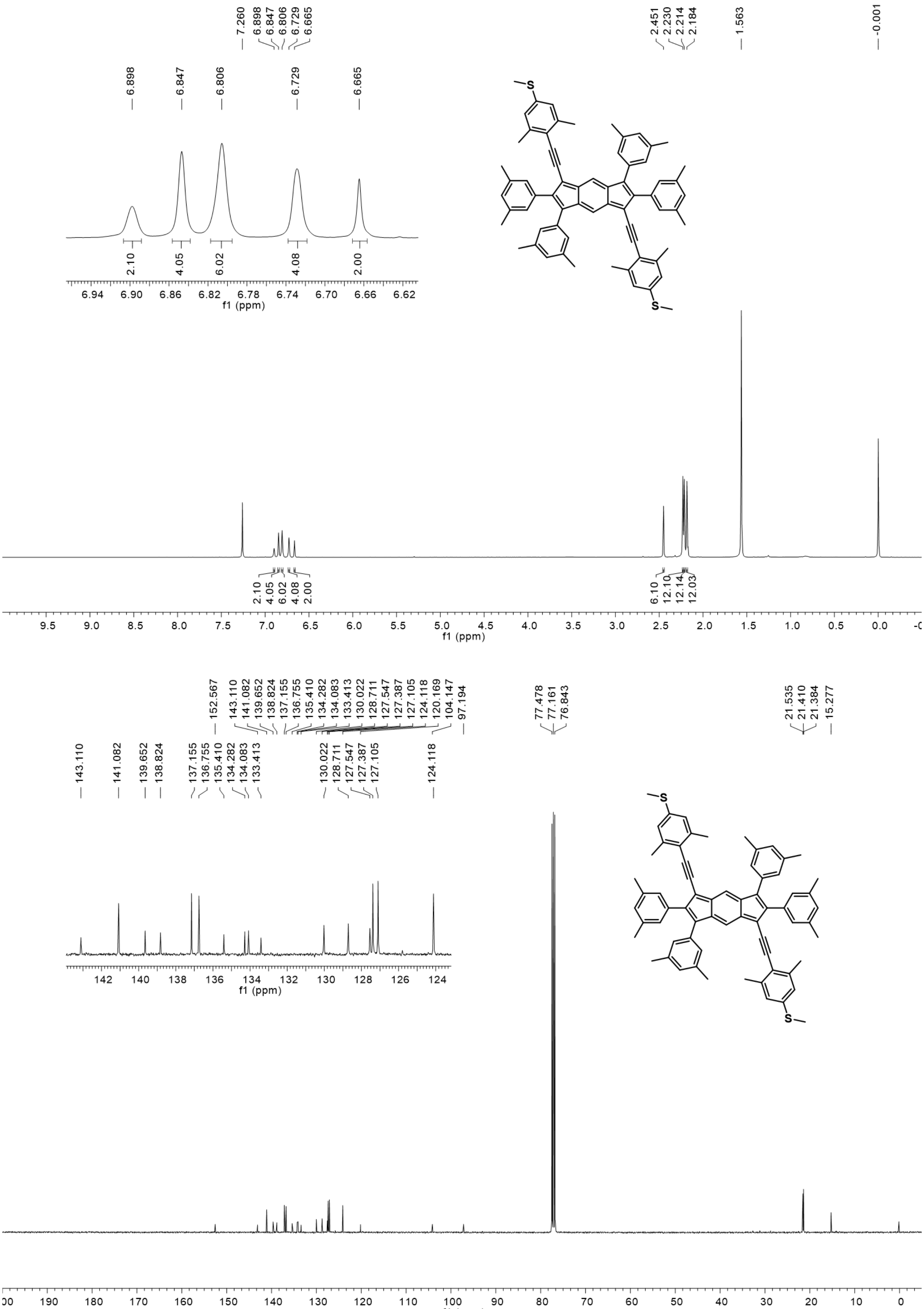

Figure S14. ^{1}H NMR (400 MHz, $CDCl_3$, 25 °C) and $^{13}C\{^{1}H\}$ NMR (100 MHz, $CDCl_3$, 25 °C) spectra of compound **15Ind-a**.

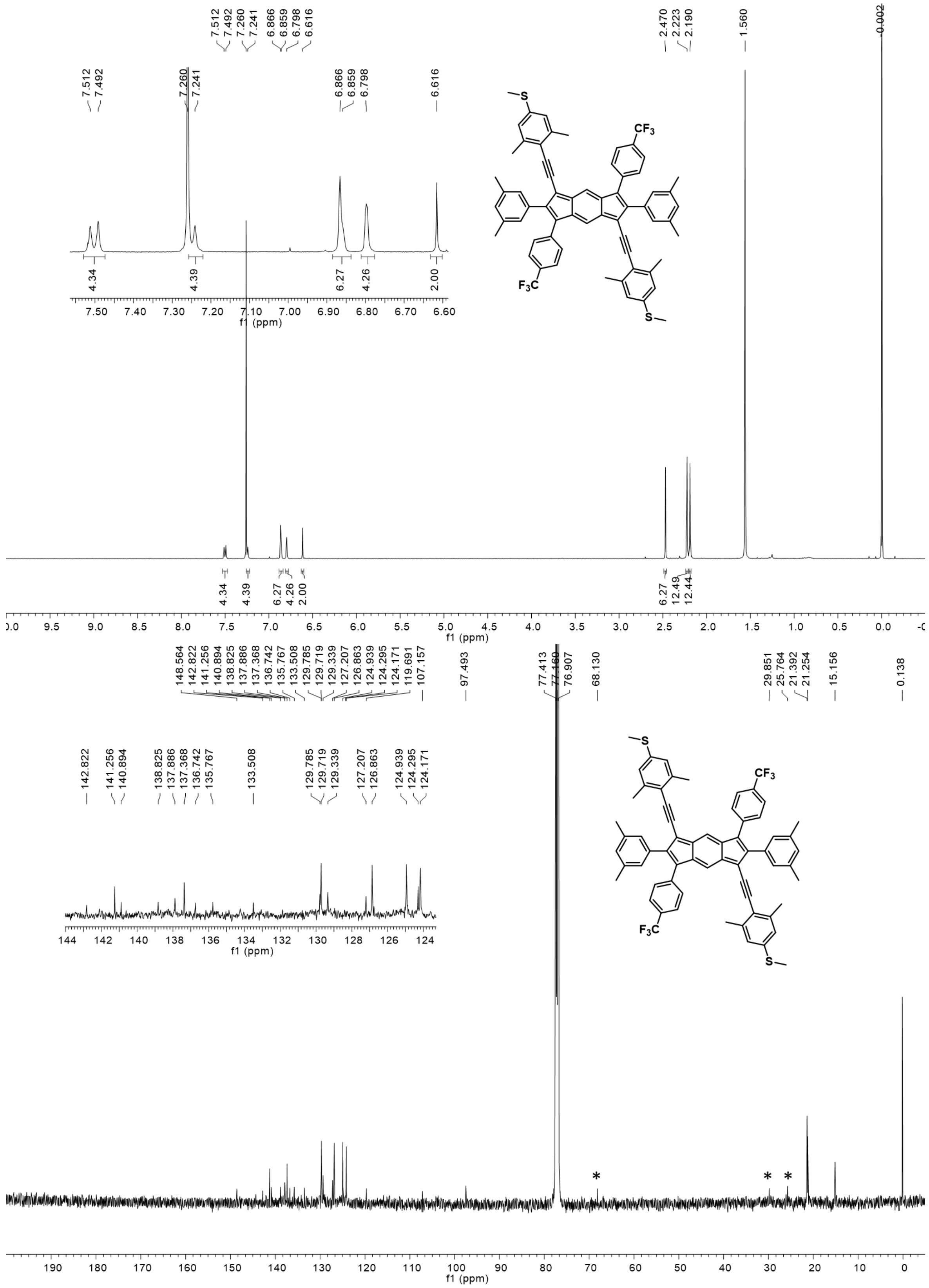

Figure S15. ^{1}H NMR (400 MHz, $CDCl_3$, 25 °C) and $^{13}C\{^{1}H\}\{^{19}F\}$ NMR (125 MHz, $CDCl_3$, 25 °C) spectra of compound **15Ind-b**. The asterisks indicate signals due to THF and grease in the solvent.

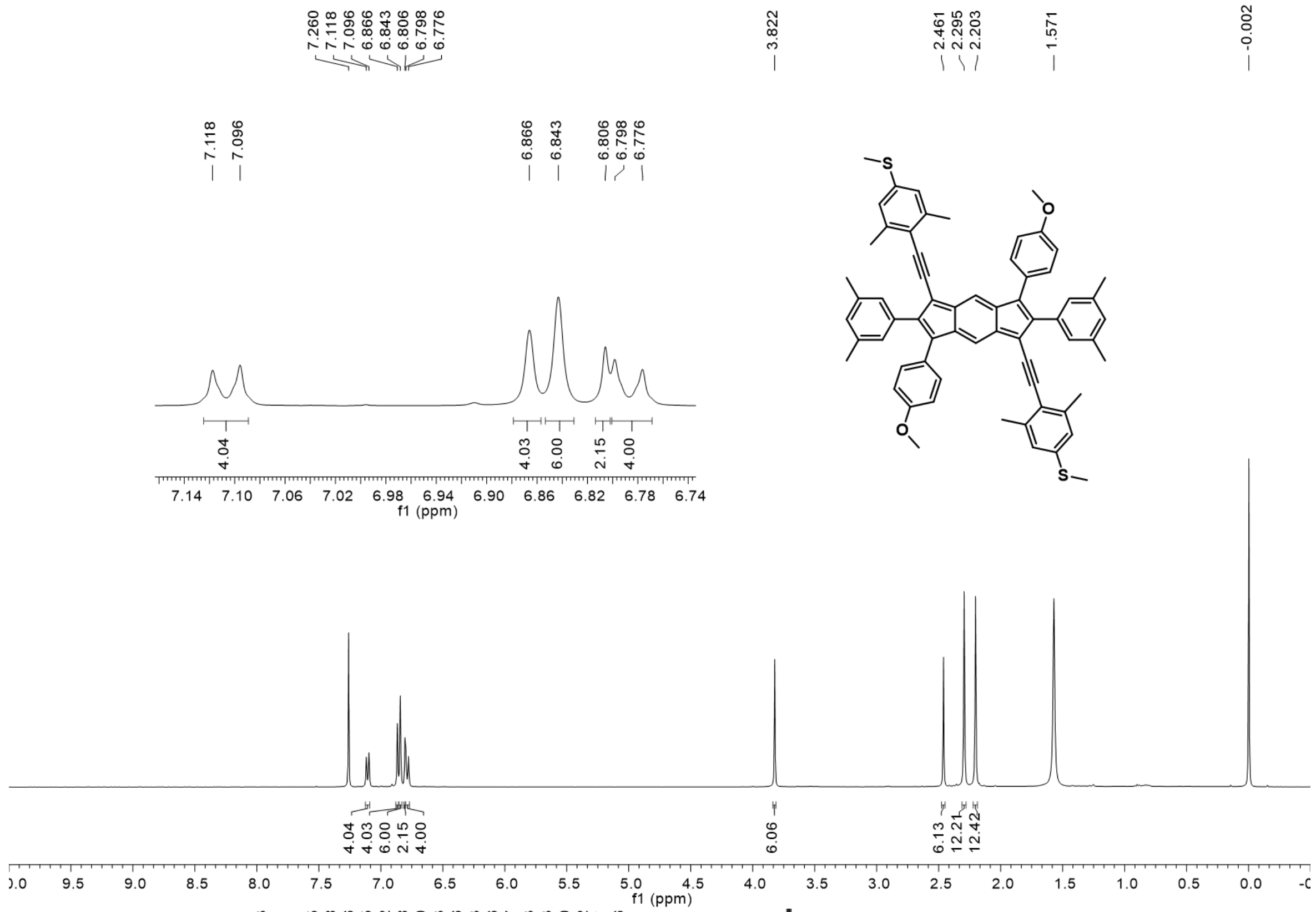

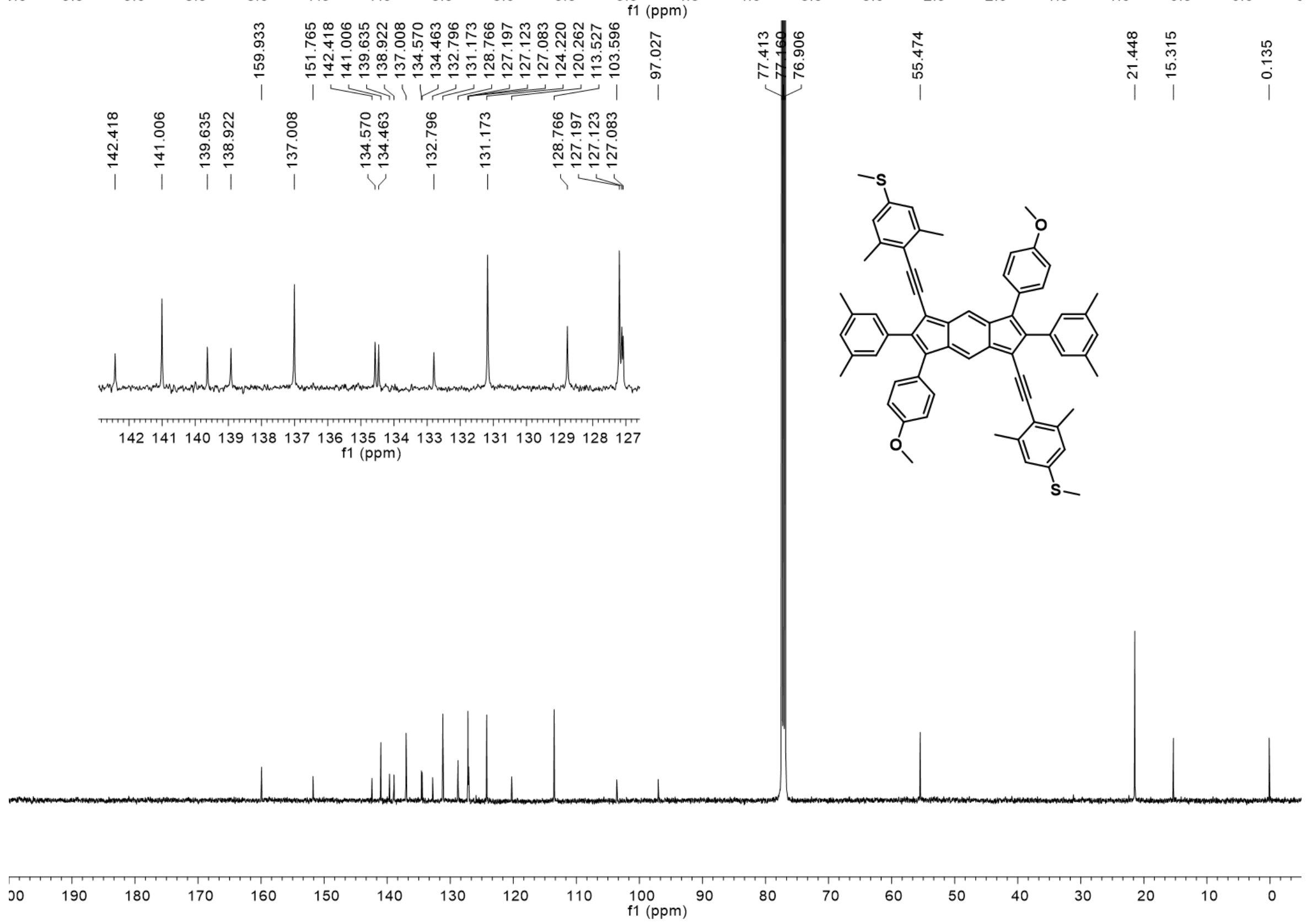

Figure S16. ^{1}H NMR (400 MHz, $CDCl_3$, 25 °C) and $^{13}C\{^{1}H\}$ NMR (125 MHz, $CDCl_3$, 25 °C) spectra of compound **15Ind-c**.

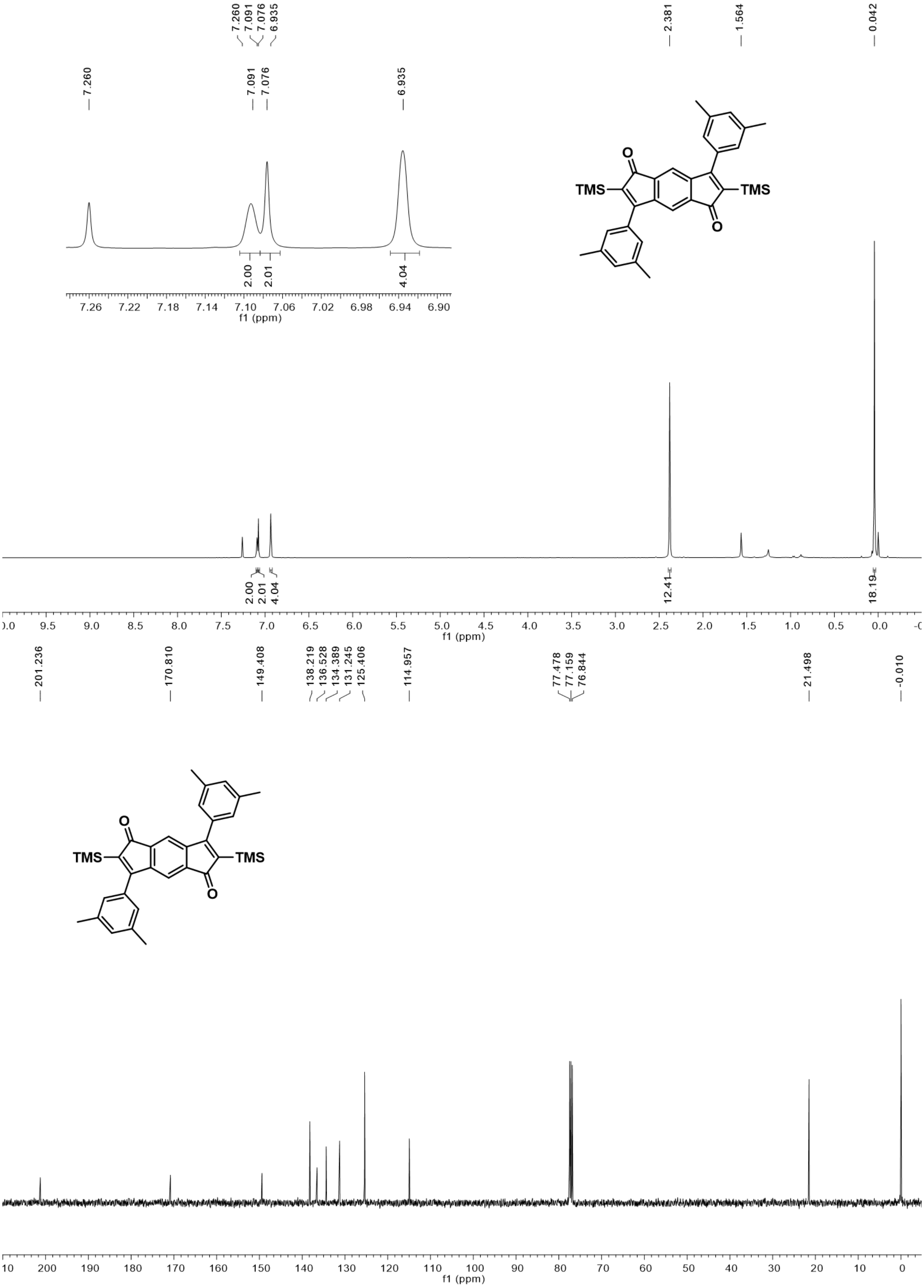

Figure S17. ^{1}H NMR (400 MHz, $CDCl_3$, 25 °C) and $^{13}C\{^{1}H\}$ NMR (100 MHz, $CDCl_3$, 25 °C) spectra of compound **1**.

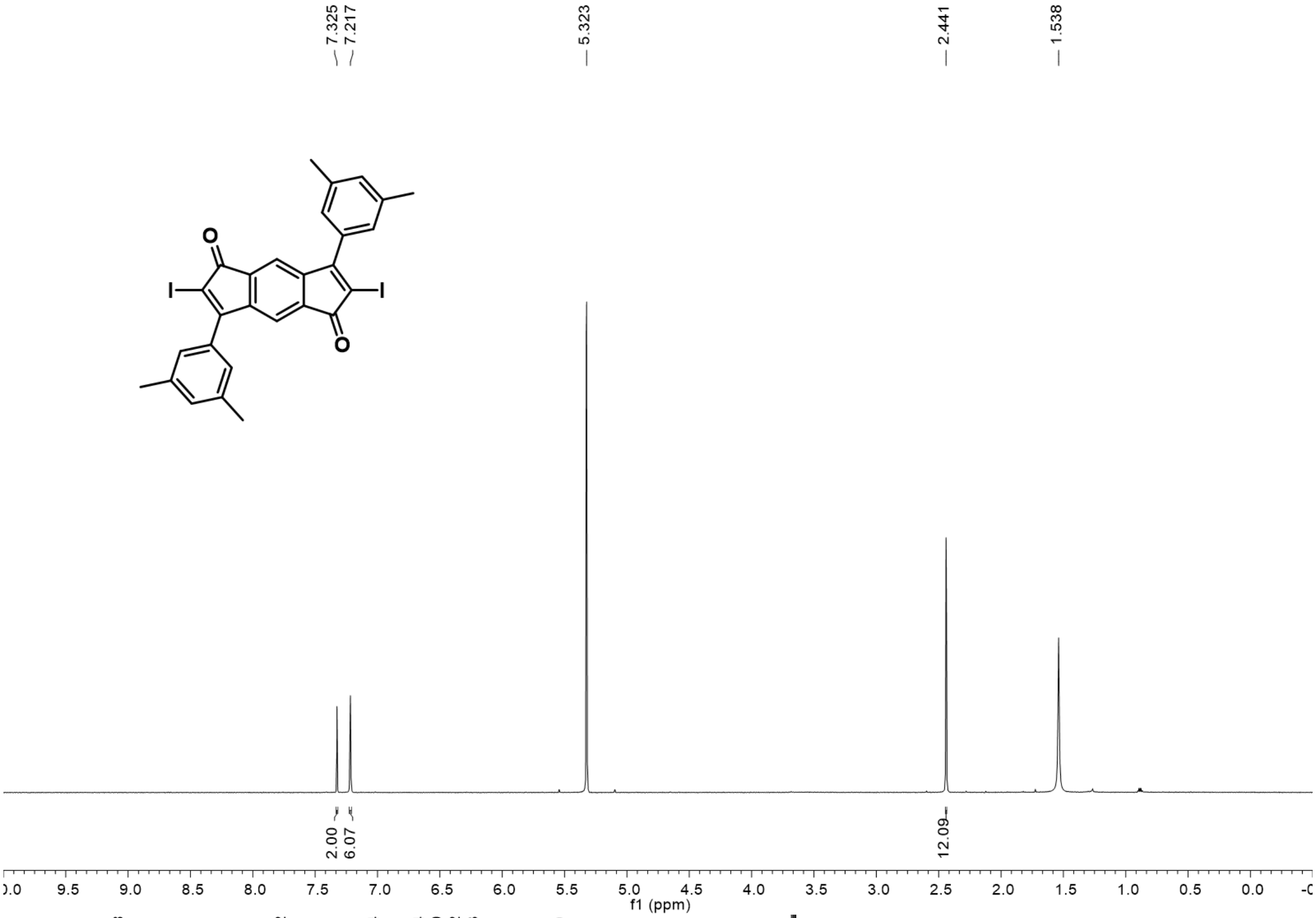

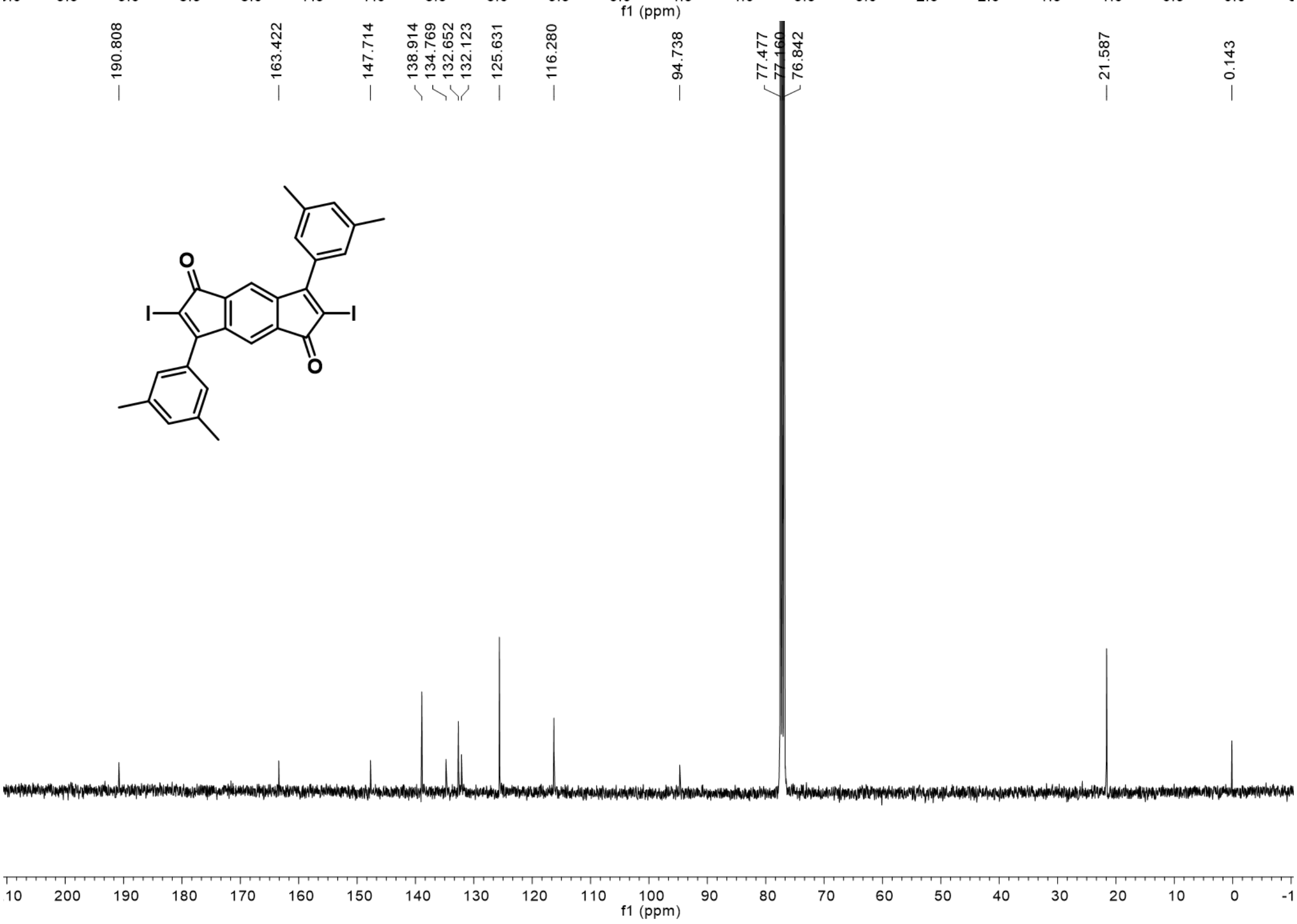

Figure S18. ^{1}H NMR (400 MHz, CD_2Cl_2, 25 °C) and $^{13}C\{^{1}H\}$ NMR (100 MHz, $CDCl_3$, 25 °C) spectra of compound **4**.

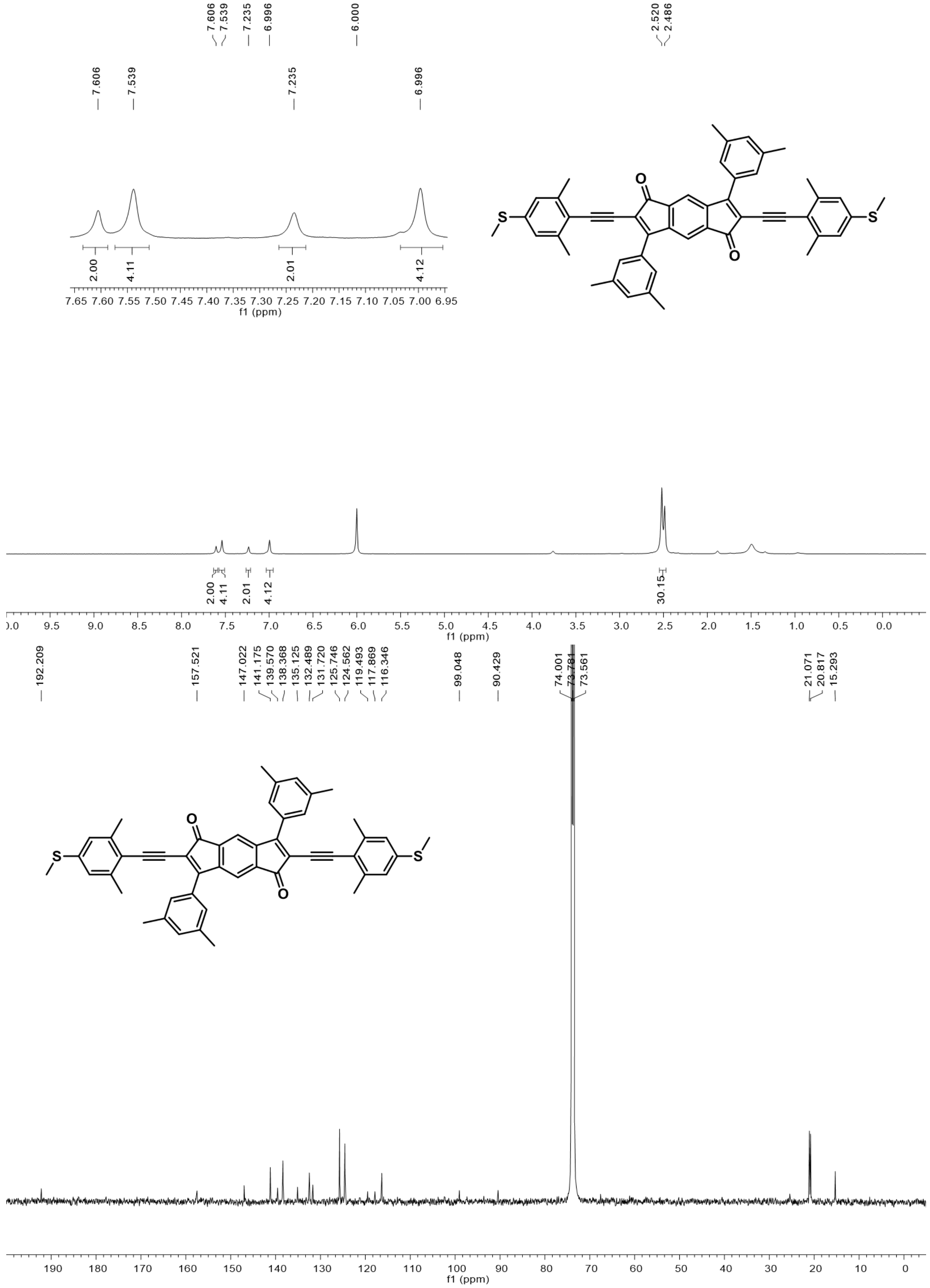

Figure S19. ^{1}H NMR (500 MHz, $C_2D_2Cl_4$, 100 °C) and $^{13}C\{^1H\}$ NMR (125 MHz, $C_2D_2Cl_4$, 100 °C) spectra of compound **2**.

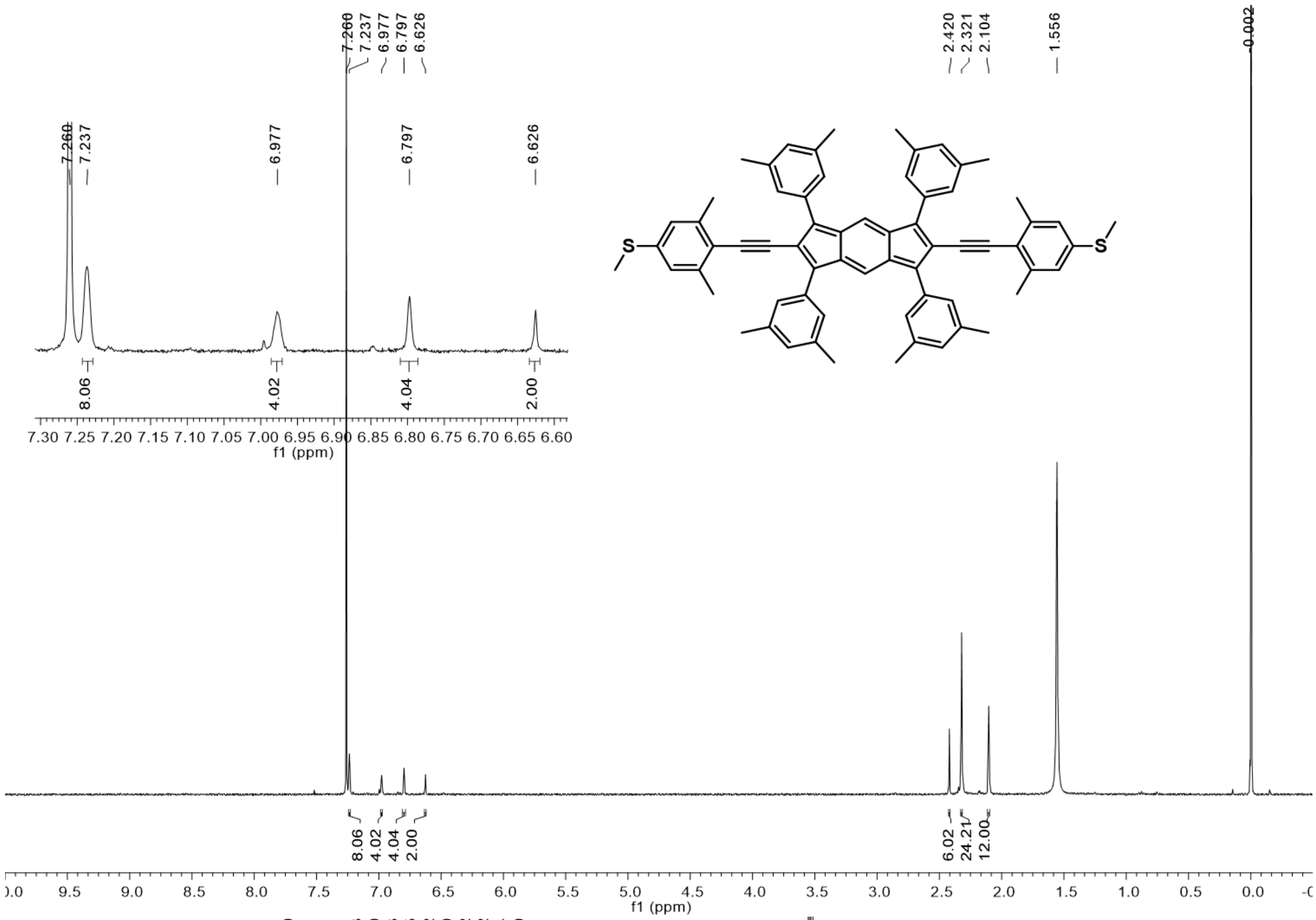

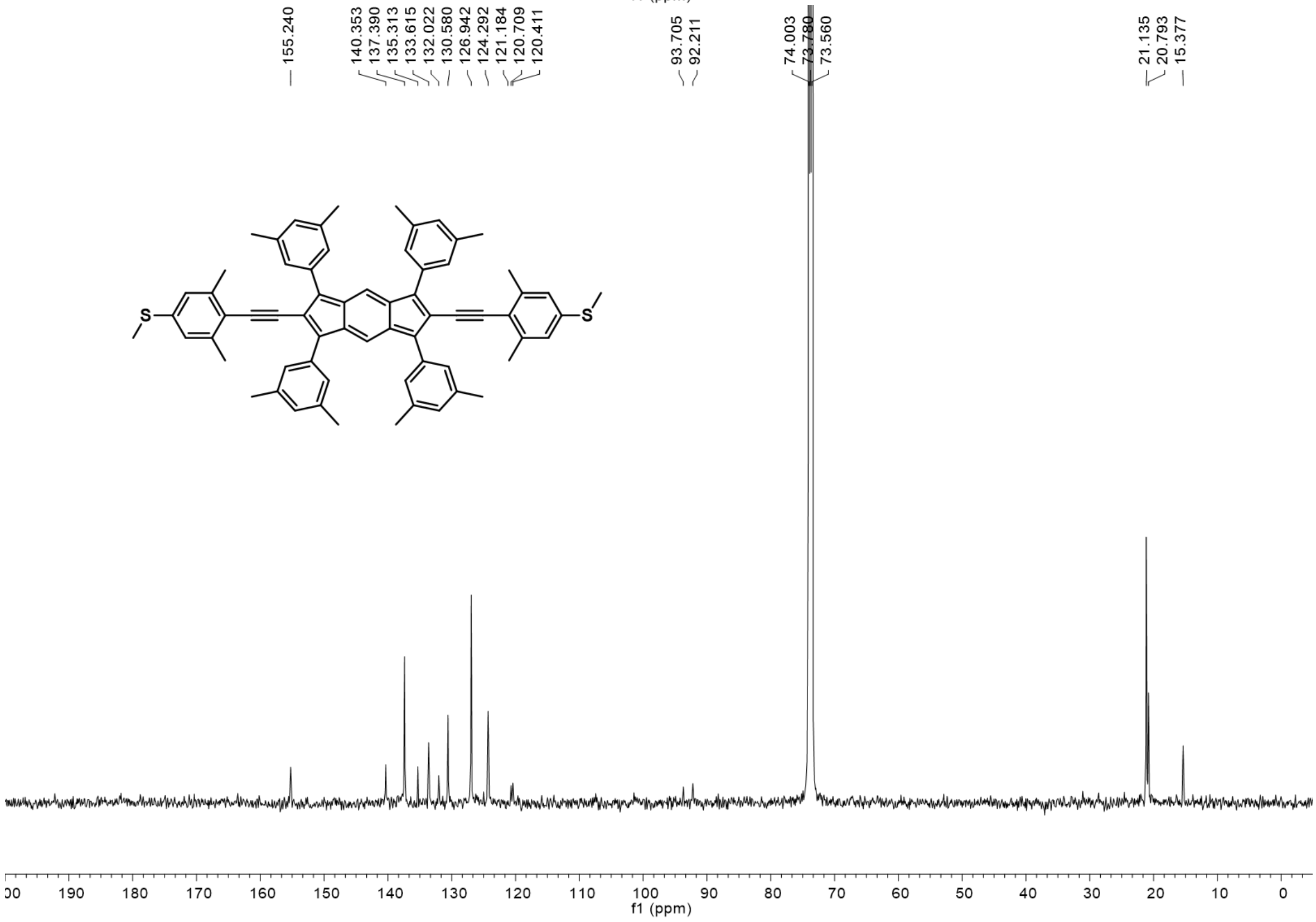

Figure S20. ^{1}H NMR (400 MHz, $CDCl_3$, 25 °C) and $^{13}C\{^{1}H\}$ NMR (125 MHz, $C_2D_2Cl_4$, 65 °C) spectra of compound **26Ind**.

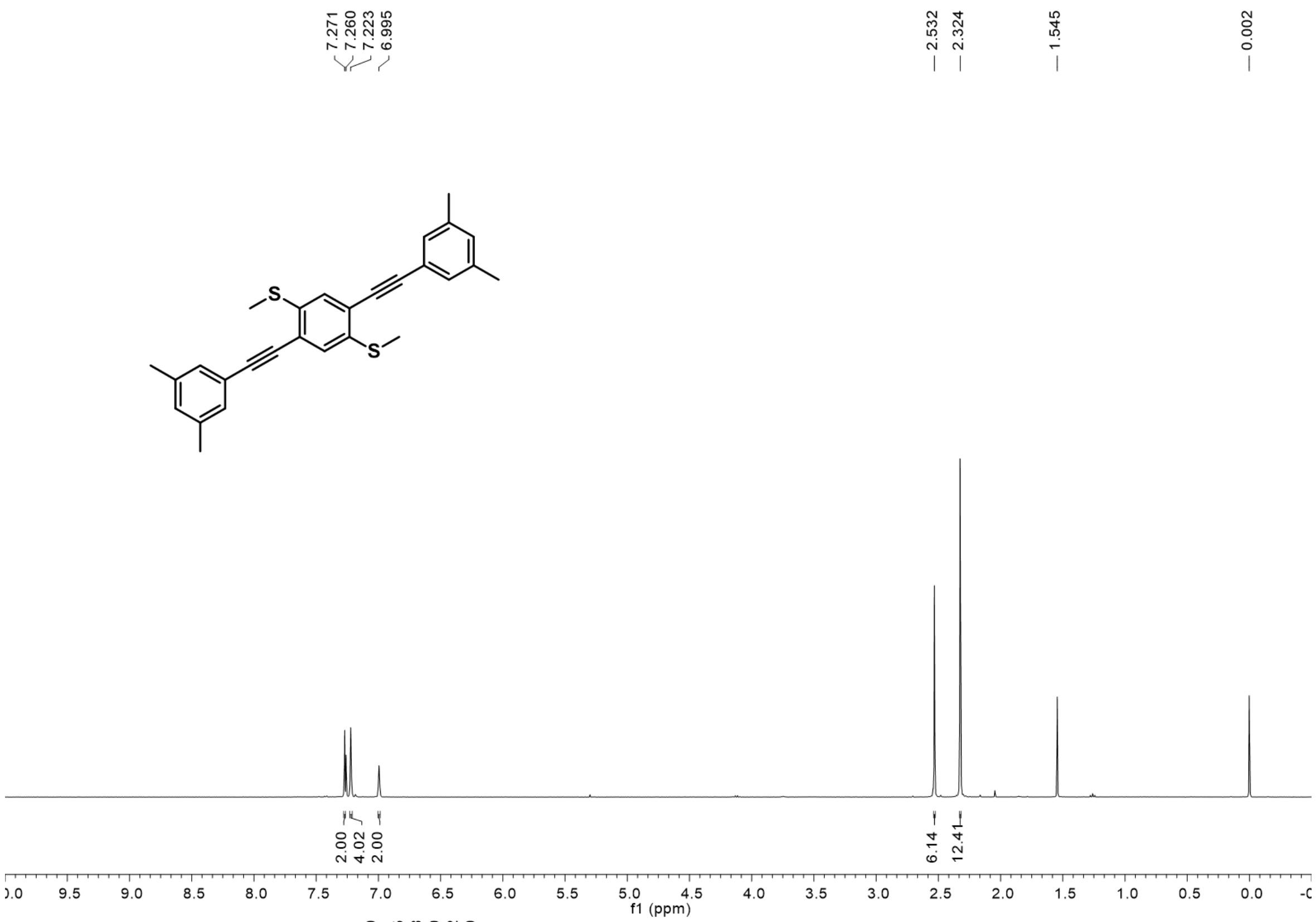

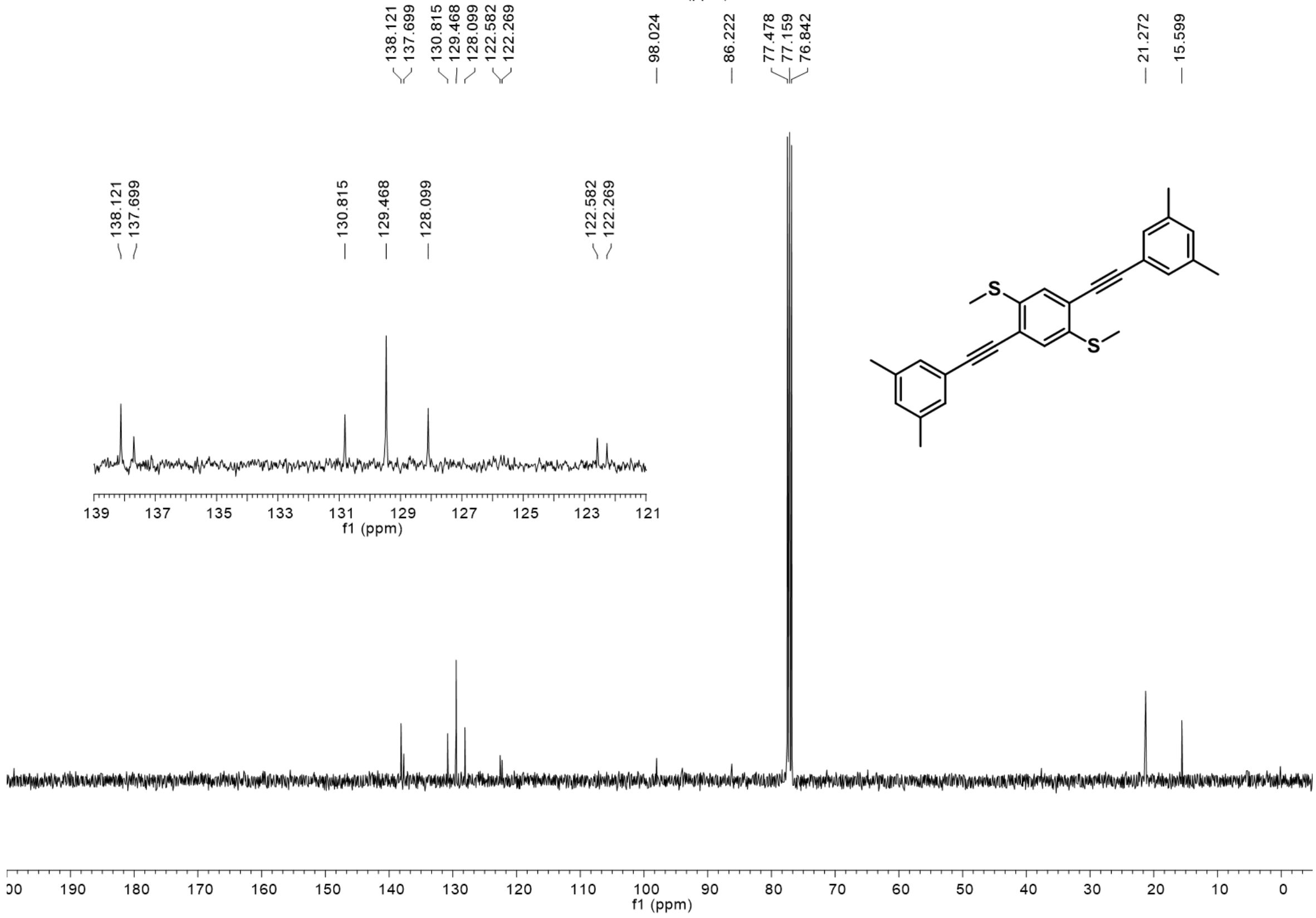

Figure S21. ^{1}H NMR (400 MHz, $CDCl_3$, 25 °C) and $^{13}C\{^{1}H\}$ NMR (100 MHz, $CDCl_3$, 25 °C) spectra of compound **8**.

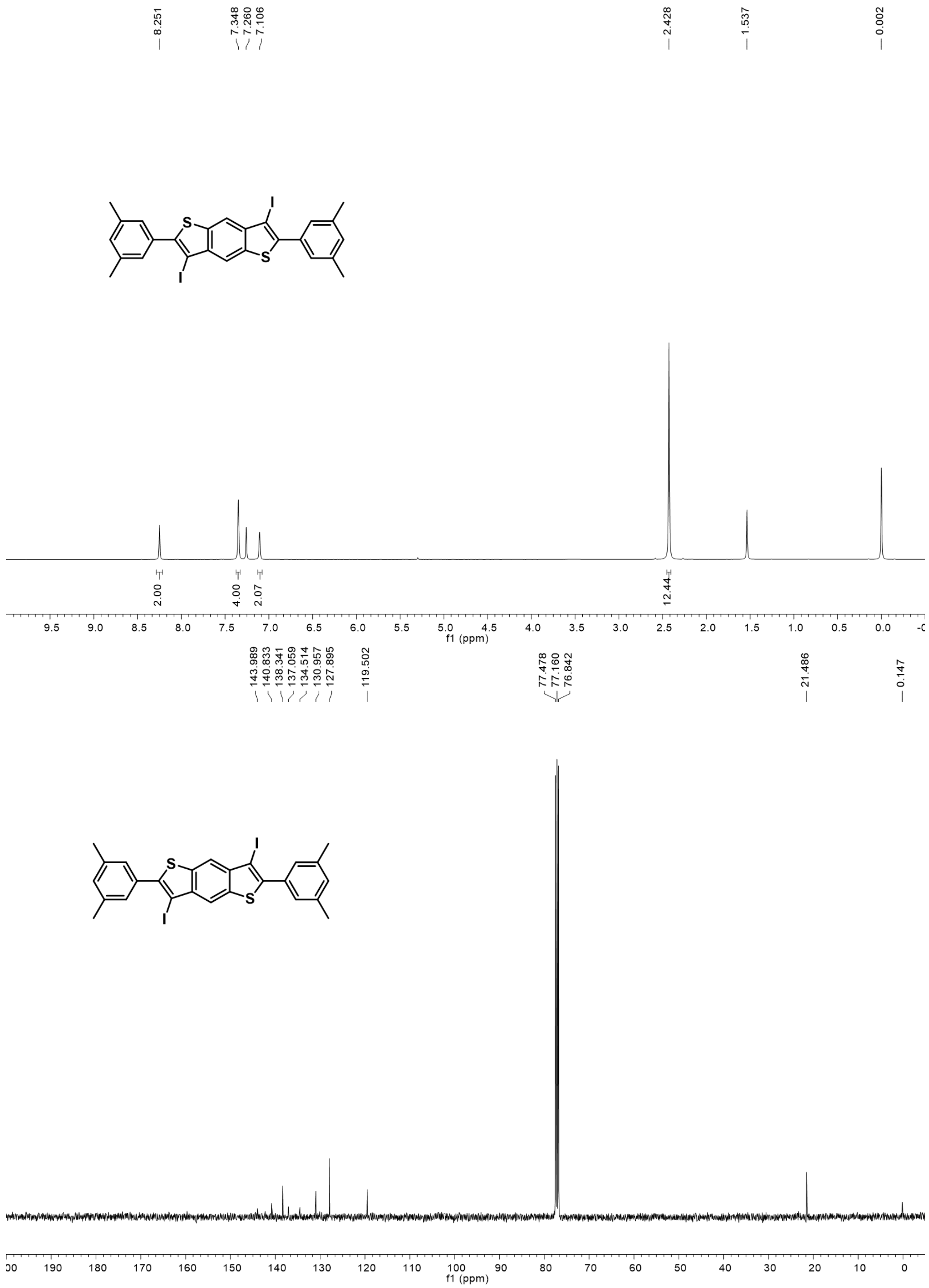

Figure S22. ^{1}H NMR (400 MHz, $CDCl_3$, 25 °C) and $^{13}C\{^{1}H\}$ NMR (100 MHz, $CDCl_3$, 25 °C) spectra of compound **9**.

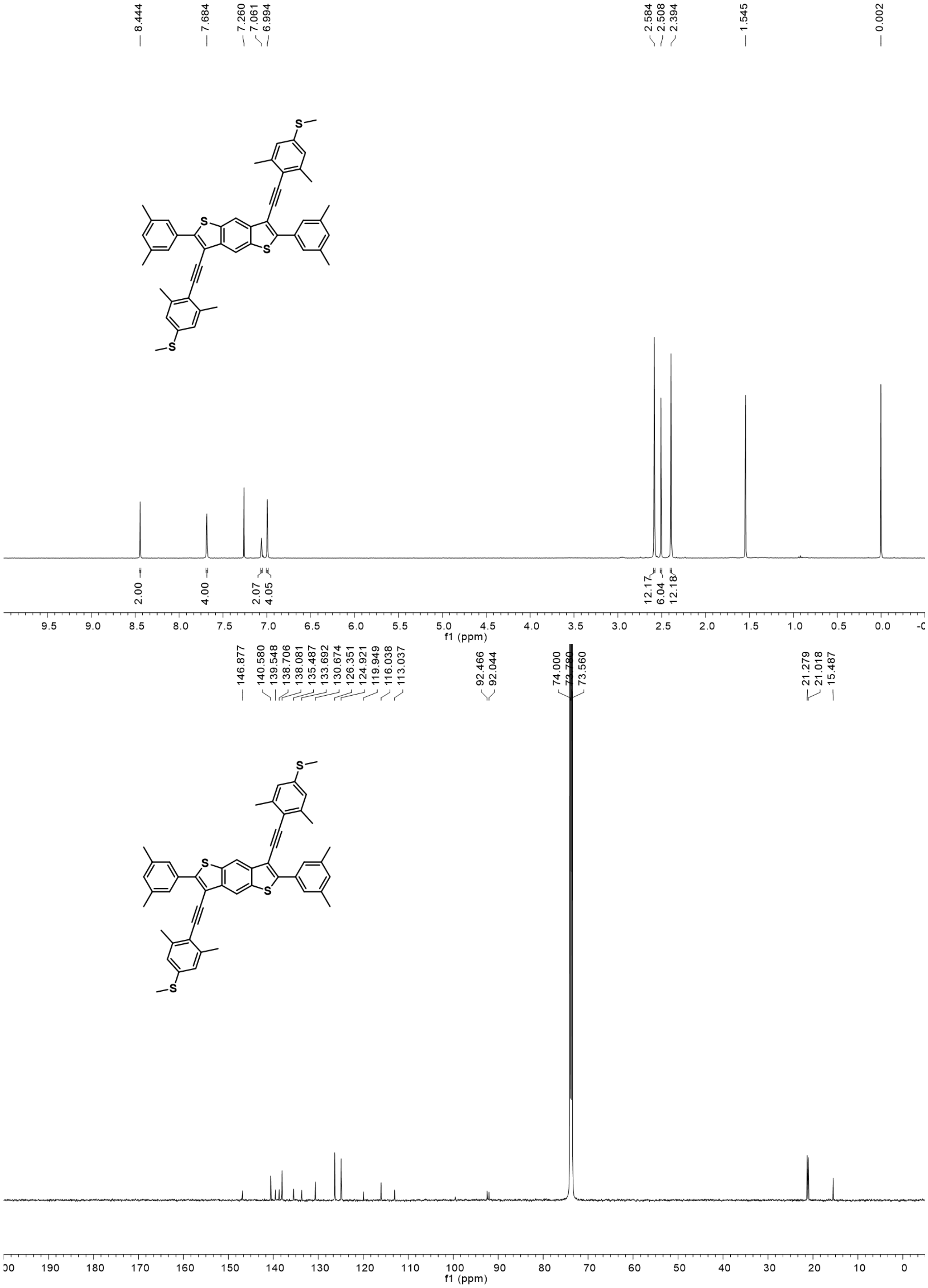

Figure S23. ^{1}H NMR (400 MHz, $CDCl_3$, 25 °C) and $^{13}C\{^{1}H\}$ NMR (125 MHz, $C_2D_2Cl_4$, 100 °C) spectra of compound **37BDT**.

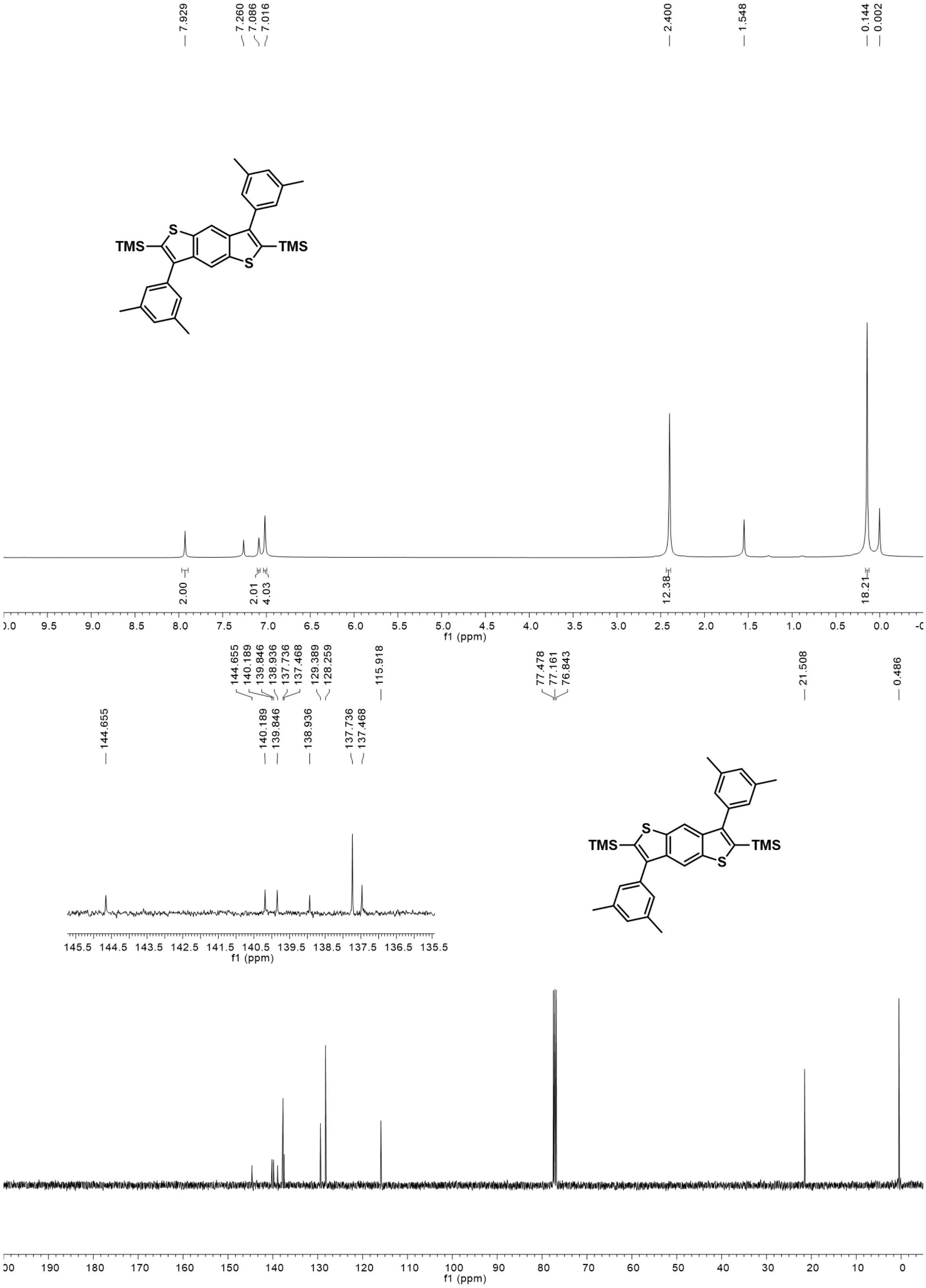

Figure S24. ^{1}H NMR (400 MHz, $CDCl_3$, 25 °C) and $^{13}C\{^{1}H\}$ NMR (100 MHz, $CDCl_3$, 25 °C) spectra of compound **11**.

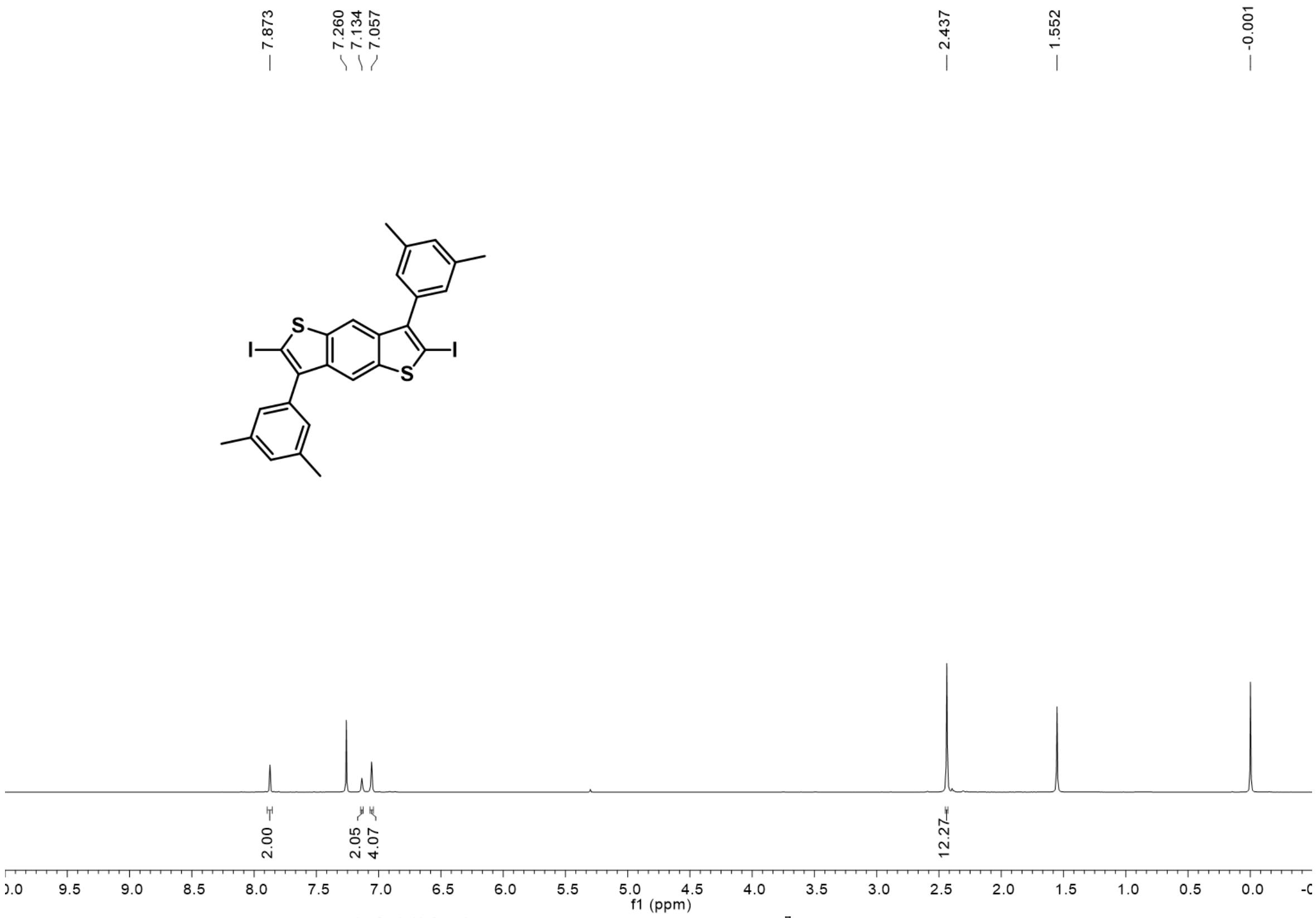

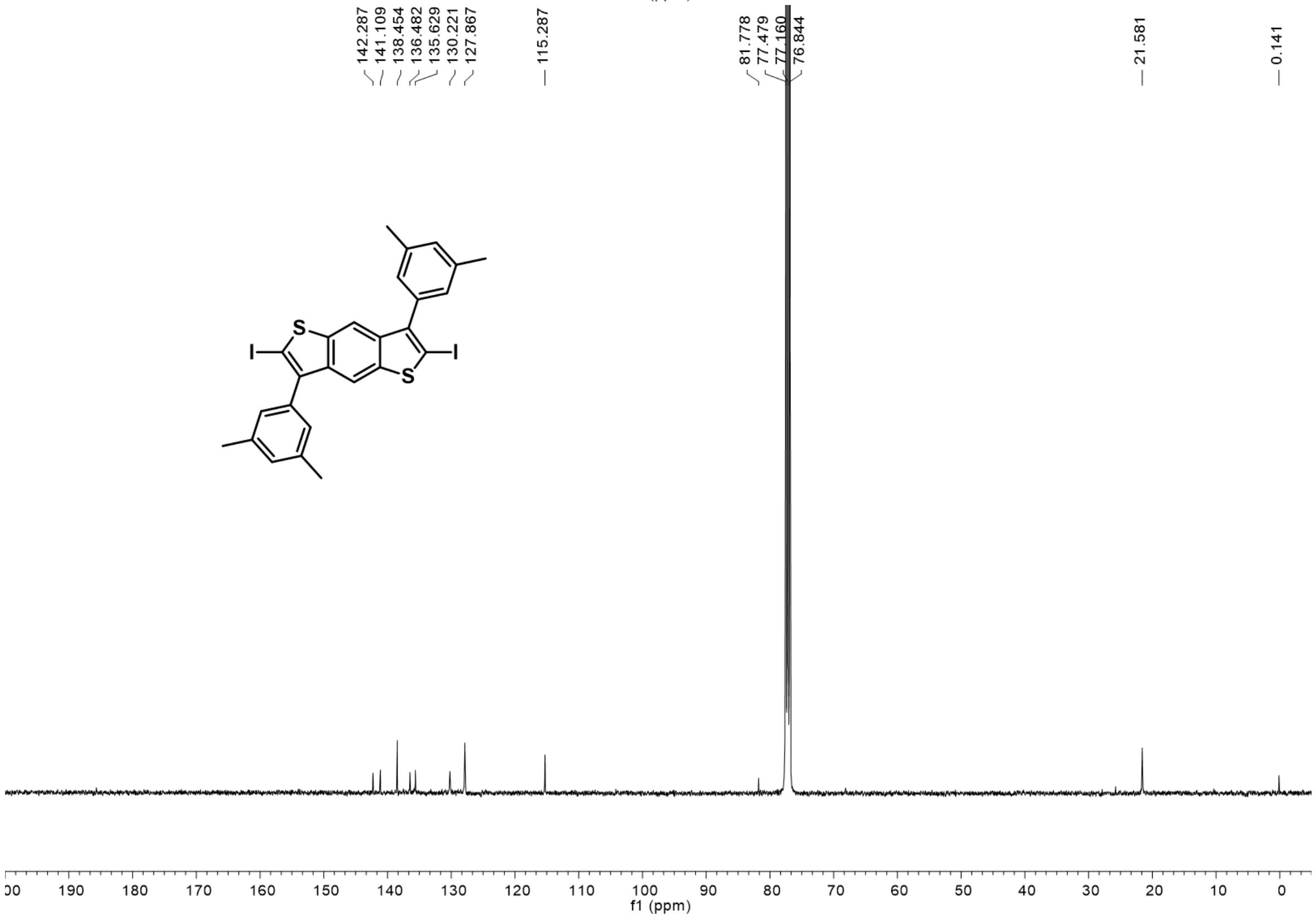

Figure S25. ^{1}H NMR (400 MHz, $CDCl_3$, 25 °C) and $^{13}C\{^{1}H\}$ NMR (100 MHz, $CDCl_3$, 25 °C) spectra of compound **12**.

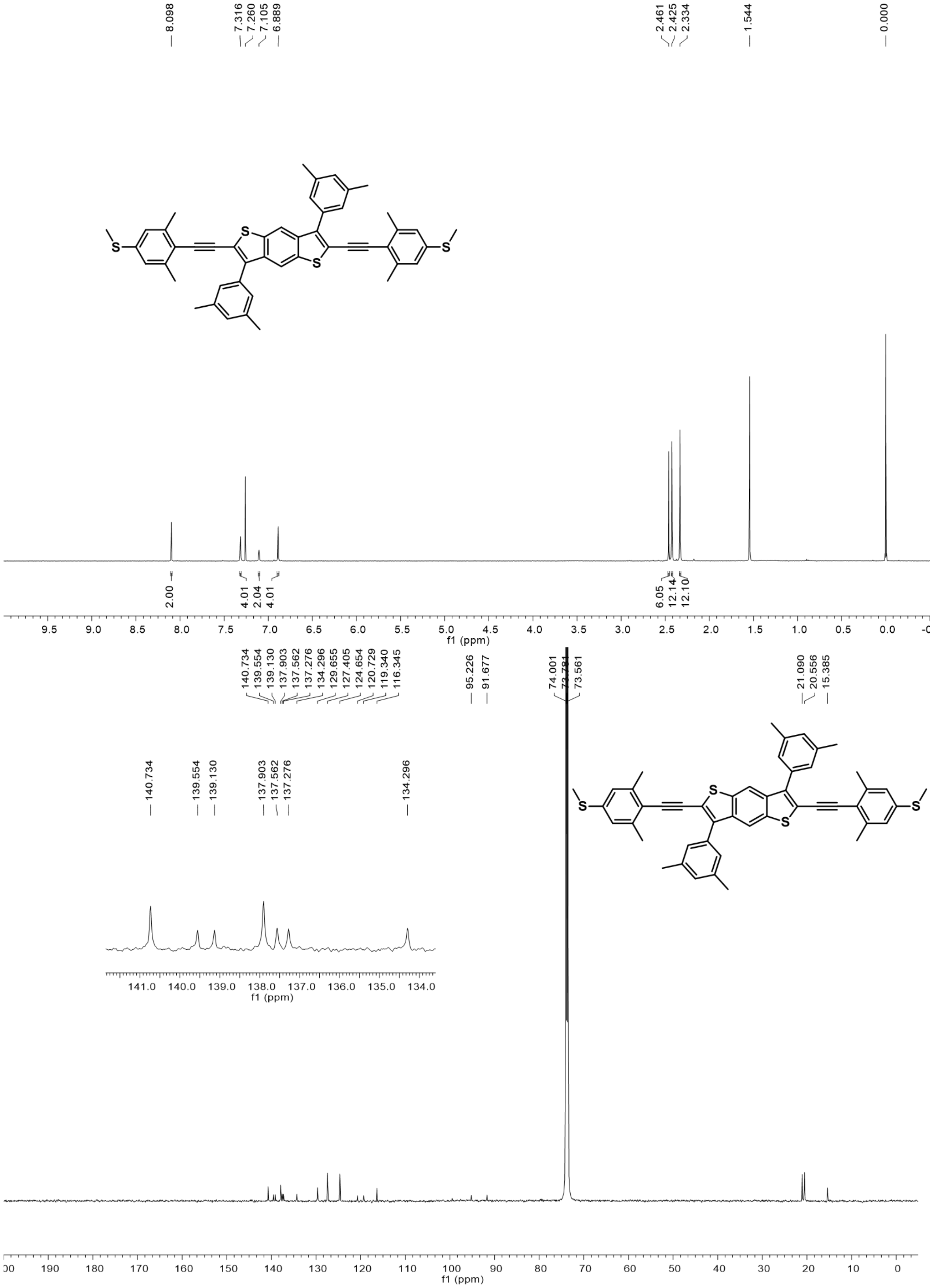

Figure S26. ^{1}H NMR (400 MHz, $CDCl_3$, 25 °C) and $^{13}C\{^{1}H\}$ NMR (125 MHz, $C_2D_2Cl_4$, 100 °C) spectra of compound **26BDT**.

11. Cartesian Coordinates for Optimized Geometries of 15Ind-a, 15Ind-b, 15Ind-c, 26Ind, 37BDT and 26BDT.

Cartesian Coordinates of 15Ind-a

C	1.02160800	1.00510300	-0.01063000	C	-5.41127500	4.41673900	1.14567900
C	2.61071700	-1.02208100	0.04868700	H	-4.37163900	2.67451300	1.84897200
C	2.37270000	-2.43471100	0.05134800	C	-5.43668200	5.41070900	0.16087900
C	0.97990000	-2.64894600	-0.01523900	H	-6.21481000	6.16852900	0.19153700
C	-1.02158800	-1.00512700	-0.01062500	C	-0.28088400	3.94253500	-0.02018800
C	-2.61069800	1.02205300	0.04869000	C	0.75826900	4.18611400	-0.93551200
C	-2.37268000	2.43468300	0.05137300	C	-0.62362800	4.95465400	0.88687700
C	-0.97988300	2.64892100	-0.01523500	C	1.43538500	5.40477500	-0.95697800
C	1.34210800	-0.35338500	-0.00612900	H	1.01030500	3.42306100	-1.66328600
C	0.32649300	-1.36439800	-0.04240000	C	0.05015800	6.17657400	0.89832000
C	-1.34208700	0.35336000	-0.00612300	H	-1.41886700	4.77652800	1.60104300
C	-0.32647400	1.36437300	-0.04239900	C	1.07204900	6.38760500	-0.03053600
H	1.80413600	1.75480600	0.02394400	H	1.59498500	7.34019200	-0.03560100
H	-1.80411600	-1.75483100	0.02395000	C	3.85388900	-0.38495700	0.04169400
C	3.43317700	-3.46491600	0.08216600	C	4.92390200	0.19708400	0.02449700
C	3.48752400	-4.47357500	-0.88501400	C	-3.85386900	0.38493200	0.04169200
C	4.40921100	-3.44870800	1.09029200	C	-4.92388200	-0.19711000	0.02447100
C	4.48520800	-5.45187100	-0.85951700	C	6.17523000	0.85625700	0.00399000
H	2.74285600	-4.48985300	-1.67332700	C	6.27187400	2.21656200	0.40360800
C	5.41135100	-4.41675700	1.14551400	C	7.33586800	0.16140200	-0.41659100
H	4.37177300	-2.67450900	1.84883100	C	7.50978600	2.84111500	0.38015000
C	5.43668700	-5.41075700	0.16073700	C	8.56109800	0.82400600	-0.42708500
H	6.21480300	-6.16859200	0.19137500	C	8.66328700	2.15954000	-0.03116900
C	0.28089100	-3.94255700	-0.02017100	H	7.58414800	3.87829400	0.68962800
C	-0.75824200	-4.18615600	-0.93550000	H	9.43585800	0.27672100	-0.75293700
C	0.62360300	-4.95464800	0.88694900	C	-6.17522700	-0.85624800	0.00397000
C	-1.43538200	-5.40481300	-0.95692700	C	-7.33582300	-0.16142000	-0.41677600
H	-1.01025600	-3.42312800	-1.66331000	C	-6.27193200	-2.21650200	0.40375200
C	-0.05021500	-6.17654000	0.89844600	C	-8.56106800	-0.82399300	-0.42725800
H	1.41882600	-4.77650000	1.60112600	C	-7.50985900	-2.84102500	0.38030000
C	-1.07209900	-6.38759500	-0.03042600	C	-8.66331800	-2.15947200	-0.03117000
H	-1.59506400	-7.34016700	-0.03544600	H	-9.43579400	-0.27673000	-0.75324100
C	-3.43315900	3.46488800	0.08223700	H	-7.58426300	-3.87816500	0.68989800
C	-3.48756500	4.47352900	-0.88496200	C	6.42857000	-4.41548900	2.26305000
C	-4.40912500	3.44869700	1.09042100	H	6.14733700	-5.12083200	3.05278500
C	-4.48525500	5.45181400	-0.85943000	H	7.41768800	-4.71047500	1.90289100
H	-2.74294400	4.48979100	-1.67332000	H	6.51537300	-3.42821300	2.72175200

C	4.53301300	-6.51854500	-1.92848200
H	5.27539800	-7.28430700	-1.69437700
H	3.56272300	-7.01106100	-2.03931500
H	4.79177400	-6.09077500	-2.90265200
C	0.30287800	-7.23639100	1.91538300
H	0.03886200	-8.23460000	1.55865300
H	1.37131800	-7.22780700	2.14286000
H	-0.23337900	-7.07138000	2.85632400
C	-2.51982700	-5.67386800	-1.97406300
H	-3.41688800	-6.08308500	-1.50077300
H	-2.80437700	-4.76274600	-2.50387100
H	-2.18727400	-6.40204500	-2.72121700
C	-6.42840100	4.41548200	2.26330000
H	-6.14656900	5.12008200	3.05348500
H	-7.41732800	4.71143800	1.90342300
H	-6.51589200	3.42796600	2.72136200
C	-4.53311200	6.51848600	-1.92839500
H	-5.27580900	7.28400300	-1.69448500
H	-3.56295000	7.01132300	-2.03894500
H	-4.79145400	6.09064900	-2.90264500
C	-0.30303200	7.23646600	1.91517900
H	-0.03818200	8.23457700	1.55879900
H	-1.37164200	7.22846900	2.14189700
H	0.23244600	7.07098000	2.85647900
C	2.51993300	5.67377800	-1.97401800
H	3.41741800	6.08178200	-1.50047100
H	2.80362300	4.76287100	-2.50465100
H	2.18794300	6.40292000	-2.72047600
S	10.17980800	3.08783500	-0.00369800
S	-10.17986700	-3.08771800	-0.00366100
C	11.42296800	1.91286700	-0.62641200
H	12.36351500	2.46413000	-0.62925700
H	11.52219900	1.04892400	0.03131000
H	11.19617400	1.59514700	-1.64447700
C	-11.42294800	-1.91281300	-0.62664800
H	-12.36351200	-2.46404700	-0.62946700
H	-11.52219600	-1.04876100	0.03092800
H	-11.19607500	-1.59526400	-1.64474900
C	7.24859200	-1.27197700	-0.87165800
H	6.76985300	-1.90413200	-0.12032700
H	6.64108100	-1.35716000	-1.77799000
H	8.23961400	-1.67588000	-1.08609400
C	5.04800500	2.97066000	0.85355400
H	4.30570200	3.03238400	0.05210100
H	4.56200200	2.46917100	1.69509700
H	5.30331800	3.98631000	1.15965400
C	-5.04811200	-2.97057400	0.85387400
H	-4.30572300	-3.03235100	0.05250800
H	-4.56220100	-2.46903400	1.69544000
H	-5.30346200	-3.98620400	1.16001400
C	-7.24848000	1.27188900	-0.87205200
H	-6.76976900	1.90414800	-0.12079300
H	-6.64091500	1.35691500	-1.77836300
H	-8.23947800	1.67578600	-1.08660900

Cartesian Coordinates of 15Ind-b

C	-1.21779100	-0.75515200	-0.04890700
C	-2.31305000	1.57447600	0.02144000
C	-1.76238500	2.90753100	0.03250900
C	-0.36693800	2.80480600	-0.04338900
C	1.21785400	0.75519500	-0.04887100
C	2.31310200	-1.57446100	0.02143900
C	1.76242800	-2.90750400	0.03247000
C	0.36697100	-2.80476500	-0.04336900
C	-1.22849400	0.64522600	-0.04402000
C	-0.01234000	1.40191400	-0.08073300
C	1.22856000	-0.64518600	-0.04399700
C	0.01240200	-1.40187000	-0.08075600
H	-2.14889900	-1.30893100	-0.00969900
H	2.14896300	1.30897900	-0.00969600
C	-2.56142500	4.15060200	0.07883700
C	-2.40434400	5.14108900	-0.89588800
C	-3.49233600	4.36021900	1.10739200
C	-3.14944000	6.32302800	-0.85721500
H	-1.69936100	4.98034600	-1.70444700
C	-4.24545100	5.53186200	1.17409200
H	-3.61845800	3.59857700	1.86915700
C	-4.06455800	6.50121200	0.18101300
H	-4.65185000	7.41455300	0.21860000
C	0.61631100	3.89730100	-0.03946600
C	1.68074800	3.89863600	-0.95932400
C	0.54701900	4.94341300	0.89423600

C	2.64481500	4.89693700	-0.93669800	S	-10.51785000	-1.01450800	-0.11421200
H	1.73297700	3.12111100	-1.71142900	S	10.51789000	1.01453000	-0.11439100
C	1.51727900	5.93715200	0.92741000	C	-11.54713500	0.44101600	-0.48320900
H	-0.26432700	4.96357500	1.61041800	H	-11.30337000	0.86464200	-1.45781000
C	2.57149300	5.91508100	0.01481600	H	-12.57182400	0.06956900	-0.50538400
C	2.56143200	-4.15060200	0.07878300	H	-11.46282800	1.19847300	0.29646600
C	2.40438400	-5.14103200	-0.89604600	C	11.54722000	-0.44101900	-0.48325200
C	3.49226900	-4.36030900	1.10734700	H	12.57189800	-0.06954100	-0.50534900
C	3.14937800	-6.32300100	-0.85737700	H	11.46282700	-1.19838500	0.29648800
H	1.69949700	-4.98019500	-1.70466300	H	11.30354600	-0.86471600	-1.45784200
C	4.24535200	-5.53200600	1.17401400	C	-5.22023200	5.76627000	2.30463900
H	3.61842600	-3.59869600	1.86913600	H	-5.42729500	4.84388400	2.85098900
C	4.06446300	-6.50128300	0.18090400	H	-4.82389700	6.49294800	3.02186400
C	-0.61629600	-3.89723900	-0.03941800	H	-6.17037100	6.16257500	1.93577600
C	-1.68076800	-3.89853700	-0.95924500	C	-2.95009500	7.38798100	-1.90982900
C	-0.54701500	-4.94336000	0.89427300	H	-2.04517300	7.97186400	-1.71010200
C	-2.64485800	-4.89680500	-0.93660400	H	-2.83896200	6.94891500	-2.90459100
H	-1.73298600	-3.12101300	-1.71135200	H	-3.79166000	8.08335300	-1.93835100
C	-1.51731400	-5.93706600	0.92747100	C	5.22013000	-5.76647400	2.30455400
H	0.26433600	-4.96355000	1.61044600	H	5.42681200	-4.84422000	2.85126800
C	-2.57154800	-5.91495700	0.01490900	H	4.82400500	-6.49357100	3.02147000
C	-3.66305300	1.22412100	0.01843300	H	6.17044500	-6.16229400	1.93561900
C	3.66311500	-1.22413900	0.01846500	H	4.65175600	-7.41463400	0.21844900
C	-4.82756400	0.86381600	-0.00257200	C	2.94981000	-7.38820500	-1.90969300
C	4.82763300	-0.86386000	-0.00250700	H	3.79512000	-8.07879000	-1.94381900
C	-6.17752800	0.44946800	-0.03318600	H	2.04949300	-7.97746500	-1.70490900
C	-6.50854300	-0.91756500	0.18058900	H	2.83013100	-6.94902200	-2.90337300
C	-7.20506200	1.39302200	-0.28495600	C	-5.43074600	-1.93615300	0.44422900
C	-7.83927100	-1.30274900	0.14472700	H	-4.72640100	-1.98547300	-0.39144700
C	-8.52975300	0.96605600	-0.31364600	H	-4.84990900	-1.67552000	1.33359300
C	-8.86153800	-0.37459500	-0.10009300	H	-5.85315200	-2.93114600	0.59086800
H	-8.09200700	-2.34456100	0.31041100	C	-6.87116300	2.83939400	-0.53948400
H	-9.30066400	1.69978200	-0.50879200	H	-6.28644500	3.26542300	0.27919600
C	6.17760000	-0.44952000	-0.03308400	H	-6.26156500	2.94809500	-1.44149800
C	7.20513300	-1.39307800	-0.28484600	H	-7.77713600	3.43467900	-0.66536700
C	6.50861600	0.91750400	0.18071700	C	6.87124100	-2.83947200	-0.53925700
C	8.52981900	-0.96610300	-0.31361500	H	6.28699500	-3.26557700	0.27972400
C	7.83934700	1.30268800	0.14484600	H	6.26117700	-2.94821700	-1.44094600
C	8.86160200	0.37454800	-0.10006500	H	7.77720500	-3.43466900	-0.66561600
H	9.30071500	-1.69981900	-0.50885600	C	5.43081500	1.93610000	0.44431300
H	8.09209700	2.34446900	0.31070000	H	4.72718300	1.98622200	-0.39193300

H	4.84918000	1.67489600	1.33297300
H	5.85330000	2.93089900	0.59203900
H	1.45818900	6.72955500	1.66238800
H	3.44962400	4.89143200	-1.66185900
H	-3.44968900	-4.89126800	-1.66174300
H	-1.45824100	-6.72946000	1.66246000
C	3.66257300	6.94480100	0.06937300
C	-3.66262800	-6.94467900	0.06938500
F	3.32433200	8.01935600	0.81207000
F	3.99503100	7.39671800	-1.16176800
F	4.80132100	6.43949000	0.60866600
F	-4.80175100	-6.43908100	0.60760700
F	-3.99426400	-7.39733600	-1.16172200
F	-3.32484700	-8.01878400	0.81292500

Cartesian Coordinates of 15Ind-c

C	1.05922500	0.96421900	-0.04038200
C	2.57426800	-1.11906800	0.02165800
C	2.28468000	-2.51690300	0.02941000
C	0.88023700	-2.68183300	-0.04004600
C	-1.05922800	-0.96420600	-0.04041400
C	-2.57426900	1.11907800	0.02166800
C	-2.28468600	2.51691300	0.02939900
C	-0.88024100	2.68184600	-0.04002900
C	1.32839700	-0.40266700	-0.03864600
C	0.27632300	-1.37588000	-0.07406900
C	-1.32840100	0.40268000	-0.03865400
C	-0.27632500	1.37589400	-0.07404700
H	1.87041500	1.68227000	0.00240900
H	-1.87041500	-1.68226100	0.00236300
C	3.30518500	-3.58708800	0.06127200
C	3.32832500	-4.59023000	-0.91282600
C	4.27399400	-3.61549000	1.07643300
C	4.28783900	-5.60633500	-0.88756900
H	2.58956100	-4.57245000	-1.70667800
C	5.23777000	-4.62152100	1.13149600
H	4.26062900	-2.84493200	1.83948200
C	5.23220200	-5.60917700	0.13968200
H	5.98034700	-6.39664200	0.17077600
C	0.13860800	-3.94691500	-0.04067000
C	-0.94824900	-4.15012900	-0.91551800
C	0.46776200	-4.99457500	0.83139200
C	-1.66350700	-5.33554400	-0.91595200
H	-1.20889400	-3.37581100	-1.62692500
C	-0.25052100	-6.18625500	0.84918200
H	1.29131500	-4.86956800	1.52285600
C	-1.32364900	-6.36522900	-0.02967400
C	-3.30519300	3.58709800	0.06122500
C	-3.32831500	4.59021700	-0.91289800
C	-4.27402400	3.61552000	1.07636200
C	-4.28782600	5.60632600	-0.88768000
H	-2.58953700	4.57241700	-1.70673700
C	-5.23779900	4.62155400	1.13138500
H	-4.26067600	2.84497700	1.83942700
C	-5.23220700	5.60919200	0.13955300
H	-5.98035000	6.39666100	0.17061900
C	-0.13861000	3.94692700	-0.04063800
C	0.94826600	4.15014100	-0.91546100
C	-0.46777700	4.99458500	0.83142400
C	1.66353300	5.33555100	-0.91587100
H	1.20891600	3.37582900	-1.62687300
C	0.25051600	6.18625700	0.84924100
H	-1.29134700	4.86958000	1.52286800
C	1.32366600	6.36522800	-0.02958800
C	3.83929400	-0.52526000	0.00896400
C	4.92712100	0.02228700	-0.01597400
C	-3.83930000	0.52527700	0.00898300
C	-4.92713600	-0.02225000	-0.01593800
C	6.19818700	0.64284800	-0.04537500
C	6.33070800	2.01440600	0.30212600
C	7.34274900	-0.10146800	-0.42273600
C	7.58737500	2.60031300	0.27362000
C	8.58783600	0.52313600	-0.43972200
C	8.72522600	1.86901300	-0.09296300
H	7.68856000	3.64620800	0.54365700
H	9.44989400	-0.06177300	-0.73247000
C	-6.19819400	-0.64283500	-0.04533900
C	-7.34277700	0.10147900	-0.42263700
C	-6.33067800	-2.01441200	0.30210300
C	-8.58785100	-0.52315300	-0.43963700
C	-7.58733300	-2.60034400	0.27359200
C	-8.72520300	-1.86905000	-0.09294500

H	-9.44993100	0.06174800	-0.73233900
H	-7.68849500	-3.64625000	0.54359200
C	6.24858500	-4.66687800	2.25391600
H	5.97071900	-5.41450100	3.00489400
H	7.24281900	-4.93351800	1.88514300
H	6.32323700	-3.70324300	2.76204300
C	4.30248200	-6.66616600	-1.96425800
H	5.01463000	-7.46078500	-1.73208800
H	3.31521000	-7.12137800	-2.08468300
H	4.58252700	-6.24098600	-2.93362500
C	-6.24864500	4.66693000	2.25377600
H	-5.97087200	5.41465900	3.00468400
H	-7.24289600	4.93343500	1.88495100
H	-6.32321900	3.70333900	2.76199900
C	-4.30245100	6.66613000	-1.96439600
H	-5.01451400	7.46081700	-1.73219600
H	-3.31514900	7.12125200	-2.08490400
H	-4.58260500	6.24095000	-2.93373100
S	10.26925000	2.75207100	-0.08026000
S	-10.26920800	-2.75214800	-0.08027400
C	11.48823500	1.50652800	-0.60607200
H	12.44728900	2.02490500	-0.60724000
H	11.53704300	0.67421100	0.09656900
H	11.28030700	1.14536200	-1.61363800
C	-11.48823700	-1.50658800	-0.60594700
H	-12.44727900	-2.02498500	-0.60713100
H	-11.53704100	-0.67433100	0.09676500
H	-11.28034400	-1.14533300	-1.61348800
C	7.21811600	-1.54764400	-0.82553200
H	6.71575000	-2.13786500	-0.05575600
H	6.61505900	-1.64929400	-1.73307900
H	8.19886900	-1.98701200	-1.01635500
C	5.12421900	2.82266200	0.70213500
H	4.39334900	2.86835700	-0.11067400
H	4.61490300	2.37406800	1.55967600
H	5.40457500	3.84401800	0.96396300
C	-5.12416200	-2.82265400	0.70205300
H	-4.39331600	-2.86830300	-0.11078100
H	-4.61483000	-2.37407600	1.55959200
H	-5.40448600	-3.84402600	0.96385300
C	-7.21818000	1.54768600	-0.82533400
H	-6.71586700	2.13787100	-0.05549600
H	-6.61508600	1.64941900	-1.73284700
H	-8.19894100	1.98703000	-1.01616900
H	-2.48732700	-5.49538800	-1.60102600
H	0.03204000	-6.96079300	1.54942500
H	2.48736700	5.49539500	-1.60092800
H	-0.03205300	6.96079500	1.54948200
O	-2.08900800	-7.48911500	-0.10311000
O	2.08905100	7.48910000	-0.10297700
C	-1.78876200	-8.57413800	0.76419200
H	-1.89468000	-8.28864400	1.81660900
H	-2.51352600	-9.35151200	0.52824400
H	-0.77723700	-8.95818700	0.59197500
C	1.78870200	8.57416200	0.76424100
H	1.89458400	8.28874300	1.81668100
H	2.51343900	9.35155800	0.52828300
H	0.77716500	8.95814200	0.59194700

Cartesian Coordinates of 26Ind

C	0.00001900	1.47315700	0.00000800
C	2.55957400	1.19974700	0.01901300
C	3.38905000	0.03935800	-0.05156100
C	2.55790800	-1.12089900	-0.09711200
C	0.00000000	-1.39443000	-0.00002500
C	-2.55790700	-1.12083800	0.09705800
C	-3.38903100	0.03945600	0.05149500
C	-2.55954000	1.19980800	-0.01907200
C	1.20138700	0.76011100	0.01350600
C	1.20041400	-0.68143200	-0.05019000
C	-1.20041400	-0.68140700	0.05016600
C	-1.20135000	0.76013800	-0.01350200
H	0.00002600	2.55635800	0.00000700
H	-0.00000600	-2.47762800	-0.00004000
C	-4.80068900	0.03990400	0.07300300
C	4.80070800	0.03976400	-0.07308400
C	-6.01420800	0.04143400	0.09188000
C	6.01422800	0.04125700	-0.09196200
C	-7.43660400	0.04409900	0.11543300
C	-8.12918300	-0.88762200	0.92696800
C	-8.15216900	0.98293700	-0.66734600
C	-9.52218800	-0.86586200	0.93688200

C	-9.54474400	0.97088300	-0.62565200
C	-10.23666000	0.05016300	0.16295300
H	-10.06307900	-1.56608300	1.56322900
H	-10.10317200	1.69017600	-1.21381500
C	7.43662400	0.04389500	-0.11550400
C	8.12919700	-0.88779500	-0.92706500
C	8.15219600	0.98269000	0.66733600
C	9.52221100	-0.86608300	-0.93692700
C	9.54476400	0.97063600	0.62563700
C	10.23668200	0.04991400	-0.16297400
H	10.06310600	-1.56634200	-1.56322400
H	10.10318900	1.68994900	1.21378200
C	-3.01074200	2.59340400	-0.06993900
C	-2.42081100	3.50220600	-0.96403900
C	-4.03063400	3.05318100	0.77908800
C	-2.83085600	4.83480600	-1.01943700
H	-1.66090200	3.15028100	-1.65229800
C	-4.44399000	4.38415600	0.75742500
H	-4.48695000	2.36368800	1.47858600
C	-3.83740400	5.26018000	-0.14914100
H	-4.16003600	6.29723400	-0.17946200
C	-3.00752500	-2.51440300	0.16225700
C	-2.39068300	-3.42301400	1.03818000
C	-4.05314400	-2.97438200	-0.65472200
C	-2.79897400	-4.75554300	1.10672400
H	-1.61002000	-3.07092800	1.70270400
C	-4.46577000	-4.30526500	-0.61972600
H	-4.53077000	-2.28493700	-1.33990700
C	-3.83181000	-5.18119000	0.26794400
H	-4.15348000	-6.21819900	0.30857200
C	3.00747600	-2.51448500	-0.16231500
C	2.39063200	-3.42305400	-1.03827900
C	4.05303700	-2.97451500	0.65470200
C	2.79888300	-4.75559400	-1.10683800
H	1.61000300	-3.07092200	-1.70282200
C	4.46561800	-4.30541600	0.61969600
H	4.53066000	-2.28509800	1.33991700
C	3.83167100	-5.18129500	-0.26802400
H	4.15330800	-6.21831000	-0.30866500
C	3.01081100	2.59332500	0.06988100
C	2.42085600	3.50215800	0.96395600
C	4.03071300	3.05309000	-0.77912800
C	2.83089800	4.83474600	1.01933900
H	1.66093200	3.15025300	1.65220200
C	4.44407500	4.38407200	-0.75747600
H	4.48703300	2.36360000	-1.47862300
C	3.83747400	5.26010900	0.14905100
H	4.16010100	6.29717100	0.17935300
C	-5.50210100	4.87890300	1.71536500
H	-6.15696400	4.06745500	2.03916600
H	-5.04659800	5.31204300	2.61255700
H	-6.12193900	5.65421300	1.25843500
C	-2.22628600	5.78545400	-2.02582100
H	-1.19369600	5.51629200	-2.25887700
H	-2.78895200	5.77082300	-2.96561600
H	-2.23402600	6.81361800	-1.65657000
C	2.22636800	5.78549900	2.02564700
H	1.19522100	5.51379800	2.26205600
H	2.79171700	5.77428500	2.96387800
H	2.23011200	6.81300800	1.65447600
C	5.50223400	4.87883300	-1.71535600
H	6.15585600	4.06701600	-2.04071700
H	5.04674600	5.31388600	-2.61162700
H	6.12335700	5.65268400	-1.25768100
C	-5.55293900	-4.80012200	-1.54457600
H	-6.21859400	-3.98880100	-1.84611400
H	-5.12561100	-5.23121800	-2.45650600
H	-6.15692900	-5.57701300	-1.06940100
C	-2.16396600	-5.70577200	2.09457500
H	-1.12432300	-5.43733600	2.29479500
H	-2.69678900	-5.68959900	3.05157600
H	-2.18422800	-6.73430200	1.72680200
C	2.16386800	-5.70575500	-2.09475200
H	1.12381900	-5.43809600	-2.29392600
H	2.69585300	-5.68849600	-3.05220000
H	2.18533800	-6.73449400	-1.72764800
C	5.55270600	-4.80029800	1.54462800
H	6.21932700	-3.98929900	1.84491200
H	5.12535100	-5.22985100	2.45727400
H	6.15567800	-5.57836700	1.07010200
S	-12.03355200	0.07868600	0.22197100
S	12.03357500	0.07835300	-0.22199300

C	-12.43534300	-1.09130100	-1.13413000
H	-13.52402600	-1.14506700	-1.18080500
H	-12.05198100	-0.72734600	-2.08737000
H	-12.03506800	-2.08290400	-0.92288700
C	12.43532800	-1.08997600	1.13554800
H	12.03507100	-2.08183600	0.92548400
H	13.52401000	-1.14366300	1.18233900
H	12.05192500	-0.72487700	2.08833400
C	7.42162600	1.97473500	1.53575700
H	6.74639000	2.60130500	0.94677400
H	6.80189200	1.46466400	2.27950300
H	8.12433500	2.62370700	2.06121400
C	7.37355700	-1.87506600	-1.77933400
H	6.72082300	-2.50976500	-1.17379400
H	6.72737100	-1.36058900	-2.49699400
H	8.06058000	-2.51596500	-2.33459900
C	-7.42160400	1.97500300	-1.53574700
H	-6.74629700	2.60150300	-0.94676900
H	-6.80194400	1.46494900	-2.27956800
H	-8.12431200	2.62404300	-2.06112000
C	-7.37353600	-1.87494400	1.77917300
H	-6.72091200	-2.50969500	1.17356500
H	-6.72723700	-1.36051700	2.49676500
H	-8.06055500	-2.51579200	2.33450300

Cartesian Coordinates of 37BDT

C	-0.27245600	-1.39322000	-0.08606200
C	1.05097900	-0.97735800	-0.07033000
C	1.30754700	0.39595800	-0.10538200
C	0.22301100	1.30986900	-0.13985500
C	-1.10048200	0.89409200	-0.15384900
C	-1.35676500	-0.47960500	-0.13443000
H	1.87115400	-1.68341900	-0.02742300
H	-1.92113100	1.60064800	-0.17435600
C	2.48338800	2.44276600	-0.12016600
C	2.59570900	1.06759200	-0.08991700
S	0.80257000	2.96736600	-0.16985700
C	-2.64504400	-1.15096600	-0.13517400
C	-2.53286800	-2.52601500	-0.09713600
S	-0.85177100	-3.05076700	-0.06025400
C	3.81295500	0.35176600	0.00319300
C	-3.86459800	-0.43304100	-0.12089500
C	-4.88285400	0.22505000	-0.09578000
C	4.82765800	-0.30558300	0.09761200
C	6.03927000	-1.04449800	0.20939100
C	7.11314900	-0.51745700	0.96798800
C	6.16252100	-2.30013200	-0.43196100
C	8.29199800	-1.25351300	1.06242100
C	7.36075000	-3.00145800	-0.31338800
C	8.42922500	-2.48591400	0.42117900
H	9.11604100	-0.86797500	1.65179600
H	7.46643400	-3.96569200	-0.79702900
C	-6.09932100	0.96388900	-0.06450500
C	-6.21141700	2.17124700	-0.79486500
C	-7.19322200	0.47896800	0.69306600
C	-7.41540900	2.87121000	-0.75648600
C	-8.37859500	1.21104600	0.70353200
C	-8.49777400	2.40550100	-0.00869400
H	-7.51822800	3.78965300	-1.32292400
H	-9.22632900	0.84662900	1.27227600
C	6.97940500	0.80561300	1.67758700
H	6.75378800	1.61502400	0.97816000
H	6.15919600	0.78182700	2.40122300
H	7.89864700	1.05581400	2.20966900
C	5.01776100	-2.87636600	-1.22499300
H	4.16406400	-3.09933700	-0.57704900
H	4.66446600	-2.17308100	-1.98402500
H	5.31357200	-3.80164400	-1.72180300
C	-5.05210300	2.69270000	-1.60458100
H	-4.20974100	2.96088900	-0.95893200
H	-4.68538000	1.93875600	-2.30643800
H	-5.33913500	3.58040700	-2.17033000
C	-7.07897000	-0.80138100	1.48020300
H	-6.83002000	-1.64792200	0.83480800
H	-6.28178200	-0.73400700	2.22655000
H	-8.01410600	-1.02498000	1.99616700
S	9.95115600	-3.42953700	0.58447400
S	-10.05192100	3.31009800	-0.00452800
C	10.93299200	-2.72697400	-0.79816600
H	11.09638300	-1.65874700	-0.65595600
H	11.89425900	-3.24262300	-0.78380800
H	10.44174200	-2.90781500	-1.75417700

C	-9.78082000	4.43819500	1.41796500
H	-8.92817300	5.09280300	1.23787000
H	-10.68536100	5.04156900	1.50670400
H	-9.63305000	3.87376200	2.33856800
C	-3.59017600	-3.54634900	-0.10385300
C	-3.48322300	-4.68899500	0.70347300
C	-4.71317500	-3.40424400	-0.93018100
C	-4.47546300	-5.66835300	0.70332200
H	-2.62763100	-4.79815300	1.36090600
C	-5.71822900	-4.37192100	-0.95061300
H	-4.79434300	-2.53486400	-1.57068600
C	-5.58726100	-5.49204700	-0.12539400
H	-6.36955500	-6.24602500	-0.12875900
C	3.54107600	3.46269000	-0.13578900
C	3.40754400	4.63971000	0.61517900
C	4.69196000	3.28463800	-0.91642200
C	4.40026500	5.61940200	0.60507000
H	2.53017300	4.77725600	1.23773200
C	5.69734100	4.25087800	-0.94565900
H	4.79412600	2.38723800	-1.51382500
C	5.53923500	5.40709800	-0.17586700
H	6.32190600	6.16059100	-0.18584200
C	-6.90589700	-4.22798500	-1.87294700
H	-6.72616700	-4.74061600	-2.82413100
H	-7.80637800	-4.66331200	-1.43324100
H	-7.11087200	-3.17970500	-2.09969200
C	-4.34852100	-6.89998900	1.56822800
H	-5.30355400	-7.16128600	2.03115100
H	-4.02689600	-7.76313600	0.97569400
H	-3.61519800	-6.75492700	2.36390600
C	6.91673900	4.06784600	-1.81835100
H	6.78908000	4.57637900	-2.78006000
H	7.81000500	4.48416700	-1.34625200
H	7.10307000	3.01252400	-2.02750300
C	4.24072700	6.88932300	1.40689200
H	5.19760300	7.22748600	1.81178900
H	3.84565600	7.69861000	0.78330800
H	3.54975100	6.75056400	2.24094000

Cartesian Coordinates of 26BDT

C	-1.05166700	-0.84268400	-0.00151300
C	0.23360900	-1.35988900	-0.00556400
C	1.31294000	-0.46472700	-0.02929600
C	1.04986300	0.93517000	-0.01268600
C	-0.23586600	1.45245700	0.00104200
C	-1.31520300	0.55760500	-0.01089200
H	0.41138300	-2.42772800	0.01597300
H	-0.41328500	2.52021400	0.02763500
C	3.50317900	0.36919800	-0.00002500
C	2.72393500	-0.76713700	-0.02471500
S	2.54066500	1.86034500	0.01560500
C	-2.72621000	0.86000600	0.01420600
C	-3.50560900	-0.27616500	0.04604800
S	-2.54244800	-1.76755300	0.04306900
C	4.90173100	0.47442300	-0.03323500
C	-4.90400300	-0.38293300	0.03311900
C	-6.11006700	-0.52085400	0.01118000
C	6.10683800	0.61255200	-0.07260000
C	-7.52045300	-0.67147000	-0.01517100
C	-8.10900500	-1.88513400	0.40793300
C	-8.34022900	0.39426800	-0.46848200
C	-9.49619900	-2.01784900	0.37885600
C	-9.71737400	0.22496100	-0.48209600
C	-10.31035500	-0.97259300	-0.06134000
H	-9.92883100	-2.95336100	0.70833000
H	-10.34517400	1.03870300	-0.82959100
C	7.51816500	0.76457900	-0.11953700
C	8.32573000	-0.30221000	-0.58473300
C	8.10542000	1.98607600	0.29173400
C	9.70735500	-0.12932200	-0.61943900
C	9.49123100	2.11493400	0.23924200
C	10.29795300	1.06541400	-0.20378400
H	10.33702000	-0.93312100	-0.98324200
H	9.95349000	3.04687300	0.54361200
C	-7.24977900	-3.02664100	0.88807600
H	-6.56042500	-3.35759900	0.10560100
H	-6.63613300	-2.72668000	1.74250400
H	-7.86118500	-3.87947000	1.18764600
C	-7.72559200	1.68615300	-0.94104400
H	-7.10097200	2.13891500	-0.16664300
H	-7.07507900	1.51721800	-1.80440400
H	-8.49617300	2.40360200	-1.22756000

C	7.70278200	-1.59257400	-1.05091300
H	7.09045200	-2.04882900	-0.26865600
H	7.03921500	-1.42233600	-1.90408600
H	8.46959800	-2.30835700	-1.35143500
C	7.24781600	3.12763100	0.77376400
H	6.54906500	3.45239700	-0.00303200
H	6.64455600	2.83272700	1.63744300
H	7.86186300	3.98292900	1.05979300
S	-12.08717400	-1.04157700	-0.13252500
S	12.08222100	1.27097800	-0.28989900
C	-12.48172800	-2.71663900	0.46064700
H	-12.15449200	-2.86361700	1.49033100
H	-13.56900100	-2.78666200	0.42239500
H	-12.05538300	-3.48108700	-0.18957700
C	12.57126100	0.66122000	1.37106400
H	12.28566000	-0.38252600	1.50149500
H	13.65778500	0.74590500	1.42031200
H	12.12679400	1.27312500	2.15591000
C	3.27130200	-2.14410100	-0.03227700
C	4.11967100	-2.57763500	0.99171400
C	2.93959300	-3.03493100	-1.06147800
C	4.63165400	-3.87709000	1.00323800
H	4.37012200	-1.89403400	1.79528000
C	3.44809100	-4.33462100	-1.08224500
H	2.29459800	-2.69999500	-1.86688800
C	4.28873300	-4.73908600	-0.04108300
H	4.68415400	-5.75110400	-0.04337900
C	-3.27343200	2.23714500	0.01870300
C	-2.95281700	3.13292600	-1.00992900
C	-4.11100800	2.66620900	1.05331300
C	-3.46094900	4.43279800	-1.01918600
H	-2.31684500	2.80162800	-1.82400200
C	-4.62297400	3.96562200	1.07611600
H	-4.35388600	1.97871100	1.85586600
C	-4.29071700	4.83257500	0.03255600
H	-4.68613000	5.84458700	0.03908100
C	5.50517800	-4.34985000	2.14141600
H	4.89901900	-4.76989300	2.95147600
H	6.19892200	-5.12815200	1.81525800
H	6.08901100	-3.52889200	2.56380000
C	3.12604300	-5.27326700	-2.22105500
H	3.92214800	-5.26441800	-2.97343300
H	3.02076400	-6.30311300	-1.87092900
H	2.19854500	-4.98898400	-2.72252400
C	-3.15044400	5.37716100	-2.15652600
H	-3.96095500	5.38349100	-2.89338800
H	-3.02692000	6.40292900	-1.80026000
H	-2.23593900	5.08690900	-2.67801000
C	-5.48525800	4.43241400	2.22528900
H	-4.87042200	4.83709400	3.03664700
H	-6.17397400	5.22067900	1.91281600
H	-6.07397300	3.61168800	2.64134600
